# Bora, CEP192 and Cenexin regulate distinct Plk1-dependent cell and centrosome cycle transitions

**DOI:** 10.1038/s41467-026-75033-5

**Published:** 2026-06-30

**Authors:** Devashish Dwivedi, Crisálida Borges, Daniela Harry, Luca Cirillo, Patrick Meraldi

**Affiliations:** 1 Department of Cell Physiology and Metabolism, Faculty of Medicine, University of Geneva, Geneva, Switzerland; 2 Translational Research Centre in Onco-haematology, Faculty of Medicine, University of Geneva, Geneva, Switzerland; 3 Institute of Cancer Research, London, UK; 4 Department of Biomedical Sciences, University of Padova, Padova, Italy

**Keywords:** Mitosis, Checkpoints, Cell division

## Abstract

Polo-like kinase 1 (Plk1) regulates multiple steps of the cell and centrosome cycle, including mitotic entry, DNA-damage recovery, centrosome maturation and centriole disengagement. Plk1 activity depends on several independent cofactors, such as the cytoplasmic Bora, and the centrosomal proteins Cep192 and Cenexin. However, whether these Plk1 coactivators differentially regulate the Plk1-dependent processes is unknown. Here, we show that each Plk1 coactivator controls different cell and centrosome cycle transitions in human cells. We find that Plk1 already acts in S-phase, where under the control of Cep192 and Aurora A it promotes replication origin firing. Bora is the main Plk1 activator driving mitotic entry, DNA-damage recovery and centrosome maturation in G2, while centriole disengagement is mainly regulated by Cep192 and Cenexin. Our results thus uncover a complex Plk1 activation regulatory network, in which distinct upstream activators dictate its activity in a context-dependent manner.

## Abstract (web summary)

Different proteins activate the cell-cycle kinase Plk1 at distinct stages of the cell cycle. Here, Dwivedi et al. show how the different activators control Plk1 to coordinate DNA replication, mitotic entry and key steps of the centrosome cycle.

## Introduction

The polo-like kinase 1 (Plk1) is a conserved serine/threonine kinase that controls key steps of the cell and centrosome cycles in animal cells to ensure faithful cell division^1^. In the mammalian cell cycle, Plk1 drives mitotic entry by phosphorylating and activating the Cdc25c1 phosphatase and inhibiting the Wee1 and Myt1 kinases^2–4^, resulting in full Cdk1-Cyclin B activation. Plk1 activity also modulates the DNA damage response, as it is essential for re-entry into the cell cycle after a DNA damage-induced cell cycle arrest^5–8^. At the level of the centrosomes, Plk1 drives the dramatic increase in microtubule nucleation capacity in late G2 phase, called centrosome maturation; moreover, it is required at mitotic exit for centriole disengagement, the loss of the orthogonal arrangement of the two centrioles that allows centrosome duplication in the next cell cycle. Finally, in mitosis itself, Plk1 regulates multiple processes, which include nuclear envelope disassembly, chromosome condensation, spindle assembly and the stability of kinetochore microtubules^1,9–11^.

Plk1 activity and abundance show a complex pattern in time and space. The protein consists of two halves, the N-terminal half with the kinase domain and the regulatory C-terminal half. This latter half contains a polo-box domain, which binds to Plk1 substrates via a phospho-epitope^12,13^. During G1 and S-phase, Plk1 shows little activity, as the C-terminal polo-box domain allosterically inhibits the kinase domain^14,15^. Nevertheless, a centrosome-bound pool has been reported to promote DNA replication^16^. At the onset of G2 phase, Plk1 activity rises sharply^2,17^. During G2, it contributes to recovery from DNA damage, promotes mitotic entry and drives centrosome maturation. Plk1 is activated when the autoinhibitory interaction between the kinase domain and the C- terminal half is disrupted, exposing its activation loop (T-loop) for phosphorylation at Thr-210 by the mitotic kinase Aurora-A for full activation^14,18^. Aurora-A-dependent Plk1 phosphorylation is facilitated in G2 by Bora, a Plk1 activator which forms a tripartite complex with Aurora A and Plk1 to provide interface for phosphorylation^6,8,19–25^. Bora itself is regulated via phosphorylation by Cdk1-Cyclin A, allowing it to bridge Aurora-A and Plk1^20–25^. A second Plk1 activator, the centrosomal protein Cep192, is also thought to bridge Aurora-A and Plk1^26,27^, however, in vitro data based on recombinant proteins suggest that it is not efficient at promoting Thr-210 phosphorylation^20^. Moreover, a second centrosomal protein, Cenexin, has also been proposed to promote Plk1 activity in G2^28–30^. Thus, different Plk1 co-activators co-exist. However, the rationale behind the simultaneous utilisation of multiple activators/adaptors to activate the same kinase, Plk1, remains unclear. Whether they provide redundancy as a fail-safe mechanism, fine-tune Plk1 activity in space and time, or impart functional specialisation by regulating discreet cellular Plk1 pools to control distinct cellular functions, remains unclear.

Here, we show in human cells that the Plk1 activators Bora, Cep192, and Cenexin regulate distinct Plk1-dependent functions during the cell- and centrosome-cycle progression. While all three activators contribute to the overall Plk1 activity, cytoplasmic Bora is the main driver for DNA-damage recovery, mitotic entry, and centrosome maturation in late G2. In contrast, S-phase entry, progression, and replication origin firing depends mainly on activation of Plk1 by the centrosomal protein Cep192. Activation of Plk1 in S-phase depends on Aurora-A, implying that similar to Aurora-A-Bora-Plk1 regulating G2^6,19,21–24^, the Aurora-A-Cep192-Plk1 axis regulates S-phase. Finally, Cep192- and Cenexin-dependent Plk1 activity drive centriole disengagement. These findings reveal that Plk1 activation is not governed by a singular mechanism but instead involves distinct upstream regulators that modulate its activity for specific cell- and centrosome-cycle transitions.

## Results

### Different Plk1 activators regulate the activity of distinct Plk1 populations

**Fig. 1:**
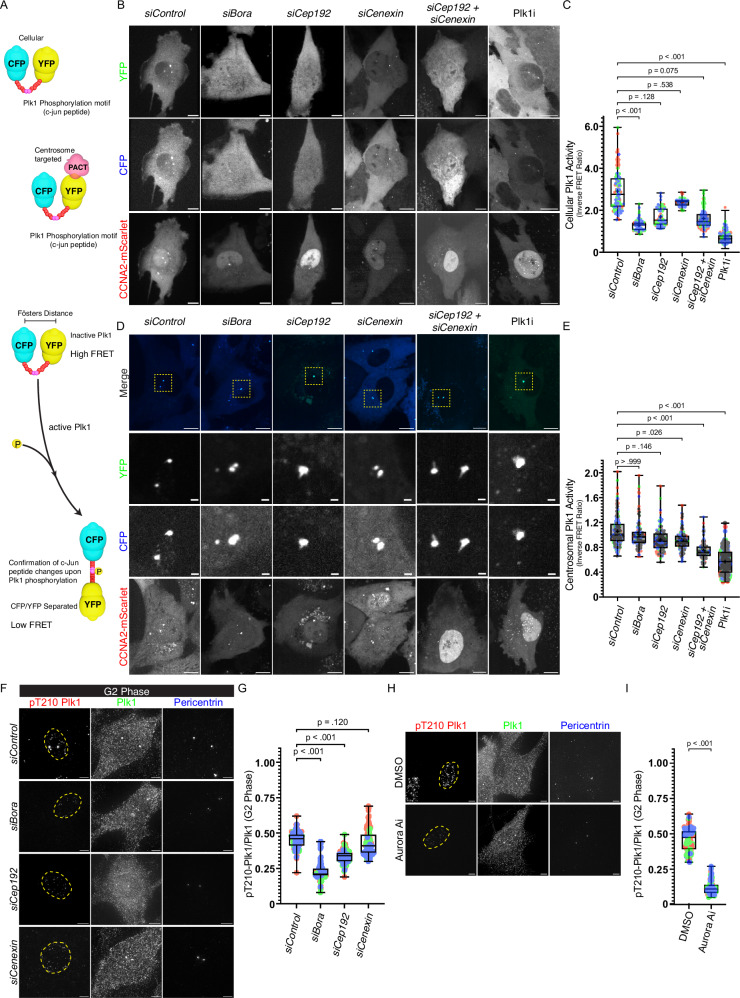
Different Plk1 activators activate distinct Plk1 pools. **A** Diagrammatic representation of the change in confirmation of the sensor that changes FRET efficiency in response to Plk1 activity. **B** Representative images of G2 phase hTERT-RPE1 cells co-expressing cellular Plk1 sensor and CCNA2-mScarlet and treated as indicated. Note that the relative exogenous CCNA2 distribution in G2 cells could vary from cell to cell. **C** Quantification for FRET-based quantification of cellular Plk1 activity from G2 phase cells in (**B**); *N* = 3, *n* = 115: siControl, 124: siBora, 117: siCep192, 122: siCenexin, 132: siCep192 + siCenexin, and 125: Plk1i cells. **D** Representative images of G2 phase hTERT-RPE1 cells co-expressing centrosomal Plk1 sensor and CCNA2-mScarlet and treated as indicated. **E** Quantification for FRET-based quantification of centrosomal Plk1 activity from G2 phase cells in (**D**); *N* = 4, *n* = 183: siControl, 183: siBora, 181: siCep192, 183: siCenexin, 185: siCep192 + siCenexin, and 192: Plk1i cells. **F** Representative images of G2 phase hTERT-RPE1 Plk1-EGFP cells treated with indicated siRNAs and stained with pT210-Plk1, Plk1-GFP (anti-GFP), and pericentrin. The yellow circles represent the nucleus. **G** Quantification for pPlk1/total Plk1 ratio from cells in (**F**)**;** *N* = 3, *n* = 104; siControl, 108: siBora, 107: siCep192, and 103: siCenexin cells. **H** Representative images of G2 phase hTERT-RPE1 Plk1-EGFP cells treated with DMSO/Aurora-Ai and stained with pT210-Plk1, Plk1-GFP (anti-GFP), and pericentrin. The yellow circles represent the nucleus. **I** Quantification for pPlk1/total Plk1 ratio from cells in (**H**); *N* = 3, *n* = 114: DMSO and 119: Aurora Ai treated cells. *p*-values: Kruskal–Wallis test (two-sided) (**C**, **E**, **G**) and two-tailed Mann–Whitney test (**I**) are indicated. The boxes indicate 1st quartile (minima), median (centre), and 3rd quartiles (maxima), and whiskers represent minimum and maximum values. Overlaid dots represent individual observations and the "+" sign represent mean value. Dots of same colour represent cells derived from same experiment. Statistical comparisons not discussed in main text are omitted and are provided in Supplementary Data. Scale bars for whole cells = 5 µm and zoomed insets = 0.5 µm.

The polo-like kinase 1 plays key roles during G2. During this cell cycle phase, it localizes both to the cytoplasm/nucleoplasm and is enriched at centrosomes^31^. To test the contribution of the Plk1 activators, Bora, Cep192 and Cenexin, to the activity of each population, we generated RPE1 cells expressing c-Jun-derived phosphorylation motif based fösters resonance energy transfer (FRET) based Plk1 activity sensors^32,33^ localising either in the cytoplasm/nucleoplasm (untagged) or at centrosomes (tagged with a PACT domain; Fig. 1A). These cells also expressed an inducible Cyclin A2-mScarlet, allowing us to identify G2 cells in an asynchronous population, based on the presence of Cyclin A2 in the cytoplasm^17^. Applying siRNA treatments, we first found that the combined depletion of Bora, Cep192, and Cenexin reduced Plk1 activity in the cytoplasm/nucleoplasm and at centrosomes to the same levels seen after depletion of Aurora-A (the kinase activating Plk1^8^; Fig. S1A–D) or treatment with a Plk1 inhibitor BI2536^34^ (Fig. 1B–E). This confirmed that these three proteins are the main Plk1 activators (Fig. 1B–E). Second, when analysing single or double depletions, we found that Plk1 activity in the cytoplasm/nucleoplasm was primarily dependent on Bora and, to a lesser extent to Cep192 (strong, but nonsignificant trend), whereas the centrosomal Plk1 population was primarily dependent on the centrosomal activators Cep192 and Cenexin (Figs. 1B–E and S1A–D; validation of depletion Fig. S1E–G). Immunoblotting and immunofluorescence indicated that loss of one Plk1 co-activator was not compensated by overexpression of the other ones (Fig. S1E–I).

These results were corroborated by quantitative immunofluorescence with antibodies against Plk1 phosphorylated at Thr-210, a marker for canonical Plk1 activation, which indicated a strong reduction in Thr-210 phosphorylation after Bora depletion and a more modest, but significant reduction after Cep192 depletion (Fig. 1F–I: note that this activated Plk1 form was, consistent with previous studies^35,36^, mostly nuclear and dependent on Aurora-A; Fig. 1H, I). We conclude that Bora regulates primarily Plk1 activity in the cytoplasm/nucleoplasm and that Cep192 and Cenexin regulate primarily Plk1 activity at centrosomes. Nevertheless, the fact that Plk1 activity at either pool could only be suppressed after depletion of all three co-activators suggested continuous exchange of Plk1 between both compartments.

### Plk1 localisation is dynamic on the centrosome

**Fig. 2:**
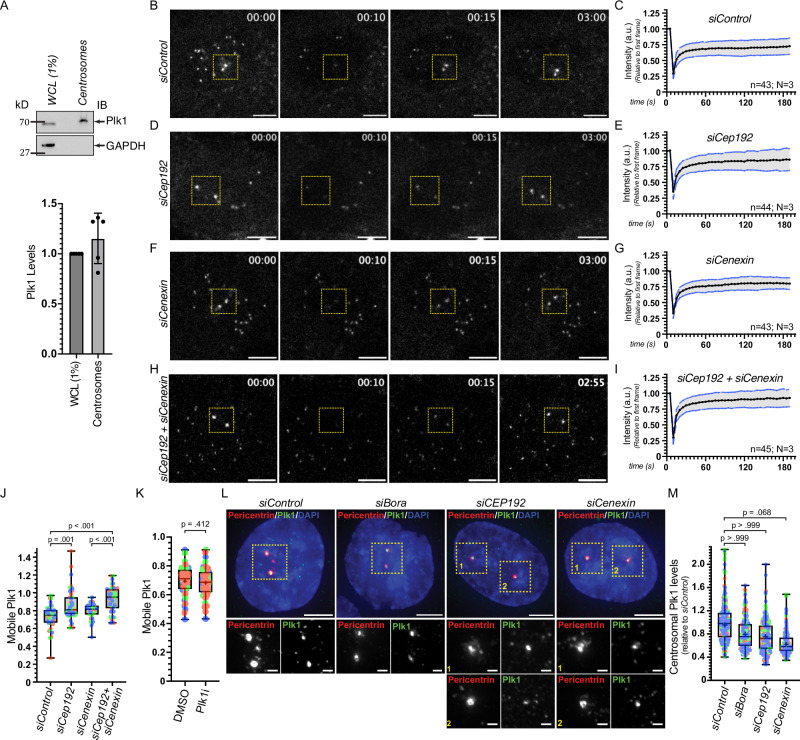
Plk1 dynamically localises on centrosomes. **A** Immunoblot depicting Plk1 levels in whole-cell lysate prepared from 250,000 cells and suspension of same number of centrosomes in Laemmli buffer, and quantification of relative Plk1 levels on centrosomes relative to 1% of the lysate from equivalent cells. Representative time stamp images and quantifications from FRAP movies from G2 phase Plk1-EGFP tagged hTERT-RPE1 cells treated with non-targeting siRNA control (**B** quantification of recovery in (**C**)), Cep192 siRNA (**D** quantification of recovery in (**E**)), Cenexin siRNA (**F** quantification of recovery in (**G**)), and siCep192 + siCenexin (**H** quantification of recovery in (**I**)). Data represented as mean ± SD. **J** Quantification for mobile Plk1 pool on centrosomes under indicated conditions; *N* = 3; *n* = 43, 44, 43, and 45 for siControl, siCep192, siCenexin, and siCep192 + siCenexin, respectively.) **K** Quantification of mobile Plk1 pool on centrosomes after Plk1 inhibition; *N* = 3, *n* = 43, and 53 individual cells for DMSO and Plk1i, respectively. **L** Representative immunofluorescence images of hTERT-RPE1: Plk1-EGFP cells treated with indicated siRNAs and stained for pericentrin (red) and EGFP (green). **M** Quantification for Plk1 levels on centrosomes during G2 phase under the conditions indicated in (**L**)**;** *N* = 5, *n* = 287: siControl, 279: siBora, 276: siCep192, and 287: siCenexin cells. *p*-values: Kruskal–Wallis test (two-sided) (**J**, **M**) and two-tailed Mann–Whitney test (**K**) are indicated. The boxes indicate 1st quartile (minima), median (centre), and 3rd quartiles (maxima), and whiskers represent minimum and maximum values. Overlaid dots represent individual observations and "+" sign represent mean value. Dots of same colour represent cells derived from same experiment. Statistical comparisons not discussed in main text are omitted and are provided in Supplementary Data. Scale bars images: 5 µm and zoomed insets: 0.5 µm.

To better understand the relationship between the centrosomal and the cytoplasmic/nucleoplasmic Plk1 populations, we next quantified their relative abundance. We compared by immunoblotting, Plk1 levels in purified centrosomes isolated from lymphoblastic KE37 cells versus total KE37 cell extracts, as these cells are well established models for centrosome purification^37^, and found that centrosomal Plk1 represents 1–2% of the total Plk1 (Fig. 2A). To next test whether Plk1 exchanges between the cytoplasm/nucleoplasm and the centrosomes, we recorded its recovery dynamics on centrosomes after photobleaching (FRAP) in RPE1 cells expressing endogenously tagged Plk1-EGFP. FRAP-recordings of control-depleted cells in which centrosomal Plk1-EGFP was bleached, indicated the presence of at least two Plk1-EGFP pools: 2/3 of GFP-Plk1 rapidly exchanged with the cytoplasm with (*t*_1/2_ < 10 s), while 1/3 of GFP-Plk1 was immobile over a period of 3 min (Fig. 2B, C, and Supplementary Video 1). To test the contribution of Cep192 and Cenexin to this immobile pool, we depleted either protein or both in combination. The immobile fraction diminished upon depletion of either centrosomal co-activator and disappeared after Cep192/Cenexin co-depletion (Fig. 2D–J and Supplementary Videos 2–4). This change in the mobility was due to a change in Plk1 anchoring rather than Plk1 activity itself, since Plk1 inhibition had no effect on the relative proportion of mobile and immobile Plk1, albeit it affected the recovery kinetics (Figs. 2J, S2A–D, and Supplementary Videos 5, 6). Finally, we also tested whether Bora, Cep192 or Cenexin-depletion affected the overall abundance of Plk1-EGFP at centrosomes and found that all three depletions caused a moderate, but nonsignificant reduction in Plk1 levels (Figs. 2L, M, and S2E, F), consistent with the previous observation that Plk1 activity can regulate its centrosomal abundance^38,39^. We conclude that, in G2, the majority of the centrosomal Plk1 exchanges with the cytoplasm/nucleoplasm, while a small immobile fraction likely binds to Cep192 and Cenexin.

### S-phase progression depends on Cep192, G2 progression on Bora

**Fig. 3:**
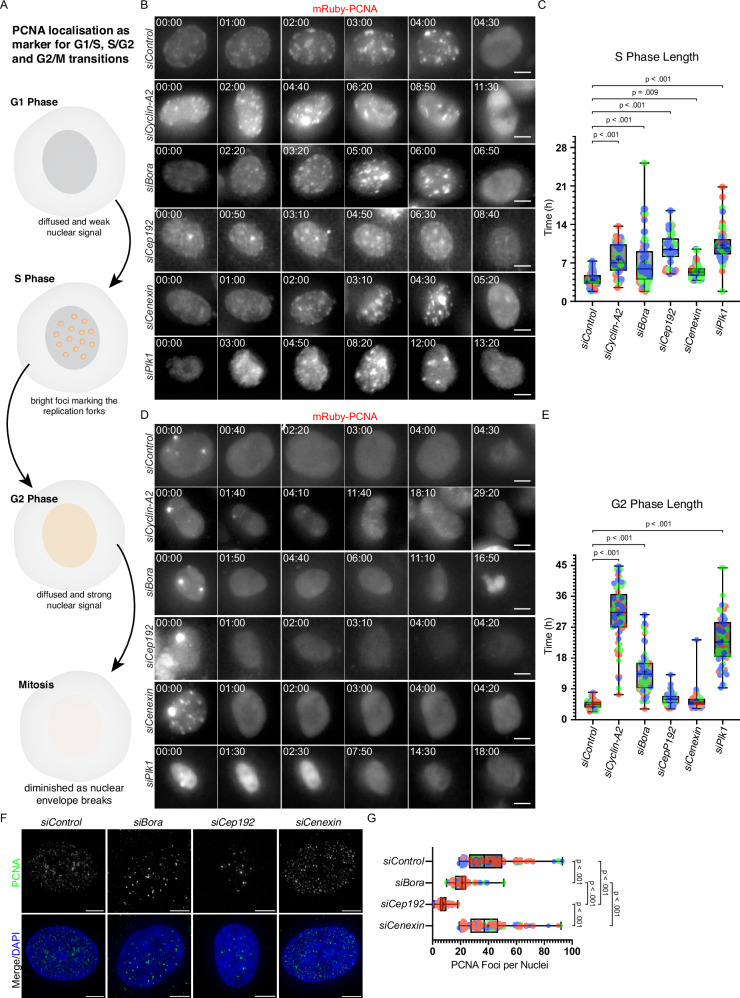
Plk1 activators Bora, Cep192, and Cenexin differentially regulate S and G2 phase progression. **A** Schematic illustrating stage-specific nuclear localization patterns of PCNA across the cell cycle, highlighting transitions from G1 to S phase, progression through G2, and entry into mitosis. **B** Live-cell time-lapse images of hTERT-RPE1: mRuby-PCNA cells treated with indicated siRNAs as they progress through S phase. **C** Quantification for the duration of S phase in cells after depletion of proteins indicated in (**A**); *N* = 3, *n* = 71: siControl, 52: siCyclin A2, 68: siBora, 46: siCep192, 57: siCenexin, and 62: siPlk1 cells. **D** Live-cell time-lapse images of hTERT-RPE1: mRuby-PCNA cells treated with indicated siRNAs as they progress through G2 phase. **E** Quantification for the duration of G2 phase in cells after depletion of proteins indicated in (**D**); *N* = 3, *n* = 83: siControl, 69: siCyclin A2, 69: siBora, 65: siCep192, 83: siCenexin, and 70: siPlk1 cells. **F** Representative images of synchronised S phase RPE1 cells treated with indicated siRNAs and immunolabelled for replication forks using PCNA antibody. **G** Quantification for the number of PCNA foci per cells as proxy for the rate of DNA replication in conditions indicated in (**F**); *N* = 3, *n* = 123: siControl, 122: siBora, 126: siCep192, and 127: siCenexin cells. *p*-values: Kruskal–Wallis test (two-sided) (**C**, **E**, **G**) are indicated. The boxes indicate 1st quartile (minima), median (centre), and 3rd quartiles (maxima), and whiskers represent minimum and maximum values. Overlaid dots represent individual observations and "**+**" sign represent mean value. Dots of same colour represent cells derived from same experiment. Statistical comparisons not discussed in main text are omitted and are provided in Supplementary Data. Scale bars: 5 µm.

Plk1 activity is crucial for progression through different cell cycle stages^1^. We therefore investigated how the depletion of Bora, Cep192, and Cenexin affects the duration of S and G2 phases. We performed long-term time-lapse imaging of live RPE1 cells expressing endogenously tagged mRuby-PCNA, which enabled us to distinguish between the different cell-cycle phases (Fig. 3A). Control-depleted cells completed S phase in 3.88 ± 1.21 h and G2 in 4.59 ± 1.32 h (Fig. 3B–E and Supplementary Videos 7 and 8). Depletion of Cyclin A2, a critical regulator of S- and G-phase and our positive control, strongly delayed S-phase and almost completely blocked the G2/M transition, as 95% of the cells failed to enter mitosis during live-cell imaging duration (Fig. 3B–E and Supplementary Videos 9 and 10). Depletion of any of the three Plk1 co-activator significantly increased S-phase duration, with Cep192 depletion causing the strongest delay (9.57 ± 2.85 h; Fig. 3B, C, and Supplementary Videos 11–13). In contrast, only Bora depletion significantly delayed G2 completion (13.99 ± 6.48 h; Fig. 3D, E, and Supplementary Videos 19–21). Co-depletion of the other co-activators did not further delay S-phase compared to Cep192 depletion, nor did it further extend G2 completion compared with Bora depletion (Fig. S3A–D and Supplementary Videos 14–26). Visual inspection of the mRuby-PCNA signal, which reflects replication origin firing in S-phase^40^, suggested a reduction in this activity. Moreover, previous studies indicated that *Xenopus laevis* Plk1 regulates DNA replication-origin firing in embryonic extracts^41–43^. To test if this is also the case in human somatic cells, we depleted Bora, Cep192, or Cenexin, synchronised the cells at G1/S boundary with the Cdk4/6 inhibitor palbociclib (Cdk4/6i)^44^, released them for 8 h to reach early S phase, stained for PCNA, and counted the number of foci per nucleus. While Cep192 depletion caused roughly a four-fold reduction in PCNA foci, Bora depletion had a mild effect, and Cenexin depletion had none (Fig. 3F, G). Overall, this suggested that only the cytosolic Plk1 activator Bora is rate-limiting for the timely progression of G2 phase, whereas the centrosomal Plk1 activator Cep192 is rate-limiting for replication origin firing and timely completion of S-phase.

### Cep192 controls S-phase progression via Plk1

**Fig. 4:**
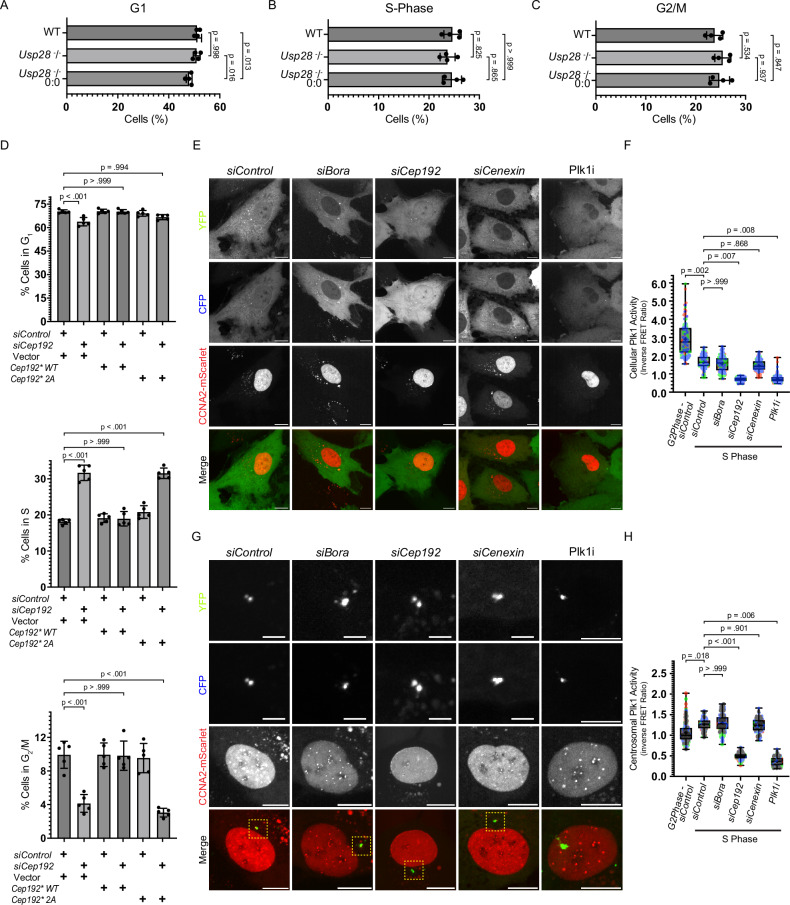
Cep192 but not Bora or Cenexin regulates Plk1 activation during S phase. Cell cycle profile of hTERT-RPE1 (WT), hTERT-RPE1: Usp28^−^/^−^ and hTERT-RPE1: Usp28^−^/^−^ 0:0 cells revealing percentage of cells in G1 (**A**), S (**B**), and G2 (**C**) phases; *N* = 4, *n* = 120,000 cells in each case. **D** Cell cycle profile of hTERT-RPE1 stably expressing either vector control, or RNAi-resistant Cep192 (WT or 2A mutant) and cells treated with control vs. Cep192 siRNA; *N* = 5, *n* = 150,000 cells in each treatment. **E** Representative images of S phase hTERT-RPE1 cells co-expressing cellular Plk1 sensor and CCNA2-mScarlet, treated as indicated. **F** Quantification for cellular Plk1 activity from cells in (**E**); *N* = 4, *n* = 203: siControl, 204: siBora, 215: siCep192, 219: siCenexin, 215: Plk1i cells. The*G2 Phase – siControl* bar shown is replica of siControl data shown in Fig. 1B. **G** Representative images of S phase hTERT-RPE1 cells co-expressing centrosomal Plk1 sensor and CCNA2-mScarlet, treated as indicated. **H** Quantification for centrosomal Plk1 activity calculated from S phase cells represented in (**G**); *N* = 3, *n* = 163: siControl, 187: siBora, 187: siCep192, 184: siCenexin, 178: Plk1i cells. The G2 *phase– siControl* bar shown is replica of siControl data shown in Fig. 1E. *p*-values: one-way ANOVA (**A–D**) and Kruskal–Wallis test (two-sided) (**F**, **H**) are indicated. The boxes in (**F**, **H**) indicate 1st quartile (minima), median (centre), and 3rd quartiles (maxima), and whiskers represent minimum and maximum values. Overlaid dots represent individual observations and "+" sign represent mean value. Dots of same colour represent cells derived from same experiment. Statistical comparisons not discussed in main text are omitted and are provided in Supplementary Data. Scale bars, Images: 5 µm and zoomed insets: 0.5 µm.

The fact that Cep192 depletion reduced the formation of PCNA foci and delayed S-phase completion raised two hypotheses: either that Cep192, possibly in conjugation with Aurora A, is the major activator of Plk1 during S-phase and that this activity is rate-limiting for replication origin firing, or alternatively, given that Cep192 also contributes to centrosome duplication, that the presence of centrosomes are critical for S-phase completion^45,46^. To distinguish between the two possibilities, we generated cells without centrosomes. Specifically, we blocked centrosome duplication with the Plk4 inhibitor centrinone^47^ in RPE1 cells lacking USP28, a component of the mitotic stopwatch checkpoint that induces a p53-dependent cell cycle arrest after centrosome loss^48^. After 1 week of centrinone treatment and a single cell selection, we obtained a RPE1 *USP28*^*−/−*^ cell line devoid of centrosomes (Fig. S4A, B). Flow cytometry indicated that, in contrast to Cep192 depletion, loss of centrosomes did not increase the proportion of RPE1 cells in S-phase (Fig. 4A–D). Strikingly, complementation with an RNAi-resistant wild-type Cep192, but not an RNAi-resistant Cep192 mutant defective in Plk1 binding^49^ (T44A, S995A: hereafter referred as 2A), fully suppressed the S-phase enrichment (Fig. 4D). We conclude that Cep192 drives the S-phase progression via Plk1 activity and independently of centrosomes.

We next quantified the cellular and centrosomal Plk1 activity during S phase after Bora, Cep192, or Cenexin depletion. Using the nuclear localisation of Cyclin A2 as proxy^17^ to identify S phase cells, we found that the cytoplasmic Plk1 activity was much lower in S-phase compared to G2, while the centrosomal Plk1 activity was equivalent (Fig. 4E–H). Consistent with our hypothesis, Plk1 activity in S-phase at either site depended on Cep192, but not on Bora or Cenexin, when measured by FRET (Fig. 4E–H), or by quantifying Plk1 T-loop phosphorylation (Fig. S4C–E). These results therefore confirmed that Cep192 is the principal activator of both cytoplasmic and centrosomal Plk1 during S-phase (Fig. 4E–H).

### The Cep192-Aurora A-Plk1 axis regulates timely S phase progression

**Fig. 5:**
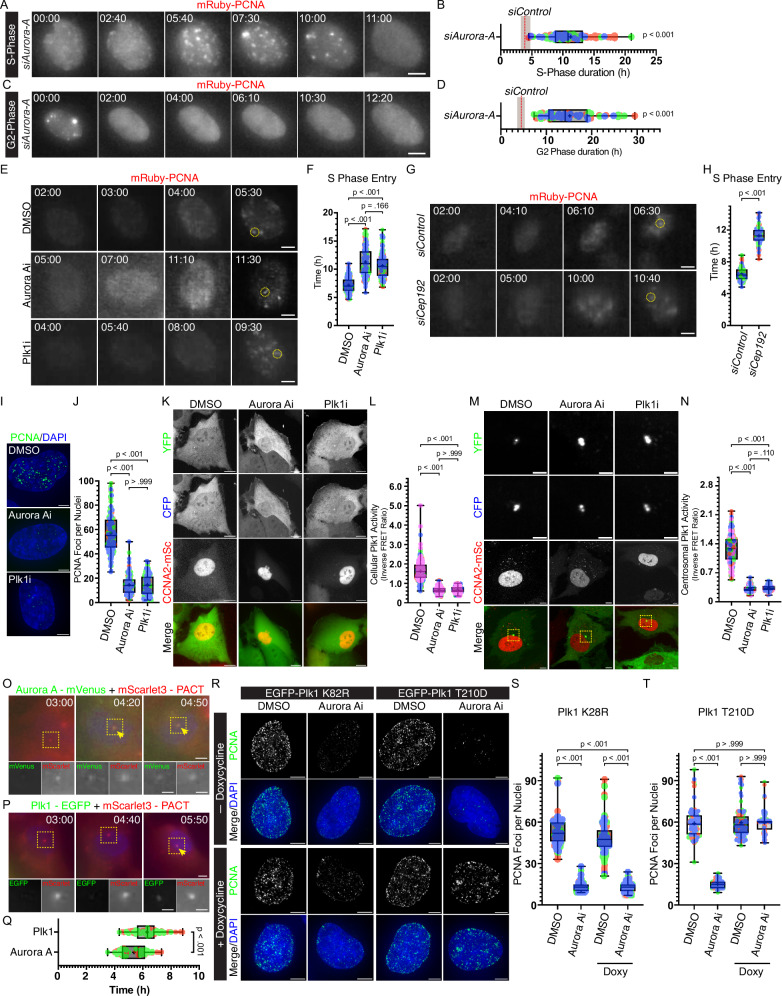
Aurora-Cep192 axis-dependent Plk1 activity regulate S phase onset and progression. Live-cell images of hTERT-RPE1-mRuby-PCNA cells treated with Aurora-A siRNA progressing through S (**A**) and G2 phase (**C**), and quantification of S (**B**) and G2 phase (**D**) duration; *N* = 3, *n* = 68 S-phase and 61 G2 cells. **E** Live-cell images of hTERT-RPE1-mRuby-PCNA cells treated with indicated inhibitors at S-phase entry. **F** Quantification for S phase entry delay; *N* = 3 *n* = 203 DMSO, 206 Aurora Ai and 206 Plk1i cells. **G** Live-cell images of RPE1-mRuby-PCNA cells treated as indicated at S phase entry. **H** Quantification of S-phase entry delay, *N* = 3, *n* = 106 siControl and 100 siCep192 cells. **I** Images of PCNA-labelled S-phase hTERT-RPE1 cells treated as indicated. **J** Quantification of PCNA foci; *N* = 3, *n* = 163 DMSO, 170 Aurora Ai, and 166 Plk1i cells. Images of S-phase hTERT-RPE1 CCNA2-mScarlet cells co-expressing cellular (**K**) or centrosomal (**M**) Plk1 sensor treated as indicated and quantification of cellular (**L**) and centrosomal (**N**) Plk1 activity; *N* = 4, *n* = 245 DMSO, 253 Aurora Ai and 251 Plk1i cells for cellular, *n* = 263 DMSO, 272 Aurora Ai and 287 Plk1i cells for centrosomal activity. Live-cell images of Cdk4/6i synchronised hTERT-RPE1-mScarlet3-PACT cells co-expressing Aurora-A-mVenus (**O**) or Plk1-eGFP (**P**). Brightness, contrast, and gamma function were adjusted to enhance visualisation. **Q** Quantification of centrosomal Aurora A and Plk1recruitment in S phase; *N* = 2, *n* = 101 cells each. **R** Images of PCNA-labelled S-phase hTERT-RPE1 cells expressing indicated transgenes, treated as indicated. **S**, **T** Quantification of PCNA foci in cells expressing K82R or T210D-Plk1; *N* = 3, *n* = 138 (K28R), 126 DMSO, 125 Aurora-Ai, 126 DMSO + doxycycline, and 125 Aurora-Ai + doxycycline (T210D) cells. *p*-values: two-tailed Mann–Whitney (**B**, **D**, **H**,** Q**) and Kruskal–Wallis tests (two-sided) (**F**, **J**,** L**,** N**, **S**, **T**). Boxes indicate 1st quartile, median, and 3rd quartile, and whiskers minimum and maximum values, overlaid dots individual observations, “+” sign mean values, and same colour dots cells same experiment. Statistical comparisons not discussed in main text are omitted and provided in Supplementary Data. Scale bars, images: 5 µm and insets: 0.5 µm.

Finally, our hypothesis predicted that Aurora-A should act upstream of Cep192-Plk1 in terms of S-phase progression, despite the fact that recombinant Aurora-A/Cep192 only showed a limited activity towards Plk1^20^. To detect a role for Aurora A in regulating S phase progression, we measured the duration of S and G2 phases in RPE1 mRuby-PCNA cells and found that Aurora A depletion led to the same long delay in S-phase (11.24 ± 3.54 h vs. 3.88 ± 1.21 h in siControl) and G-phase progression (15.17 ± 5.64 h vs. 4.59 ± 1.32 h in siControl) as seen after Plk1 depletion (Figs. 3A–D, 5A–D, and Supplementary Videos 25–28). Moreover, in cells synchronised in lG1 with the Cdk4/6 inhibitor palbociclib, Aurora A or Plk1 inhibition (Fig. 5E, F, and Supplementary Videos 29–31), or Cep192 depletion (Fig. 5G, H, and Supplementary Videos 32, 33) strongly delayed the start of S phase and reduced the number of replication origin firings (as measured by the appearance and the number of PCNA foci) as compared control-treated or control-depleted cells (Figs. 3F, G, and 5I, J). These results were thus consistent with our hypothesis that Aurora A acts upstream of Cep192-Plk1 axis during S phase. Next. using Cyclin A2 localisation as proxy to identify S phase cells and our Plk1 activity FRET assays, we found that acute Aurora A inhibition in S phase abolished Plk1 activity both on centrosomes and in the cytoplasm/nucleoplasm (Fig. 5K–N). Likewise, acute Aurora A inhibition also reduced Plk1 T-loop phosphorylation in S phase (Fig. S5A, B). Moreover, using synchronized RPE1 expressing either endogenously tagged Aurora-A-mVenus or endogenously tagged Plk1-EGFP together with mScarlet fused to PACT domain (centrosomal localisation), we found that Aurora-A was recruited to centrosomes earlier than Plk1 (Figs. 5O–Q, S5C–F and Supplementary Videos 34, 35). Finally, we generated RPE1 cells expressing inducible exogenous EGFP-Plk1 K82R (catalytically inactive) or EGFP-Plk1 T210D (constitutively active) (expression confirmed by immunoblotting Fig. S5G), induced their expression in cells synchronised with a Cdk4/6 inhibitor, released them in the presence of an Aurora A inhibitor and counted the nuclear PCNA foci by immunofluorescence. While a catalytically inactive Plk1 mutant could not rescue the suppression of PCNA foci caused by Aurora-A inhibition, a constitutively active Plk1 mutant mimicking Aurora-A phosphorylation fully restored the number of PCNA foci in the presence of an Aurora A inhibitor (Fig. 5R–T). Overall, we conclude that Cep192-Aurora-A regulate S-phase entry and progression by activating Plk1 on centrosomes, and that this activity is rate-limiting for the numbers of replication origin firings.

### Bora is essential for DNA-damage recovery

**Fig. 6:**
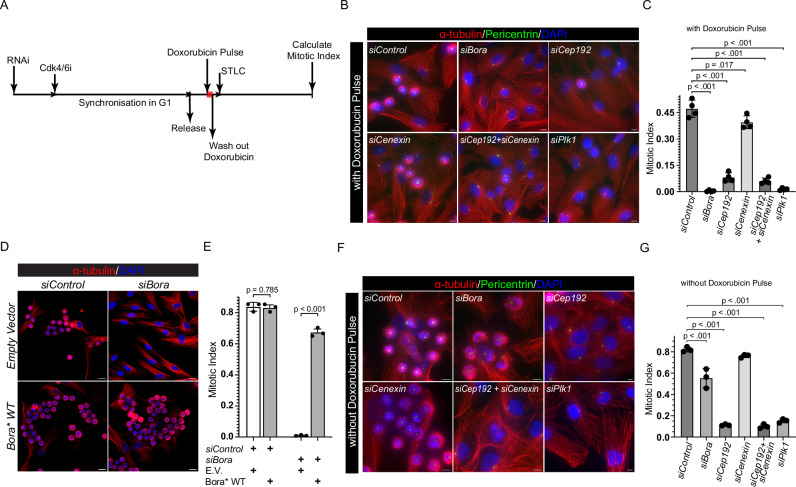
Bora, but not Cep192 or Cenexin, dependent Plk1 activation during G2 is required for checkpoint recovery. **A** Schematic representation of the experimental setup for investigating the role of Plk1 activators in checkpoint recovery. **B** Representative field images of hTERT-RPE1 cells treated with indicated siRNAs and stained for α-tubulin, pericentrin, and DAPI. **C** Quantification representing mitotic index after DNA damage recovery in cells; *N* = 4, *n* = 1136 siControl, 1398 siBora, 976 siCep192, 1359 siCenexin, 1157 siCep192 + siCenexin, and 972 siPlk1 cells. **D** Representative field images of hTERT-RPE1 cells stably expressing either empty vector or RNAi-resistant Bora and treated with Bora siRNA and stained for α-tubulin, pericentrin, and DAPI. **E** Quantification representing rescue in mitotic index after DNA damage recovery in cells after reintroducing RNAi resistant Bora in Bora depleted cells *N* = 3, *n* = 1317 siControl, 1805 siBora, 1365 siControl + Bora* WT, and 930 siBora +  Bora* WT cells. **F** Representative field images of hTERT-RPE1 cells treated with indicated siRNAs and stained for α-tubulin, pericentrin, and DAPI. **G** Quantification of mitotic index in cells without doxorubicin pulse under different depletion conditions; *N* = 3, *n* = 1413 siControl, 838 siBora, 827 siCep192, 1412 siCenexin, 636 siCep192 + siCenexin, and 1803 siPlk1 cells. *p*-values: one-way ANOVA are indicated. Data presented as mean value ± SD. Statistical comparisons not discussed in the main text are omitted; complete results are provided in the Supplementary Data. Scale bars: 5 µm.

In addition to its generic cell cycle roles, Bora and Plk1 are also critical for cell cycle re-entry after DNA damage, as Plk1 directly inhibits the DNA-damage checkpoint effectors 53BP1, p53 and their downstream checkpoint kinases Chk1 and Chk2^8,50–53^. To determine whether Cep192 or Cenexin also contribute to DNA-damage recovery, we synchronised the cells in G1 with a Cdk4/6i, released them for 8 h in the presence or absence of a short pulse of the DNA-damaging agent doxorubicin, and treated them with the Eg5 inhibitor STLC for 16 h to arrest them in mitosis (See scheme Fig. 6A). By determining the mitotic index, we could monitor the ability of cells to recover from a DNA-damage induced cell cycle arrest. Consistent with previous studies^8,24^, Bora depletion specifically abolished mitotic entry after a doxorubicin pulse, indicating a failure to recover from a DNA-damage-induced arrest (Fig. 6B, C; confirmation of DNA damage in Fig. S6A). This defect was rescued by reintroduction of an RNAi-resistant wild-type Bora, confirming RNAi specificity and the essential role of Bora in checkpoint recovery, consistent with previous results^24^ (Fig. 6D, E). In contrast, Cenexin depletion did not prevent mitotic entry (Fig. 6B, C). Cep192 and Plk1 depletion gave inconclusive results, as these perturbations already prevented mitotic entry in the absence of Doxorubicin pulse (Figs. 6F, G, and S6B, C), likely reflecting their critical contribution to S-phase progression (Note that both depletion led to approx. 5-h-long delay in S-phase entry combined with an additional slower S-phase progression, explaining their inability to reach mitosis in the timeframe of our experiment; Fig. 5E, F). We therefore conclude that Bora-dependent Plk1 activity, but not Cenexin, is required for DNA-damage recovery.

### Bora is the major driver of Plk1-dependent centrosome maturation during late G2

**Fig. 7:**
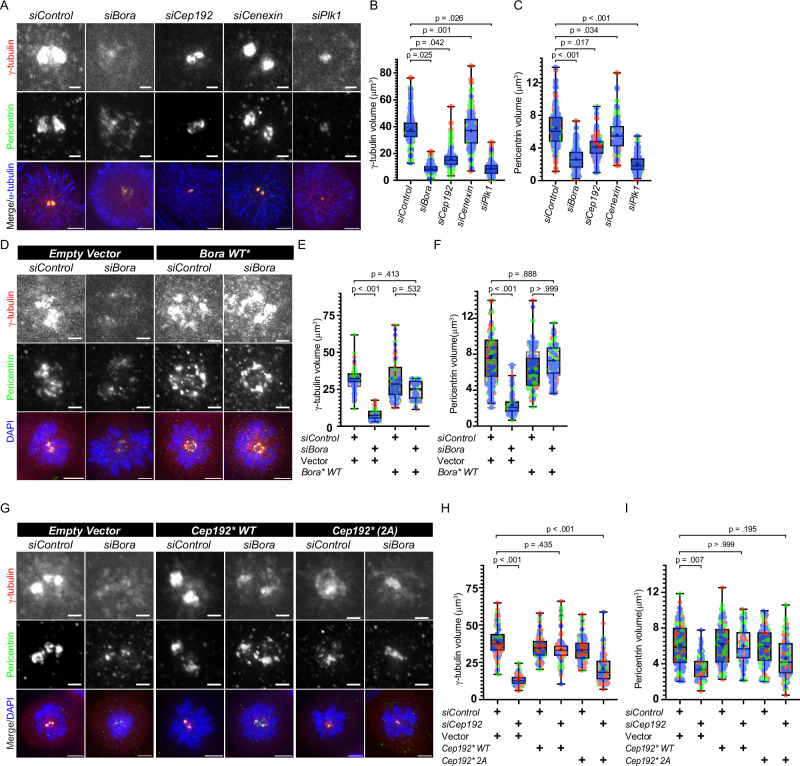
Bora and Cep192 regulate centrosome maturation. **A** Representative images of G2 phase centrosomes stained with γ-tubulin (red) and pericentrin (green) in hTERT-RPE1 cells treated with indicated siRNAs. Quantification for centrosomal levels of γ-tubulin (**B**) and pericentrin (**C**) in cells represented in (**A**)**;** *N* = 3, *n* = (168: siControl, 171: siBora, 177: siCep192, 165: siCenexin, and 168: siPlk1) for γ-tubulin and *n* = (176: siControl, 170: siBora, 179: siCep192, 157: siCenexin, and 183: siPlk1) for pericentrin cells. **D** Representative images of G2 phase centrosomes stained with γ-tubulin (red) and pericentrin (green) in hTERT-RPE1 cells expressing indicated transgenes and treated as indicated. Quantification for centrosomal levels of γ-tubulin (**E**) and pericentrin (**F**) from cells in (**D**); *N* = 3, *n* = (117: siControl, 119: siBora, 118: siControl + Bora* WT, and 120: siBora + Bora* WT) for γ-tubulin and *n* = (117: siControl, 119: siBora, 121: siControl + Bora* WT and 124: siCenexin and 183: siBora + Bora* WT) for pericentrin cells. **G** Representative images of G2 phase centrosomes stained with γ-tubulin (red) and pericentrin (green) in hTERT-RPE1 cells indicated transgenes and treated as indicated. Quantification for centrosomal levels of γ-tubulin (**H**) and pericentrin (**I**) from cells in (**G**)**;** *N* = 3, *n* = (135: siControl, 134: siCep192, 136: siControl + Cep192* WT, 136: siCep192 + Cep192* WT, 135: siControl + Cep192* 2A, and 134: siCep192 + Cep192* 2A for γ-tubulin) and (135: siControl, 134: siCep192, 136: siControl + Cep192* WT, 136: siCep192 + Cep192* WT, 135: siControl + Cep192* 2A, and 134: siCep192 + Cep192* 2A for pericentrin) cells. *p*-values: Kruskal–Wallis test (two-sided) are indicated. The boxes indicate 1st quartile (minima), median (centre) and 3rd quartiles (maxima) and whiskers represent minimum and maximum values. Overlaid dots represent individual observations, and the "**+**" sign represent the mean value. Dots of the same colour represent cells derived from the same experiment. Statistical comparisons not discussed in the main text are omitted and are provided in Supplementary Data. Scale bars, images: 5 µm and zoomed insets: 0.5 µm.

As cells prepare for mitotic entry in late G2, Plk1 drives centrosome maturation via the recruitment of pericentrosomal matrix (PCM) proteins, such as pericentrin (PCNT) and γ-tubulin, to increase the microtubule-nucleation capacity of the future spindle poles^54–57^. To identify the contribution of each of the Plk1-adaptors in this process, we monitored the total volume of γ-tubulin and PCNT on the early prometaphase spindle poles after Bora, Cep192, or Cenexin depletion. Whereas Bora-depletion reduced the levels of γ-tubulin and PCNT on spindle poles as severely as Plk1 depletion, Cep192 depletion only caused an intermediate (50%) reduction, and Cenexin depletion had no effect (Figs. 7A–C and S7A–C). Consistent with these findings, expression of RNAi-resistant Bora and Cep192 restored pericentrin and γ-tubulin levels on centrosomes (Fig. 7D–I). In contrast, the Cep192 2A mutant that cannot interact with Plk1 partially restored the recruitment of those pericentrosomal components (Fig. 7G–I), indicating that Cep192 contributes to PCM assembly both in a Plk1-dependent and independent manner. Overall, these data imply that during G2, the pool of Plk1 activated by Bora is the key regulator of centrosome maturation.

### Cep192 and Cenexin drive centriole disengagement

In cells exiting mitosis, Plk1 regulates the first step of centrosome duplication by inducing centriole disengagement during mitotic exit^58–60^. In our previous work, we showed that incomplete Plk1 activation caused by mild DNA replication stress leads to premature centriole disengagement in G2^61^. We hypothesized that such partial Plk1 activity mimics the condition found during mitotic exit, when Plk1 activity declines^62^. To analyse how this process is controlled, we depleted each of three Plk1 activators, and examined the centriole engagement status in G2 phase using ultrastructure-expansion microscopy (U-ExM), as previously described^61^.

**Fig. 8:**
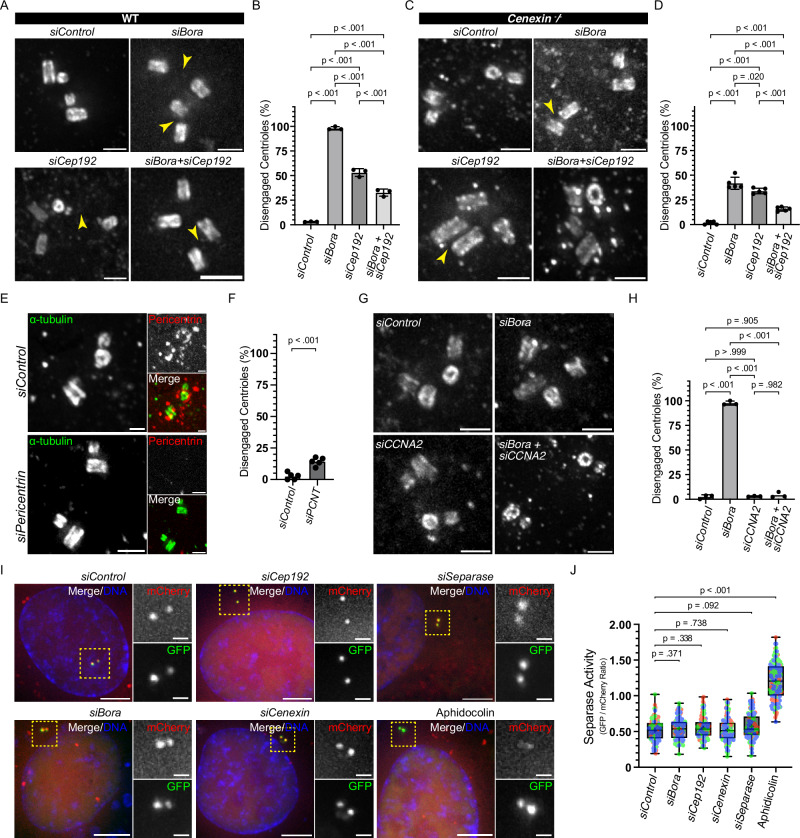
Cenexin-dependent Plk1 activation drives centriole disengagement. **A** Representative U-ExM images of centrioles in G2 phase hTERT-RPE1 cells stained for α-tubulin and treated as indicated. **B** Quantification of percentage G2 cells with disengaged centrioles; *N* = 3, *n* = 96: siControl, 98: siBora, 100: siCep192, and 104: siBora + siCep192 cells. **C** Representative U-ExM images of centrioles in G2 phase hTERT-RPE1 Cenexin^−^^/^^−^ cells stained for α-tubulin and treated as indicated. **D** Quantification of percentage of G2 cells with disengaged centrioles; *N* = 5, *n* = 166: siControl, 149: siBora, 138: siCep192, and 145: siBora + siCep192 cells. **E** Representative U-ExM images of centrioles in G2 phase hTERT-RPE1 cells stained for α-tubulin and treated as indicated. The cells were also counterstained for pericentrin to confirm depletion. **F** Quantification of percentage of G2 phase hTERT-RPE1 cells with disengaged centrioles in their centrosomes; *N* = 5, *n* = 112: siControl and 129: siPericentrin cells. **G** Representative U-ExM images of centrioles in G2 phase hTERT-RPE1 cells stained for α-tubulin and treated as indicated. The arrowheads in (**A**, **C**, **E**, **H**), if indicated, represent disengaged mother-daughter centriole pair. **H** Quantification of percentage of G2 cells with disengaged centrioles; *N* = 3, *n* = 79: siControl, 81: siBora, 101: siCCNA2, and 104: siBora + siCCNA2 cells). **I** Representative images of hTERT-RPE1 expressing centrosomal separase sensor and treated as indicated. **J** Quantification for centrosomal activity of separase in conditions depicted in (**I**); *N* = 3, *n* = 142: siControl, 144: siBora, 134: siCep192, 152: siCenexin, 152: siSeparase, and 147: aphidicolin treated cells. *p*-values: one-way ANOVA (**B**, **D**,** H**), two-tailed Student’s *t* test (**F**), and Kruskal–Wallis test (two-sided) (**J**) are indicated. The boxes in (**F**, **H**) indicate 1st quartile (minima), median (centre) and 3rd quartiles (maxima) and whiskers represent minimum and maximum values. Overlaid dots represent individual observations and “+” sign represent mean value. Dots of same colour represent cells derived from same experiment. Scale bars, Images: 5 µm and zoomed insets: 0.5 µm.

Strikingly, Bora depletion led to premature centriole disengagement in nearly all cells (98.10 ± 1.65% vs. 1.76 ± 1.61% in control-depleted cells), while Cep192 depletion produced an intermediate effect (53.10 ± 3.95% of cells with disengaged centrioles) (Fig. 8A, B), and Cenexin loss did not induce centriole disengagement (Fig. 8C, D). Premature centriole disengagement was not due to DNA replication stress, since, unlike the DNA-polymerase inhibitor aphidicolin, none of the depletions increased typical DNA replication stress markers, such as 53BP1 or γ-H2AX foci (Fig. S8A–C). Neither did Bora nor Cep192 depletion induce premature centriole disengagement purely by lengthening G2 or impairing centrosome maturation, since neither depletion of Cyclin A2, which also prolongs G2, nor depletion of pericentrin, a critical pericentriolar material scaffold protein required for centrosome maturation, resulted in strong premature centriole disengagement (Fig. 8E–H). Rather, our results suggested that in conditions with partial cytoplasmic Plk1 activity but high centrosomal Plk1 activity, as seen after Bora depletion (Fig. 1), centrioles disengage. To test this hypothesis, we co-depleted Bora and CEP192 either in WT RPE1 cells or in RPE1 *Cenexin*^*−/−*^ knock-out cells (to avoid the technical challenge of identifying properly staged G2 phase cells in triple siRNA treatment combined with expansion microscopy). Loss of either centrosomal Plk1 activator reduced centriole disengagement in a Bora siRNA background, and depletion of both almost completely abolished it (Fig. 8C, D). We conclude that centriole disengagement is predominantly mediated by the centrosomal Plk1 activators Cep192 and Cenexin, and that the conditions that maintain centrosomal Plk1 activity while simultaneously reducing cytoplasmic Plk1 activity (Bora depletion) in G2 are sufficient to triggers premature centriole dis-engagement.

Next, we quantified the proteolytic separase activity on the centrosomes in the depletions leading to premature centriole disengagement. Indeed, together with Plk1, separase plays a key role in centriole disengagement during telophase^63–65^ and in the case of premature centriole disengagement in G2 following mild replication stress^61^. Using an established separase activity sensor^66^, we monitored separase activity on centrosomes during G2 phase, and found that, unlike mild replication stress induced by low doses of aphidicolin (positive control)^61^, none of the tested conditions upregulated separase activity (Fig. 8I, J). Thus, we conclude that centriole disengagement observed after Bora and Cep192 depletion solely rely on partial Plk1 activity.

## Discussion

Here, we investigated how the different Plk1 co-activators regulate Plk1 activity in time and space and whether they differentially control the Plk1-dependent transitions of the cell- and centrosome-cycle. In terms of spatial regulation, our results indicate that the centrosomal and cytoplasmic Plk1 can largely exchange, even though a restricted Plk1 pool at centrosomes exists. At the functional level, we demonstrate that the Plk1-dependent cell- and centrosome-cycle steps are differentially regulated by each Plk1 co-activator. We uncover that S-phase entry, replication-origin firing and S-phase progression specifically depends on the centrosomal activator Cep192 and Aurora-A, which initiate Plk1 activation both at centrosomes and the cytoplasm. Cytoplasmic Bora is the main driver for mitotic entry, DNA-damage recovery and centrosome maturation in G2, while centriole disengagement, which normally occurs at mitotic exit, is controlled by the centrosomal co-activators Cep192 and Cenexin.

Our results indicate that at the spatial level, the different Plk1 populations in G2 exist as a mix of a large interchangeable population combined with a restricted pool at centrosomes. In G2, Bora is the main activator of the large cyto/nucleoplasmic Plk1 pool, while Cep192 and Cenexin are mostly responsible for the activity of the centrosomal Plk1 pool, which comprises roughly 1% of total Plk1 (assuming a cell diameter of 10 µm and centrosome diameter of 800–1000 nm, this implies a 10–20 fold enrichment). This explains how Bora-depletion can strongly reduce the cyto/nucleoplasmic Plk1 activity, without majorly affecting centrosomal Plk1 activity, as Cep192 and Cenexin may suffice to activate this smaller Plk1 population. Nevertheless, the Bora-dependent Plk1 activity is required for centrosome maturation, suggesting that Plk1 might partially phosphorylate pericentriolar material in the cytoplasm. Conversely, cyto/nucleoplasmic Plk1 activity depends in S-phase entirely and in G2 partially on the centrosomal Cep192, inferring a rapid Plk1 exchange. Our FRAP experiments confirm the ability of centrosomal Plk1 to exchange with the cytoplasm, consistent with previous work^39,67^, but also point to an immobile Cep192/Cenexin-dependent fraction, which has been seen in some but not all previous studies^38,39^, possibly due to the use of an exogenously tagged Plk1 version^39^. This immobile fraction points to a partial separation of the different Plk1 pools. This agrees with previous studies, which indicated that DNA-damage recovery is under the control of cytoplasmic Plk1, but not nuclear Plk1, or a Plk1 version unable to diffuse from centrosomes^68^; or that kinetochore-microtubule attachments are regulated specifically by a kinetochore-bound form of Plk1^69^.

At the temporal level, we find a much clearer distinction, as Plk1 activators differentially regulate Plk1-dependent cell- and centrosome cycle transitions. While Bora is the main driver of mitotic entry, DNA-damage recovery, and centrosome maturation in G2, we uncover a major role of Cep192 in S-Phase progression, while centriole disengagement in late mitosis depends on Cep192, and to a lesser extent on Cenexin. Earlier studies reported negligible Plk1 activity during early cell cycle phases and proposed that Plk1 activation is restricted to G2, requiring S-phase completion^2,17,19^. Other studies, however, provided evidence for a role of Plk1 in DNA replication^5,41–43^. Our data indicate that human cells exhibit low, but functionally significant Plk1 activity in S-Phase that drives DNA replication-origin firing. This S-phase activity depends on Cep192 and Aurora-A but does not require centrosomes themselves. So far, Cep192 was mostly thought to recruit Aurora A and Plk1 at centrosomes to promote centrosome maturation and spindle formation^26,49^, while Aurora-A had been reported in cancer cells to regulate replication fork initiation, but in a kinase-independent manner^70^. Here, we show in non-transformed cells that Cep192 and Aurora-A kinase activity plays a key role in promoting S-phase entry and progression via Plk1 activation. In vitro Cep192 exhibits a limited ability in promoting Aurora-A-dependent Plk1 phosphorylation^20^, but our in cellulo results indicate that it is nevertheless essential for Plk1 T210 phosphorylation in S-phase, and our Plk1 T210D rescue experiment implies that this phosphorylation event drives S-phase progression. This suggests two non-exclusive possibilities. The Aurora-A/Cep192 complex could phosphorylate Plk1 with low but sufficient efficiency during S phase before being Cep192 is displaced by Bora as cells progress into G2, consistent with the observation that Bora and Cep192 compete for the same binding site on Aurora-A^22^. Alternatively, Cep192-dependent activation of Plk1 in S phase could require an additional centrosomal/pericentrosomal interactor that is still present in acentriolar cells, thereby explaining the persistence of Cep192 dependence in our acentriolar system. In either scenario, the rise in Bora levels during G2^19^ would favour displacement of Cep192 from Aurora-A, and promote Bora-dependent Plk1 activation.

In G2, Bora is the main Plk1 activator. Our data align with previous findings on the critical role of Plk1 in DNA damage checkpoint recovery, centrosome maturation, and mitotic entry in late G2^8,25,56^. We observed a similar magnitude of impaired centrosome maturation after depletion of either Bora or Plk1, implying that Cep192 and Cenexin cannot substitute for cytoplasmic Bora during this process, despite their centrosomal localization. This suggest that full centrosome maturation requires Bora-activated Plk1 to phosphorylate (peri-)centrosomal components in the cytoplasm. Cep192 also contributes to centrosome maturation, but to a lesser extent. Moreover, our RNAi complementation experiments indicate that Cep192 contributes to centrosome maturation both in a Plk1-dependent and Plk1-independent manner, consistent with previous studies^71–74^.

Finally, Plk1 regulates centriole disengagement^58,60^, a critical step for licensing centrioles for the next duplication round. This appears to be the only step that depends on the Cep192- and Cenexin-dependent centrosomal Plk1 pool. Our data indicates, that Cep192 is the primary activator for centrosomal Plk1 and that Cenexin only has limited contribution towards fine-tuning centrosomal Plk1 activity. As such, Cenexin might not be a cell cycle stage-specific Plk1 activator but might more provide a localized Plk1 activity. This aligns with a recent studies revealing how Cenexin fines-tunes centrosome maturation on old and new centrosomes through Plk1 activity^30,75^. Centriole disengagement, which normally occurs during telophase, thus appears to be predominantly regulated by Cep192- and Cenexin-dependent Plk1 pools, which may become rate-limiting once Bora is degraded in mitosis^19,76,77^. The fact that centriole disengagement does not require the high Bora-dependent Plk1 activity is also consistent with our previous finding that the Plk1 activity threshold required for centriole disengagement is much lower^61^ than for other Plk1-dependent mitotic steps. A second striking observation is that in Bora-depleted cells, nearly every G2 centriole pair is disengaged. While Bora depletion also prolongs G2 phase and impairs centrosome maturation, these two defects did not suffice to induce centriole disengagement on their own, since neither Cyclin A2 (prolonged G2) nor pericentrin depletion (impaired centrosome maturation) induced premature centriole disengagement. Rather, we propose that premature centriole disengagement instead arises from sustained Cep192 and Cenexin-dependent centrosomal Plk1 activity combined with reduced cyto/nucleoplasmic Plk1 activity during a prolonged G2 phase, consistent without previous data showing that forcing cells to rapidly enter mitosis suppresses Plk1-dependent premature centriole disengagement in G2^61^.

**Fig. 9:**
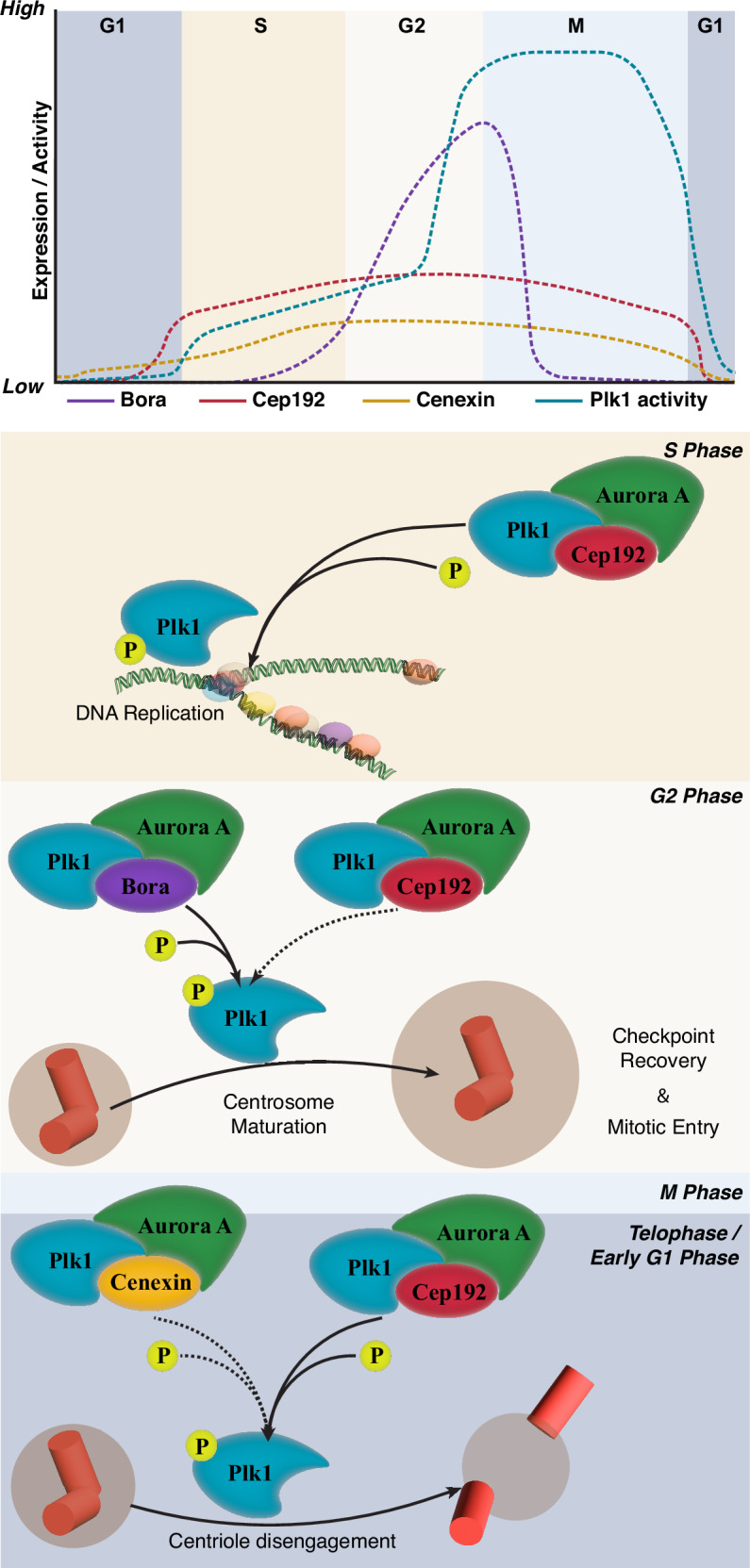
Sequential and combinatorial activation of Plk1 by Cep192, Bora, and Cenexin. Schematic illustration showing Bora, Cep192, and Cenexin display cyclin-like, partially overlapping expression across the cell cycle and generate a progressive increase in Plk1 activity to facilitate mitotic entry. While Cep192 triggers Aurora A-dependent Plk1 activation to facilitate S-phase onset and progression, Bora amplifies and drives full Plk1 activation required for mitotic entry and progression. At the end of mitosis, Cep192 and Cenexin regulate centrosomal Plk1 activity-dependent centriole disengagement. Dashed arrows denote a non-dominant contribution of the respective activator to Plk1 activation at the respective cell cycle stage.

Overall, we demonstrate that Plk1 activity is regulated by Bora and Cep192 and to a lesser extent, Cenexin, with each cell cycle or centrosome cycle transition requiring a specific set(s) of Plk1 activators. This stage-specific regulation is reminiscent of the Cdk–cyclin system, where cyclin expression controls Cdk temporal activity and drive cell cycle progression, suggesting that Plk1, like Cdks, rely on a sequential activation mechanism to coordinate stage-specific functions. Similar to D-type cyclins, which are present through several cell cycle stages, Cep192 is present early and persists until mitotic exit, while Bora resembles more Cyclin-E, as it plays a crucial role during a particular cell transition, G2 and mitotic entry (see model in Fig. 9). This differential regulatory network can, however, induce genetic instability, as exemplified Bora depletion, uncoupling the cell cycle and the centrosome cycle progression, where the mitotic entry is delayed (retarded cell cycle) while the centriole disengagement is promoted (accelerated centrosome cycle) simultaneously, resulting in a pronounced desynchrony between the two otherwise synchronous processes. It therefore will be exciting to explore in the future, whether such type of asynchronies can also be observed in pathological conditions, such as genetically instable cancer cells.

## Methods

### Cell culture and drug treatments

hTERT-RPE1 cells (ATCC: CRL-4000), hTERT-RPE1 Plk1-EGFP, hTERT-RPE1 USP28^−/−^ (kind gift by Arshad Desai, University of California, San Deigo, United States)^48^, h-TERT RPE1 Cenexin^−/−^ (kind gift by gift of Brian Tsou, Sloan Kettering Institute, United States)^78^, hTERT-RPE1 FRT/TR (kind gift by Johnathan Pines, Institute of Cancer Research Cambridge, United Kingdom), hTERT-RPE1 mRuby-PCNA (kind gift by Alexis Barr, Imperial College London, United Kingdom)^79^, hTERT-RPE1 Aurora A-mVenus (kind gift by Catherine Lindon, University of Cambridge, United Kingdom), hTERT-RPE1 Cyclin A2-mScarlet + Plk1-FRET sensor, hTERT-RPE1 Cyclin A2-mScarlet + Plk1-FRET-PACT sensor, hTERT-RPE1 centrosomal separase sensor, hTERT-RPE1 EGFP-Plk1 K82R, hTERT-RPE1 EGFP-Plk1 T210D, hTERT-RPE1 EGFP-Cep192* WT, hTERT-RPE1 EGFP-Cep192* 2A (T44A, S995A), hTERT-RPE1 EGFP-Bora* WT, hTERT-RPE1 Plk1-GFP + mScarlet3-PACT, hTERT-RPE1 Aurora A-mVenus + mScarlet3-PACT cells were cultured in Dulbecco’s Modified Eagle’s Medium (Thermo-Fisher Scientific: 61965-026) supplemented with 10% foetal calf serum (FCS) [Regular: (Thermo-Fisher Scientific: A5256701) or Tet-System Approved: (Thermo-Fisher Scientific: A4736201) as required] and 100 U/ml of each penicillin and streptomycin (P/S) (Thermo-Fisher Scientific: 15140122) at 37 °C, 95% relative humidity and 5% CO_2_ in humidified CO_2_ incubator. Human lymphoblastic KE37 cells (DSMZ: ACC 46) were cultured in RPMI 1640 medium (Thermo-Fisher Scientific: 72400-013) supplemented with 10% foetal calf serum (FCS) (Thermo-Fisher Scientific: A5256701) and 100 U/ml of each penicillin and streptomycin (P/S) (Thermo-Fisher Scientific: 15140122) at 37 °C, 95% relative humidity and 5% CO_2_ in humidified CO_2_ incubator. All cell lines were routinely tested for mycoplasma contamination by PCR. For live-cell imaging, cells were cultured in Leibovitz’s L-15 medium without phenol red (Thermo-Fisher Scientific: 21083-027) with 10% FCS and 100 U/ml P/S at 37 °C. Cells were treated with following inhibitors/drugs to inhibit/induce indicated proteins as per experimental requirement, Cdk1: 9 µM RO3306 (Sigma Aldrich: SML0569), Plk1: 10 nM BI2536 (Selleck Chemicals: S1109), Aurora A: 0.2 µM MLN8237 (Selleck Chemicals: S1133), 300 nM Plk4: Centrinone (Tocris Bioscience: 5687), Eg5: 10 µM (+)-S-Trityl-L-cysteine/STLC (Sigma Aldrich: 164739), DNA polymerase (to induce mild replication stress): 400 nM aphidicolin (Sigma Aldrich: A0781), 0.2 µM doxorubicin: (to induce DNA damage) (Sigma Aldrich: D1515-10MG), 1 µg/ml nocodazole (Sigma Aldrich: M1404-2MG), 1 µg/ml Cytochalasin D (Sigma Aldrich: C8273) and 1 µg/ml doxycycline (Sigma Aldrich: D3447-1G). To label microtubules and DNA in live cell imaging 50 nM SiR-tubulin (Cytoskeleton Inc: CY-SC002), SiR-DNA (Cytoskeleton Inc: CY-SC007), and SPY505-DNA (Cytoskeleton Inc: CY-SC101) were used according to manufacturer’s instructions.

### Preparation of stable cell lines

hTERT-RPE1 separase sensor cells were prepared by transfecting hTERT-RPE1 FRT/TR cells with 0.5 µg separase sensor (pcDNA5-FRT/TO-mCherry-Scc1^(142-467)-ΔNLS^-EGFP-PACT)^66^ and 4.5 µg pOG44 plasmids using X-tremeGENE™ 9 (Merck: XTG9-RO) transfection reagent according to manufacturer’s instructions. The transfected recombinants were selected with 200 µg/ml Hygromycin-B (Invivogen: ant-hm-5) in DMEM with 10% FCS and 100 U/ml P/S. Similarly, hTERT-RPE1 EGFP-Plk1 K82R, hTERT-RPE1 EGFP-Plk1 T210D cells were prepared by transfecting hTERT-RPE1 FRT/TR cells with 0.5 µg plasmid containing EGFP tagged Plk1 mutants (pcDNA5-FRT/TO-EGFP-Plk1 K82R or pcDNA5-FRT/TO-EGFP-Plk1 T210D) and 4.5 µg pOG44 plasmids as described above, and transfected recombinants were selected with 200 µg/ml Hygromycin-B in DMEM with 10% FCS and 100 U/ml P/S.

A two-tiered approach was used to prepare hTERT-RPE1 Cyclin A2-mScarlet + Plk1-FRET sensor and hTERT-RPE1 Cyclin A2-mScarlett + Plk1-FRET-PACT (Centrosome localised) sensor cells, where first hTERT-RPE1 FRT/TR cells were transfected with 0.5 µg Cyclin A2 (pcDNA5-FRT/TO-Cyclin A2-mScarlet) and 4.5 µg pOG44 plasmids as described above to generate hTERT-RPE1 Cyclin A2-mScarlet cells. These cells were later transfected again with either Plk1-FRET sensor c-jun substrate plasmid^32^ (Addgene Plasmid #45203) or c-jun-based Plk1 FRET sensor tagged to PACT domain at c-terminus, enabling centrosomal localisation^33^ (Addgene Plasmid #106907) using X-tremeGENE™ 9 (Merck: XTG9-RO) transfection reagent according to manufacturer’s instructions. Transfected cells were selected with DMEM with 10% FCS (Tet-System compatible) and 100 U/ml P/S supplemented with both 600 µg/ml of G418 (Invivogen: ant-gn-5) and 200 µg/ml Hygromycin-B (Invivogen: ant-hm-5), followed by single cell cloning.

RNAi-resistant constructs for Bora (Bora*) and Cep192 (Cep192*) were generated by introducing silent mutations within the siRNA target sequences using site-directed mutagenesis. Presence of the desired silent mutations and absence of unintended sequence changes was confirmed by Sanger sequencing, and these validated RNAi-resistant constructs were used to create stable cells expressing RNAi-resistant versions for rescue experiments. hTERT-RPE1 EGFP-Cep192* WT, hTERT-RPE1 EGFP-Cep192* 2A (T44A, S995A) cells were prepared by transfecting hTERT RPE1 cells with 0.5 µg plasmid containing EGFP-tagged Cep192 (WT or 2A) using X-tremeGENE™ 9 (Merck: XTG9-RO) transfection reagent according to manufacturer’s instructions. The transfected recombinants were first selected with 600 µg/ml of G418 (Invivogen: ant-gn-5) in DMEM with 10% FCS and 100 U/ml P/S and later cultured in the same medium to maintain EGFP-Cep192 expression.

hTERT-RPE1 EGFP–Bora* WT cells were generated by lentiviral transduction. Briefly, the coding sequence of RNAi-resistant Bora (WT) was cloned in-frame with an N-terminal EGFP tag into pTwist-Lenti-SFFV-Puro lentiviral expression vector. Lentiviral particles were produced in HEK293T packaging cells by co-transfection of the transfer plasmid together with psPAX2 and pMD2.G using standard calcium phosphate-based transfection as described below. Viral supernatant was collected 48–72 h post-transfection, filtered (0.45 µm), and used to transduce hTERT-RPE1 cells in the presence of polybrene (8 µg/ml). Following 24 h of infection, cells were selected using 5 µg/ml puromycin for 7 days to generate stable populations. Cell population expressing similar level of transgene was segregated from the whole by fluorescence-activated cell sorting (FACS) based on EGFP signal. The expression and localisation of EGFP–Bora was validated by fluorescence microscopy and immunoblotting. Similarly, hTERT-RPE1 PLK1-GFP + mScarlet3–PACT and hTERT-RPE1 Aurora A-mVenus + mScarlet3–PACT cell lines were generated by lentiviral transduction. Lentivirus was produced using a transfer plasmid (pTwist-Lenti-SFFV-Puro) encoding mScarlet3 fused to the PACT domain, following the protocol described above. Centrosomal localisation of the mScarlet3–PACT marker was verified by fluorescence microscopy. These dual-colour cell lines were used for live-cell imaging to quantify the temporal dynamics and centrosomal accumulation of PLK1 and Aurora A relative to centrosomes.

Centrosome devoid USP28^−/−^ cells were prepared by treating the cells with 300 nM of the Plk4 inhibitor centrinone^47^ followed by single-cell sorting by FACS and immunofluorescence-based selection of clones devoid of centrioles (Fig. S4). These cells were constantly maintained in 300 nM centrinone to prevent de-novo centriole formation.

For generating Plk1-EGFP knock-In cell line, hTERT-RPE1 cells were co-transfected with Cas9-eGFP, sgRNA 5′-TCGGCCAGCAACCGTCTCA-3′ (targeting Plk1) and a repair template (Genewiz). The repair template was designed as a fusion of 5xGly-eGFP flanked by two 500 bp arms, homologous to the genomic region around the Cas9 cutting site. Five days after transfection EGFP-positive cells were sorted and expanded for 1 week before a second sorting of single cells in a 96-well plates. After 2–3 weeks cells were screened by PCR.

### RNA interference

siRNA transfections were performed using Lipofectamine RNAiMAX (Thermo-Fisher Scientific: 13778075) according to manufacturer’s instructions. RNAi was performed for 72 h; when combined with inhibitors, the drugs were added 60 h post transfection. All the siRNA sequences used in the study were previously validated sequences: siControl (Qiagen, GGACCTGGAGGTCTGCTGT), siBora (Dharmacon, TAACTAGTCCTTCGCCTATTT)^76^, siCep192 (Dharmacon, AAGGAAGACATTTTCATCTCTTT), siCenexin (Dharmacon, GGCACAACATCGAGCGCAT), siCyclin A2 SMARTpool (Dharmacon, GGAAATGGAGGTTAAATGT, TAGCAGAGTTTGTGTACAT, ATGAGGATATTCACACATA, TGATAGATGCTGACCCATA), siAurora-A (Dharmacon, ATGCCCTGTCTTACTGTCA), siPlk1 (Dharmacon, CGAGCTGCTTAATGACGAG), and siSeparase (Dharmacon, GCTTGTGATGCCATCCTGA)^80^.

### Antibodies

The following antibodies were used in this study: mouse anti-α-tubulin (Geneva antibody facility: AA345-M2a; 1:250: ExM)^81^, mouse anti-β-tubulin (Geneva antibody facility: AA344-M2a; 1:250: ExM)^81^, mouse monoclonal anti-α-tubulin (Clone: DM1α, Sigma Aldrich, T9026, 1:5000: Western blotting), rabbit polyclonal anti-Bora (gift from Erich Nigg, 1:1000 Western blotting), rabbit polyclonal anti-CEP192 (Bethyl: A302-324A, 1:1000 Western blotting), rabbit polyclonal anti-Cenexin (abcam: ab43840, 1:1000 Western blotting), mouse monoclonal anti-Aurora A (Clone 4/IAK1, BD Biosciences: 610939, 1:1000 Western blotting), mouse monoclonal anti-Plk1 (Clone: 36-298, abcam: ab17057, 1:1000 Western blotting), rabbit polyclonal anti-pericentrin (abcam: ab4448; 1:250: ExM, 1:2000: IF), mouse monoclonal anti-Cyclin A2 (Clone: E32.1, abcam: ab38; 1:1000: Western Blotting), chicken polyclonal anti-GFP (Thermo-Fisher Scientific: A10262; 1:2000 IF), mouse monoclonal anti-centrin (Clone: 20H5, Merck Millipore: 04-1624; 1:1500 IF), rabbit polyclonal anti-53BP1 (Novus Biologicals: NB100-304: 1:2000 IF), mouse monoclonal anti-γ-tubulin (Clone: GTU-88, Sigma Aldrich: T6557, 1:2000 IF), mouse monoclonal anti-PCNA (Clone: PC10, Santacruz Biotechnology, SC-56), mouse monoclonal anti-γ-H2AX pSer139 (Clone: JBW301, Merck Millipore, 05-636), mouse anti-PLK1 (pT210) (BD Pharmingen™ Purified) (Clone: K50-483: BD Biosciences, 558400, 1:200 IF), mouse anti-GAPDH (Clone: GA1R, Thermo Fisher Scientific, MA5-15738: 1:2000 Western Blotting), rabbit polyclonal anti-GFP antibody (Origene: TP401, 1:5000 Western blotting) and mouse monoclonal anti-separase (abcam: ab16170; 1:500 Western blotting). All the Alexa Fluor-conjugated secondary antibodies were purchased from Thermo-Fisher Scientific and used at 1:500 dilution. For immunoblotting experiments, Horseradish peroxidase (HRP)-conjugated goat anti-mouse antibody was purchased from Thermo-Fisher (Cat # 32430), and HRP-conjugated goat anti-rabbit antibody was purchased from Bio-Rad (Cat # 1706515), and both of them were used at 1:10,000 dilution.

### FRET assay to measure Plk1 activity

Tetracycline inducible hTERT-RPE1 Cyclin A2-mScarlett Cells stably and constitutively expressing Plk1 FRET sensor were seeded in four-well glass bottom µ-Slide Ibidi chambers (Ibidi; 80426) and treated with different inhibitors/siRNAs as indicated in DMEM with 10% FCS and 1% P/S. The DMEM was replaced with Leibovitz L-15 supplemented with 10% FCS and 1% P/S, containing same inhibitors as before, if any. The chambers were acclimatised in 37 °C chamber before imaging. The acquisition was performed with an EC Plan Apochromat 100× (NA 1.56) oil objective on a Zeiss Cell Observer. Z1 spinning disk microscope (Nipkow Disk) equipped with a 37 °C chamber and a CSU X1 automatic Yokogawa spinning disk head. To perform FRET experiments, samples were illuminated with 1% laser intensity of 100 mW 445 nm laser, and the emission signal was split equally using DV2 split view system and CFP and YFP emissions were recorded on the split beams. 512 × 512-pixel size images were acquired with an evolve EM512 camera (photometrics) using Visiview 7.00.3 software. Acquired images were analysed using ImageJ to calculate the YFP to CFP emission intensity ratio after background subtraction. FRET analysis was performed in ImageJ. Background fluorescence was subtracted independently from both CFP and YFP channels using regions devoid of cells. To correct for spectral bleed-through, donor (CFP) and acceptor (YFP) crosstalk into the FRET channel were quantified using cells expressing donor-only or acceptor-only constructs, and corresponding correction coefficients were applied to all measurements. Corrected FRET was expressed as the CFP/YFP emission ratio (inverse FRET ratio). The regions of interest (ROIs) for cellular Plk1 activity were defined as circular areas of 5 µm diameter within the cyto-/nucleoplasm, excluding the nucleus and cell boundaries. For centrosomal measurements, circular ROIs of 2 µm diameter were centred on visually identifiable centrosomes and kept constant throughout the conditions. For determining the background, intensity values in each image were measured by transferring the same ROIs to the region devoid of cells. To facilitate interpretation, data are presented as inverse FRET ratios, which directly correlate with Plk1 activity. Sensor performance and dynamic range were validated by acute modulation of Plk1 activity using pharmacological inhibition, which resulted in the expected directional changes in FRET signal, confirming responsiveness within the experimental range.

### Immunofluorescence and quantitative image analysis

Cells treated with indicated inhibitors or siRNAs were fixed in prechilled methanol at −20 °C for 6 min. Fixed cells were blocked in Blocking solution (5% BSA in PBS), followed by incubating with indicated primary and secondary antibodies for 1 h and 30 min, respectively, with three washes of 10 min with PBS in between. Stained coverslips were mounted on glass slides with Vectashield with DAPI (Vector Laboratories: H-1200-10) and visualised using a 100× PLAN Apochromat oil-immersion (NA 1.4) objective on an Olympus DeltaVision microscope (GE Healthcare) equipped with a DAPI/FITC/Rhodamine/CY5 filter set (Chroma Technology Corp) and a CoolSNAP HQ camera (Roper-Scientific). The three- dimensional image stacks were deconvolved with SoftWorx (GE Healthcare). The acquired images were cropped and processed with ImageJ (NIH) software to generate intensity projections (maximum/average) and subjected to background subtraction (unless specified) before extracting the fluorescence intensity and pixel size values according to the analysis as described below.

Briefly, hTERT-RPE-1 expressing endogenously GFP-tagged Plk1 were used for all analyses. Cells were co-stained with PCNA or pericentrin to identify S-phase and G2-phase cells, respectively. S-phase cells were defined by the presence of characteristic bright nuclear PCNA foci, whereas G2-phase cells were identified by the presence of two spatially separated pericentrin-positive centrosomes within cells exhibiting an intact nuclear envelope. Fluorescence intensities of phosphorylated Plk1 (pT210) and total Plk1 were quantified from maximum-intensity projections. For each cell, the ratio of pT210-Plk1 to total Plk1 was calculated. Individual values were normalized to the mean of the control condition, and the full distribution of normalized values was plotted. For measuring centrosomal Plk1 levels after RNAi of Plk1 activators in G2, pericentrin signals were used to generate centrosomal masks, which were subsequently applied to the PLK1–EGFP channel to extract centrosome-associated fluorescence intensities. Values were normalized to the mean centrosomal PLK1 intensity in control cells prior to plotting.

Centrosome maturation was assessed by quantifying the 3D volumes of pericentrin and γ-tubulin from z-stack images using ImageJ. The volume of CEP192 was quantified using the same approach. The number of PCNA foci per nucleus was quantified by particle analysis in ImageJ. Only particles with an apparent diameter ≥300 nm and having minimum three times more intensity values as compared to background (SNR ≥ 3) were included in the analysis. DNA damage was quantified by measuring the size distribution of nuclear γ-H2AX and 53BP1 foci. Particle diameters were extracted using the ImageJ particle analysis plugin, and the full distribution of foci sizes was plotted.

Centrosomal recruitment of Plk1 and Aurora A was assessed by line-scan intensity profiling in cells expressing endogenously tagged PLK1–GFP or Aurora A–mVenus. Cells were synchronised using a CDK4/6 inhibitor, released, and fixed at the indicated time points, followed by immunostaining for PLK1 or Aurora A. Centrosomes were identified using pericentrin. Images were acquired at each time point, and a 5 µm line with a width of 5 pixels was drawn across individual centrosomes, positioning the centrosome at the centre of the line. Fluorescence intensity profiles were extracted for each channel, averaged across the line width and normalised to the cellular background for each channel. Co-localization was defined by the presence of coincident intensity peaks of PLK1 or Aurora A with the centrosomal marker at the same spatial position. A minimum of 11 centrosomes were analysed at each time point.

### Immunoblotting

Cells were grown in 60 mm plastic dishes and treated with inhibitors/drugs overnight. To prepare protein lysate, the cells were scrapped off using cell scrapper and lysed in RIPA buffer (50 mM Tris pH-7.4, 150 mM NaCl, 1% Nonidet P-40 (Thermo-Fisher Scientific: 85124), 0.5% sodium deoxycholate (Sigma Aldrich: D5670), 0.1% sodium dodecyl-sulfate in ultrapure water) supplemented with protease inhibitor (Roche: 11873580001) and phospho-STOP (Roche: 04906845001). Protein concentrations in the lysates were determined using the Bradford Protein Assay (Thermo-Fisher; 23200). Samples with equal amounts of protein were mixed with 4× Laemmli buffer and heated to 95 °C for 5 min. Proteins were separated on a 10% SDS-polyacrylamide gels and transferred onto a 0.45 µm pore size nitrocellulose membrane (Macherey-Nagel GMBH: 741280) by wet blotting. Membranes were blocked with 5% non-fat dry milk in TBS 0.1% Tween20 (TBS-T) for 30 min. After blocking, membranes were incubated with primary antibodies overnight at 4 °C in TBS-T 5% non-fat dry milk. Membranes were washed three times with TBS-T and incubated 1 h with the appropriate peroxidase-conjugated secondary antibody in TBS-T 5% non-fat dry milk. The membranes were washed thrice with TBS-T, and the bands corresponding to the protein of interest were detected by chemiluminescence using the Amersham ECL Prime Western Blotting Detection Kit (GE Healthcare; RPN2232) in a Fusion FX7 Spectra Multispectral Imaging system (Witec AG, Switzerland).

### Live cell imaging and analysis

For live cell imaging experiments, hTERT-RPE1 cells endogenously tagged with mRuby-PCNA were plated in glass-bottom Ibidi chambers (Ibidi GMBH: Cat # 81158), and normal DMEM medium was replaced with L15 Leibovitz’s medium supplemented with 10% FCS, 100 U/ml P/S, 50 nM SiR-tubulin, and 100 nM SPY505-DNA prior to imaging. The cells were treated with indicated inhibitors/siRNAs and imaged at 37 °C on a Nikon Ti microscope equipped with a 60× NA 1.3 oil-immersion objective, DAPI/FITC/rhodamine/CY5 (Chroma, USA) filter set, Orca Flash 4.0 CMOS camera (Hamamatsu, Japan), and the NIS software. Cells were recorded every 10 min for 48 h with z-slices separated by 1 μm, and 100 ms exposure per z-slice at wavelengths of 488 (525), 561 nm (615 nm), and 647 (670) excitations (emission). The time-lapse movies were analysed manually for determining the duration of S and G2 phases based on PCNA localisation (Fig. 3A) using Imaris software (Bitplane Inc). Briefly, PCNA in the nucleus is diffused and low during G1, becomes punctate, marking active replication forks throughout S-phase, and returns to a more uniform but brighter nuclear distribution in G2. During mitosis, PCNA dissociates from chromatin and appears diffuse or largely excluded from condensed chromosomes. These criteria were used to identify the G1/S, S/G2, and G2/M transitions in the live-cell recordings.

### FRAP analysis determining mobile Plk1 fraction on centrosomes

hTERT-RPE1 cells expressing Plk1 tagged with GFP at endogenous locus were plated in glass bottom Ibidi chambers (Ibidi GMBH: Cat # 81158) in DMEM media and treated with indicated siRNAs or drugs. Prior to imaging, the normal DMEM medium was replaced with L15 Leibovitz’s medium supplemented with 10% FCS. Presence of 2 separate centrosomal foci was used as the marker to identify G2 phase cells for imaging. FRAP experiments were performed on a Nikon A1R point scanning confocal microscope (Nikon Europe B.V.) using 60 × 1.40 NA Plan Apochromat objective using a 50 mW 488-nm laser equipped with 37 °C heating chamber and PMT and GaAsP detectors. A small square ROI 2 µm × 2 µm was created around the centrosomes and was then photobleached using 100% laser power. Both pre-bleach and post-bleach images were acquired at 1% laser power to minimise photobleaching during imaging. A second ROI was also created outside the cell and subjected to identical analysis to calculate the background. Fluorescence recovery was monitored by acquiring images post-bleaching at every 5 s for a total duration of 3 min. Fluorescence intensities were quantified using NIS software after background subtraction and normalisation to pre-bleach intensity values. From these relative fluorescence intensities obtained were used to generate recovery curves from multiple cells and fitted using standard exponential recovery models. The mobile fraction of Plk1 was calculated for each case using the formula below, with each value derived from the recovery curve.

$${{{\rm{mobile\; fraction}}}}=({F}_{\infty }-{F}_{0})/({F}_{{pre}}-{F}_{0})$$

where, *F*_pre_, *F*_0_ and *F*_∞_ refer to the relative fluorescence intensity before photobleaching, relative fluorescence intensity immediately after photobleaching, and relative fluorescence intensity at the plateau, respectively.

### Flow cytometry-based cell cycle profile analysis

Asynchronously growing cells in log phase were collected by trypsinisation in a microcentrifuge tube, counted, and fixed with prechilled 70% ethanol and incubated overnight at −20 °C. Next day, the cells were washed with 1× PBS and resuspended in PI/RNase Staining Buffer (BD Biosciences: 550825) to a final concentration of 0.5 million cells per ml of suspension and incubated in dark for 1 h at room temperature. The DNA content in the cells was analysed on Cytoflex flow cytometer (Beckman Coulter) using 561 nm laser after removing the doublets. The acquired data was analysed in FlowJo 10 Software (BD Biosciences) to extract the percentage of cells in different cell cycle stages based on DNA content.

### Expansion microscopy

Cells were grown on 12 mm circular glass coverslips (Thermo-Fisher Scientific) and treated with required inhibitors/drugs overnight. Next day the coverslips were treated with acrylamide (AA)-formaldehyde (FA) solution [1.4% AA (Sigma Aldrich: A4058) and 2% FA (Sigma Aldrich: F8775) in PBS] for 5 h at 37 °C to prevent protein crosslinking. Coverslips were next subjected to gelation by incubation for 1 h at 37 °C with monomer solution [19% sodium acrylate (Sigma Aldrich: 408220), 10% acrylamide, 0.1% bis-acrylamide (Sigma Aldrich: M1533), 0.5% tetramethyl ethylenediamine-TEMED (Thermo Fisher: 17919), 0.5% ammonium persulfate (Thermo Fisher: 17874) in PBS]. Post gelation, the coverslips were treated with denaturation solution [50 mM Tris (Sigma Aldrich: 99362), 200 mM sodium dodecyl sulfate (Axon Lab AG: A2572.0500), 200 mM sodium chloride (Axon Lab AG: A3597.1000) in nuclease free water, pH: 9.0] for 15 min on a rocker shaker at room temperature to detach the gels from coverslips. The gels were heated at 95 °C for 90 min in the denaturation solution, followed by three 30 min washes with water. The gels were incubated with PBS for 15 min followed by 3 h incubations with primary and secondary antibodies, followed each by three 10 min washes at 37 °C and gentle shaking. Stained gels were kept overnight in water for optimal expansion. The size of gel was measured to calculate the expansion factor, and the gel was cut into small pieces and placed in 2 well plastic bottom Ibidi chamber (Ibidi GMBH: Cat # 80286). The 3D image stacks of centrioles in G2 phase cells (4 centrioles in one cell) were acquired in 0.1 µm steps using a 100× PLAN-Apochromat oil-immersion (NA 1.4) objective on an Olympus DeltaVision microscope (GE Healthcare) equipped with a DAPI/FITC/rhodamine/CY5 filter set (Chroma Technology Corp) and a CoolSNAP HQ camera (Roper-Scientific). The three- dimensional image stacks were deconvolved with SoftWorx (GE Healthcare). The acquired images were cropped and processed with ImageJ (NIH) software to construct 3D image to analyse the configuration of centrioles (orthogonal orientation and distance in between) for each image.

### Separase activity measurements

Separase activity measurements were performed on live cells. hTERT-RPE1 cells expressing separase sensor were plated in glass bottom Ibidi chambers (Ibidi GMBH: Cat # 81158), and DMEM containing Tet-System approved FCS and transfected with indicated siRNAs. DMEM was replaced with L15 Leibovitz’s medium supplemented with 10% FCS, 100 U/ml P/S, 50 nM SiR-DNA, and 1 µg/ml doxycycline 3 h prior to imaging. Cells with two distinct centrosomes and intact nucleus (G2 phase) were selected, and 3D image stacks of centrosomes were acquired for FITC, TRITC, and Cy5 channels in 0.1 µm incremental z-steps using a 100× oil-immersion (NA 1.4) objective on an Olympus DeltaVision microscope (GE Healthcare) equipped with a DAPI/FITC/rhodamine/CY5 filter set (Chroma Technology Corp) and a CoolSNAP HQ camera (Roper-Scientific). The three-dimensional image stacks were deconvolved with SoftWorx (GE Healthcare). The acquired images were cropped and processed with ImageJ (NIH) software to extract the intensities of GFP and mCherry in the centrosomes for each image and plotted as the ratio of GFP to mCherry as the measure of separase activity.

### Measuring the relative Plk1 levels on centrosomes

Determining of relative Plk1 concentration on the centrosomes w.r.t cytoplasm was performed by comparing the total Plk1 present on purified centrosomes and total cell lysate prepared from equivalent number of cells. Centrosomes were isolated from human lymphoblastic KE37 cells using a sucrose density gradient centrifugation approach as previously described^37,82^. Briefly, KE37 cells were grown to a density of approx. 1–2 × 10^6^ cells/ml and treated with 330 nM nocodazole (Sigma Aldrich: M1404-2MG), 1 µg/ml Cytochalasin D (Sigma Aldrich: C8273) for 1 h at 37 °C to depolymerise microtubules and actin, thereby facilitating centrosome release. All subsequent steps were performed at 4 °C unless otherwise stated. Cells were collected by centrifugation followed by hypotonic lysis (1 mM HEPES, 0.5% NP-40, 0.5 mM MgCl_2_, supplemented with protease inhibitors), which allows chromatin dispersion while maintaining centrosome integrity. Centrosomes are harvested by centrifugation in onto a 50% sucrose cushion and further purified by centrifugation through a discontinuous (70%, 50%, and 40%) sucrose gradient in PIPES-based buffer (10 mM K.PIPES, pH 7.2, 0.1% Triton X-100 and 0.1% ß-mercaptoethanol) at 110,000 × *g* at 4 °C for 75 min using SW-41 Ti rotor in Beckman Coulter Optima XPN-100 ultracentrifuge (Beckman Coulter, Inc). Gradients were fractionated by collecting fractions of 0.5 ml from the bottom, and centrosome-containing fractions (typically within the 40–70% sucrose range) were analysed by immunofluorescence for determining the centrosome concentration. Fractions enriched in centrosomes were pooled, aliquoted, snap-frozen in liquid nitrogen, and stored at −80 °C for measuring Plk1 levels. From the centrosome-containing fractions, a volume corresponding to 250,000 centrosomes was mixed with Laemmli Buffer and loaded on to 10 % SDS-PAGE along with 1% of the total cell lysate prepared from the same number of cells. The two samples running side by side were probed for Plk1 (to compare levels) and GAPDH (estimate the purity of isolated centrosomes).

### Statistical analysis

Statistical tests for all figures were performed using GraphPad Prism 10 (GraphPad); the statistical tests employed in every case are described in the figure legends. Minimum three independent biological replicates were performed in all experiments.

### Reporting summary

Further information on research design is available in the Nature Portfolio Reporting Summary linked to this article.

## Supplementary information

Supplementary Information

Description of Additional Supplementary Files

Movie 1

Movie 2

Movie 3

Movie 4

Movie 5

Movie 6

Movie 7

Movie 8

Movie 9

Movie 10

Movie 11

Movie 12

Movie 13

Movie 14

Movie 15

Movie 16

Movie 17

Movie 18

Movie 19

Movie 20

Movie 21

Movie 22

Movie 23

Movie 24

Movie 25

Movie 26

Movie 27

Movie 28

Movie 29

Movie 30

Movie 31

Movie 32

Movie 33

Movie 34

Movie 35

Reporting Summary

Transparent Peer Review File

## Source data

Source Data

## Data Availability

All the raw data related to figures and Supplementary Figs. (representative images and movies) are available at: 10.26037/yareta:7rmgsonvwne33i42slk3uf2yfi. Due to the large size (>4 TB), the raw live-cell imaging data used for analyses will be made available on request to the corresponding authors by sending external hard disks. Source data are provided with this paper.
