# Supplementary material for: Bora, CEP192 and Cenexin regulate distinct Plk1-dependent cell and centrosome cycle transitions
Source: Nat Commun. 2026 Jun 30;17:8158. doi: 10.1038/s41467-026-75033-5 (PMC13458439; doi:10.1038/s41467-026-75033-5)
Supplement: Supplementary file 1 [file 41467_2026_75033_MOESM1_ESM.pdf]

Supplementary Information

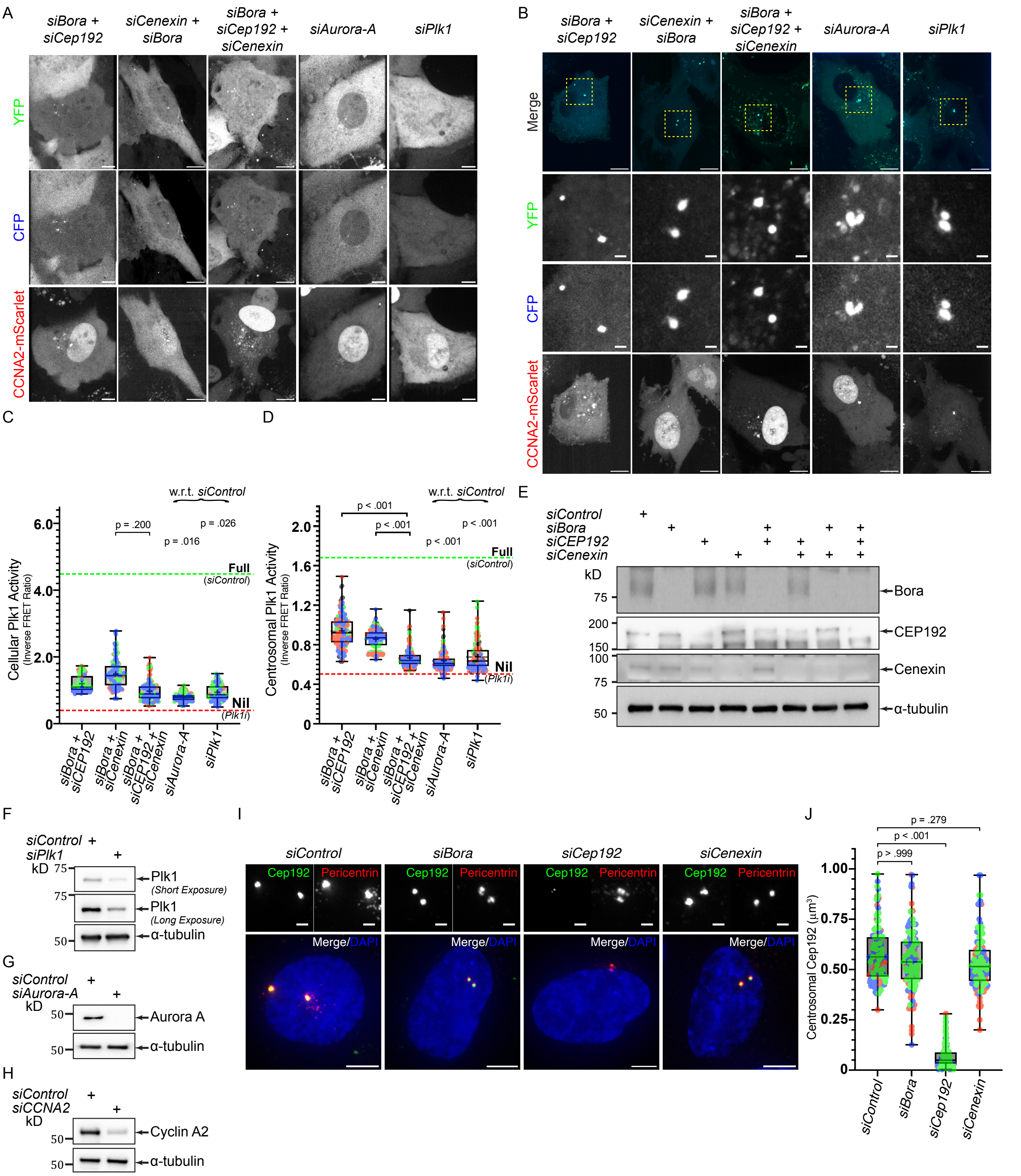

Figure S1: Different Plk1 activators activate distinct Plk1 pools. (A-B) Representative images of G2 phase hTERT-RPE1 cells expressing cellular Plk1 sensor **(A)** or centrosomal Plk1 sensor **(B)** together with CCNA2-mScarlet and treated as indicated. **(C-D)** Quantification for cellular **(C)** and centrosomal **(D)** Plk1 activity from G2 cells in **(A)** and **(C)**, respectively; N=3, n= 120: siBora + siCep192, 135: siBora + siCenexin, 127: siBora + siCep192 + siCenexin, 124: siAurora and 121: siPlk1 cells (Whole cells) and N=4, n= 181: siBora + siCep192, 187: siBora + siCenexin, 181: siBora + siCep192 + siCenexin, 182: siAurora and 182: siPlk1 cells (centrosomal). **(E-H)** Western Blot confirming the depletion of indicated proteins after siRNA treatments. Note that in a first approximation, depletion of one Plk1 activator did not change the expression of the other activators; our data does however not exclude more minor changes due to changes in cell cycle phase durations. **(I)** Representative images of G2 phase hTERT RPE1 cells stained with Cep192 and Pericentrin and treated with indicated siRNAs. **(J)** Quantification for centrosomal levels of Cep192 in conditions depicted in **(I)**; N=3, n=130: siControl, 138: siBora, 142: siCep192 and 132: siCenexin cells. p-values: Kruskal-Wallis test (two-sided) **(C, D and J)** are indicated. The boxes indicate 1st quartile (minima), median (centre) and 3rd quartiles (maxima) and whiskers represent minimum and maximum values. Overlaid dots represent individual observations and '+' sign represent mean value. Dots of same colour represent cells derived from same experiment. Statistical comparisons not discussed in main text are omitted for clarity and are provided in Supplementary Data. Scale bars, Images: 5 μm and zoomed insets: 0.5 μm.

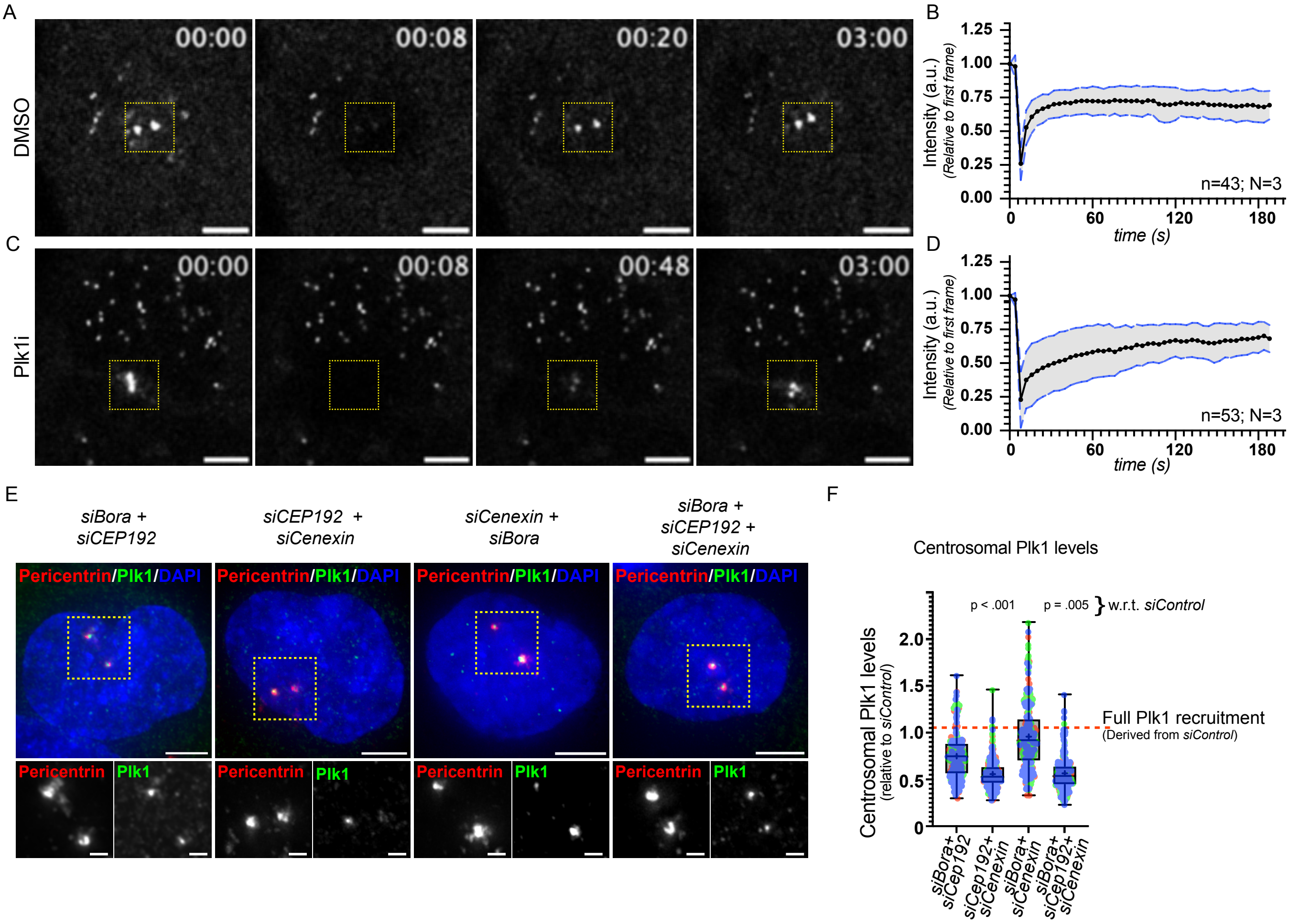

Figure S2: Plk1 activity does not affect the resident pool of Plk1 on centrosomes. (A-D) Representative time stamp images and quantifications from FRAP movies from G2 phase Plk1-EGFP tagged hTERT-RPE1 cells treated with DMSO (**A**: quantification of Plk1 recovery on centrosomes in **(B)**) and Plk1i ((**C**: quantification of Plk1 recovery on centrosomes in **(D)**); N=3, n=43 and 53 DMSO and Plk1i treated cells, respectively. **(E)** Representative immunofluorescence images of hTERT-RPE1: Plk1-EGFP cells treated with indicated siRNAs and stained for Pericentrin (Red) and EGFP (Green) for Plk1. Data represented as mean ± SD. **(F)** Quantification for Plk1 levels on centrosomes during G2 phase under the conditions indicated in **(E)**; N=5, n= 276: siBora + siCep192, 287: siCep192 + siCenexin, 286: Bora + siCenexin and 282: siBora + siCep192 + siCenexin cells. p-values: Kruskal-Wallis test (two-sided) **(F)** are indicated. The boxes indicate 1st quartile (minima), median (centre) and 3rd quartiles (maxima) and whiskers represent minimum and maximum values. Overlaid dots represent individual observations and '+' sign represent mean value. Dots of same colour represent cells derived from same experiment. Statistical comparisons not discussed in main text are omitted for clarity and are provided in Supplementary Data. Scale bars, Images: 5 μm and zoomed insets: 0.5 μm.

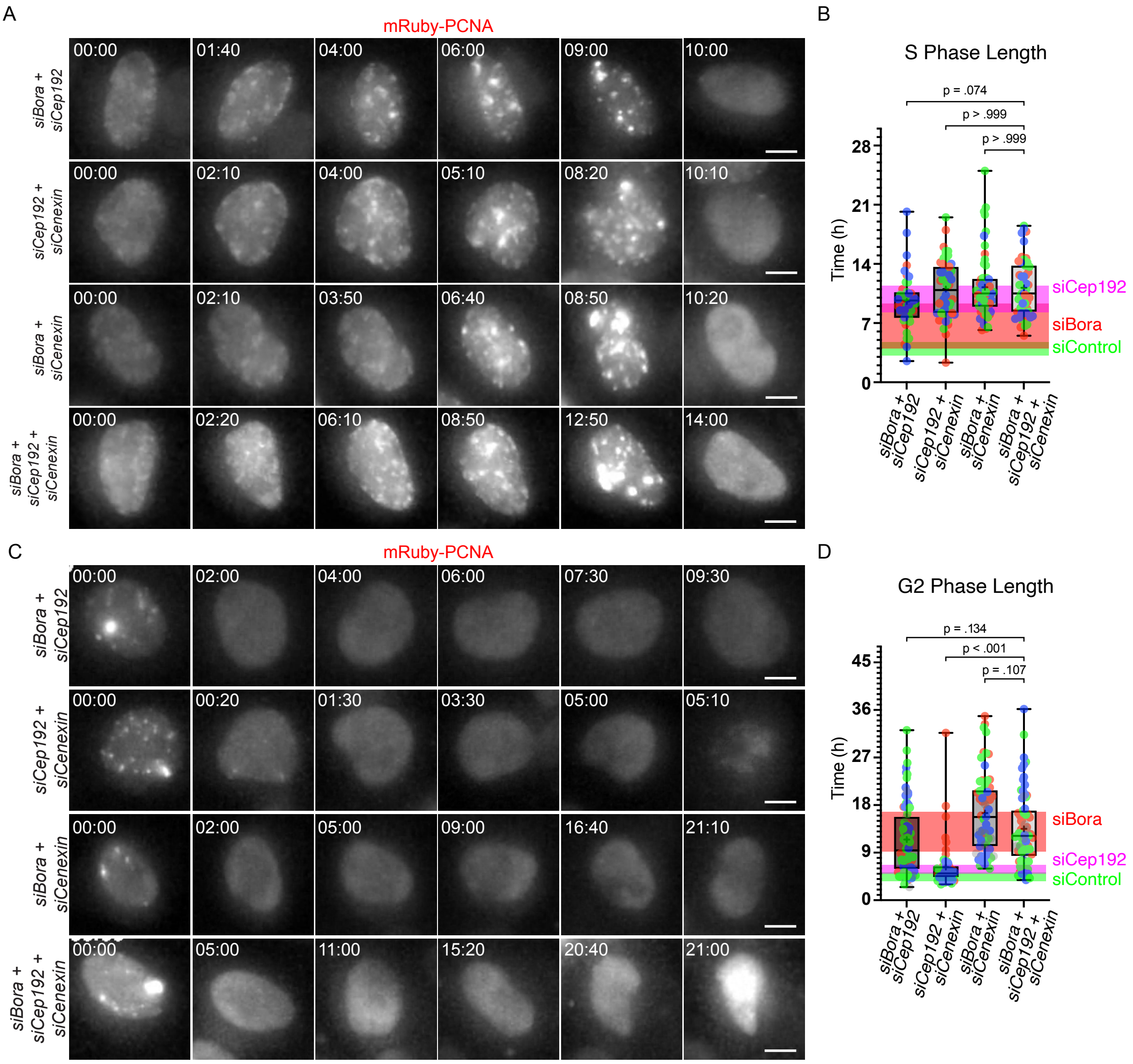

Figure S3: Perturbing Plk1 activity delays the progression through S and G2 phase. (A) Live-cell time-lapse images of hTERT-RPE1: mRuby-PCNA cells treated with indicated siRNA combinations as they progress through S phase. **(B)** Quantification for the duration of S phase in cells after depletion of indicated proteins in **(A)**; N=3, n= 45: siBora + Cep192, 52: siCep192 + siCenexin, 57: siBora + siCenexin and 51: siBora + siCep192 + siCenexin cells. **(C)** Live-cell time-lapse images of hTERT-RPE1: mRuby-PCNA cells treated with indicated siRNA combinations as they progress through G2 phase. **(D)** Quantification for the duration of G2 phase in cells after depletion of indicated proteins in **(C)**; N=3, n= 82: siBora + Cep192, 56: siCep192 + siCenexin, 64: siBora + siCenexin and 71: siBora + siCep192 + siCenexin cells. p-values: Kruskal-Wallis test (two-sided) (**B** and **D**) are indicated. The boxes indicate 1st quartile (minima), median (centre) and 3rd quartiles (maxima) and whiskers represent minimum and maximum values. Overlaid dots represent individual observations and '+' sign represent mean value. Dots of same colour represent cells derived from same experiment. Statistical comparisons not discussed in main text are omitted for clarity and are provided in Supplementary Data. Scale bars: 5 μm.

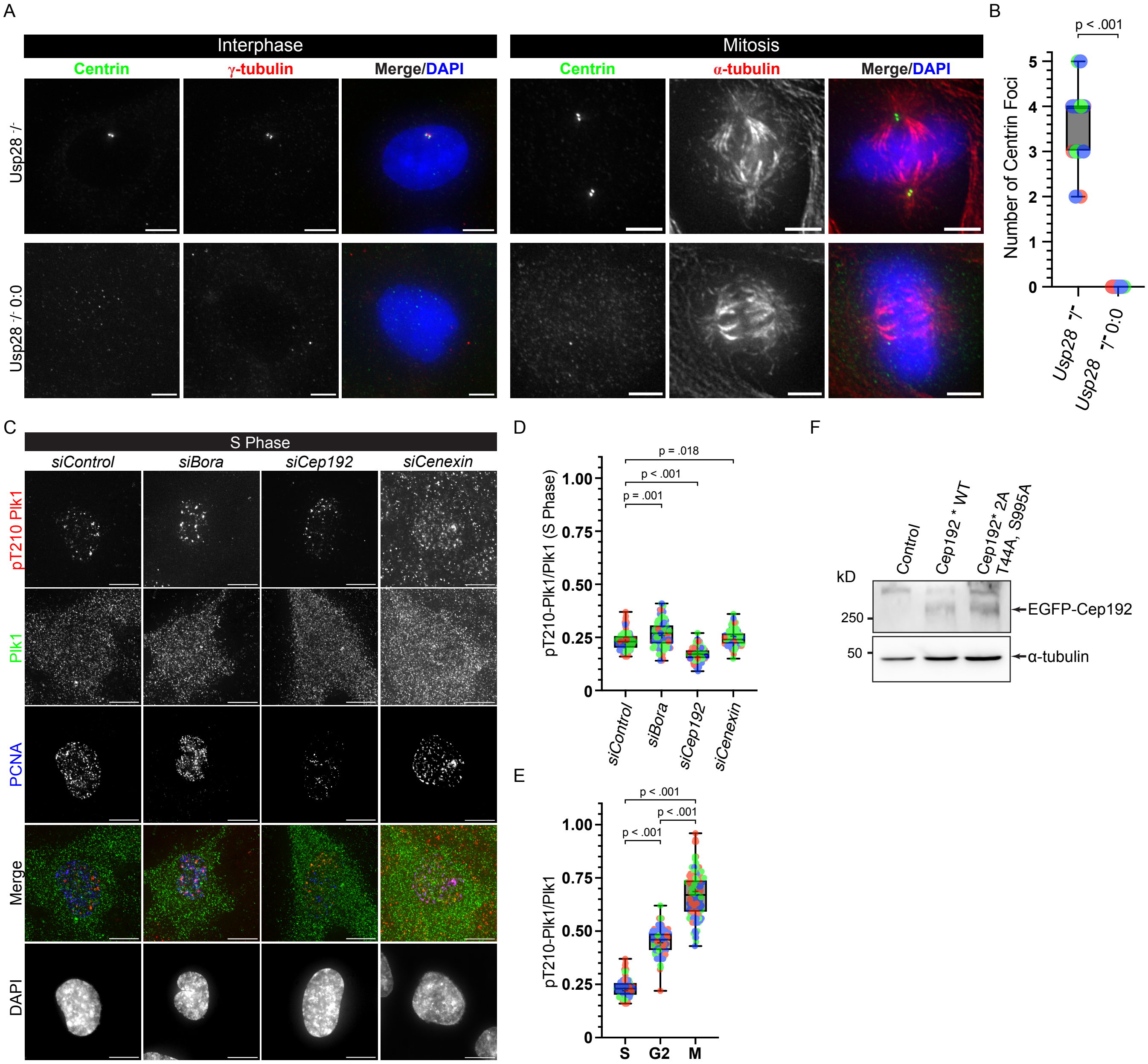

Figure S4: Acentrosomal cells lack centrioles and Cep192 depletion abrogates Plk1 activity in S phase. (A) Representative images of interphase and metaphase hTERT-RPE1: Usp28 -/- and hTERT-RPE1: Usp28 -/- 0:0 cells stained for centrioles with Centrin (green) and γ-tubulin (interphase cells)/α-tubulin (mitotic cells) (red) confirming absence of centrosomes. **(B)** Quantification for the number of centrioles present in hTERT-RPE1: Usp28 -/- and hTERT-RPE1: Usp28 -/- 0:0 cells; N=3, n= 47 cells in each case. **(C)** Representative immunofluorescence images of S phase hTERT-RPE1 Plk1-EGFP cells treated as indicated and stained for pT210-Plk1, Plk1-GFP (anti-GFP) and PCNA. **(D)** Quantification for pPlk1/total-Plk1 in cells in **(C);** N=3, n= 104; siControl, 108: siBora, 101: siCep192 and 105: siCenexin cells). **(E)** Quantification for pPlk1/total-Plk1 in S, G2 and M phase cells represented in **(C)** and **(Fig 1G)**; N=3, n= 104; S, 104: G2, and 116: Mitotic cells). **(F)** Western Blot confirming the expression of Cep192 (WT or 2A mutant) in stable cells. p-values: two-tailed Mann-Whitney test **(B)** and Kruskal-Wallis test (two-sided) **(D and E)** are indicated. The boxes indicate 1st quartile (minima), median (centre) and 3rd quartiles (maxima) and whiskers represent minimum and maximum values. Overlaid dots represent individual observations and '+' sign represent mean value. Dots of same colour represent cells derived from same experiment. Scale bars: 5 μm.

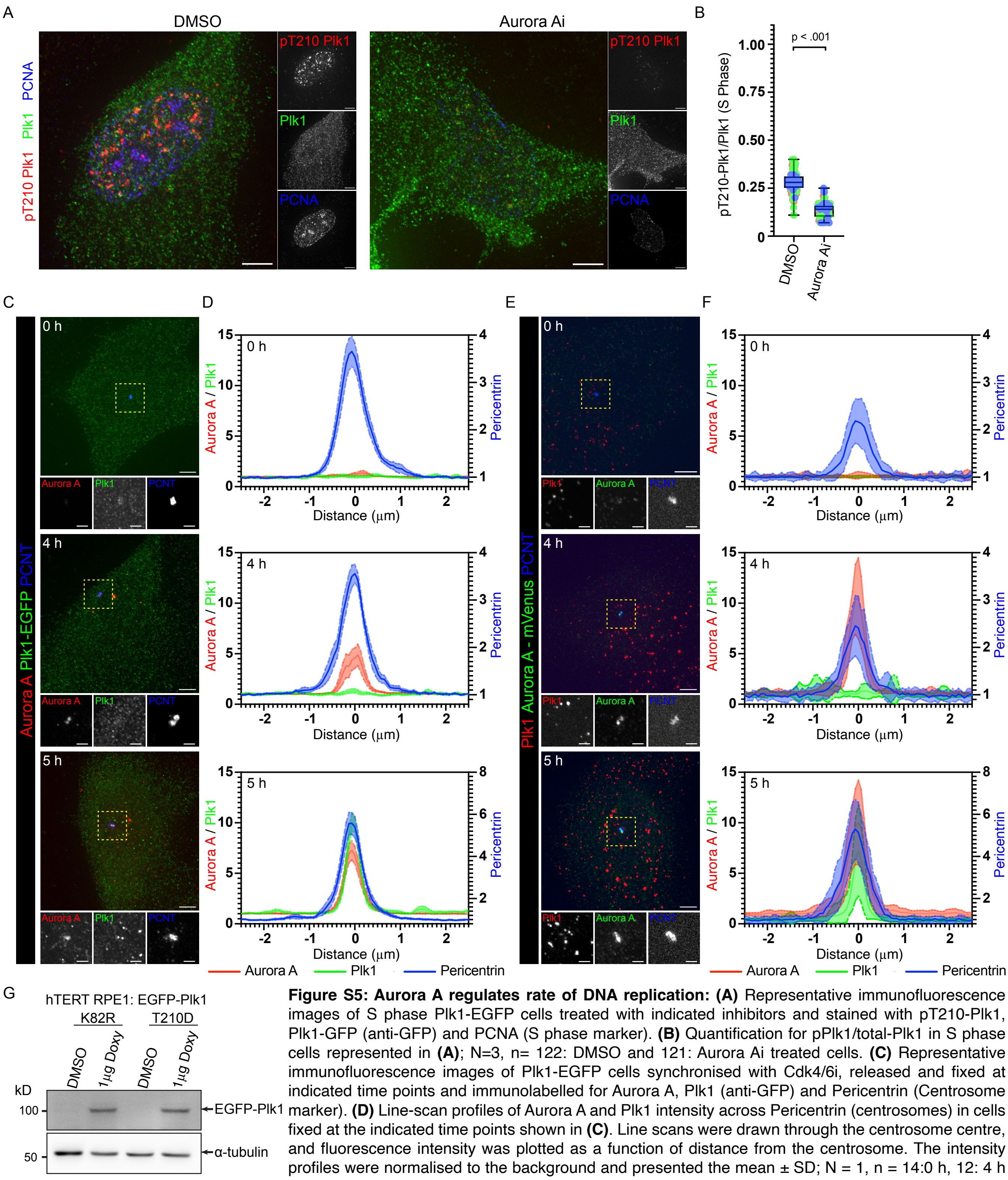

Figure S5: Aurora A regulates rate of DNA replication: (A) Representative immunofluorescence images of S phase Plk1-EGFP cells treated with indicated inhibitors and stained with pT210-Plk1, Plk1-GFP (anti-GFP) and PCNA (S phase marker). **(B)** Quantification for pPlk1/total-Plk1 in S phase cells represented in **(A)**; N=3, n= 122: DMSO and 121: Aurora Ai treated cells. **(C)** Representative immunofluorescence images of Plk1-EGFP cells synchronised with Cdk4/6i, released and fixed at indicated time points and immunolabelled for Aurora A, Plk1 (anti-GFP) and Pericentrin (Centrosome marker). **(D)** Line-scan profiles of Aurora A and Plk1 intensity across Pericentrin (centrosomes) in cells fixed at the indicated time points shown in **(C)**. Line scans were drawn through the centrosome centre, and fluorescence intensity was plotted as a function of distance from the centrosome. The intensity profiles were normalised to the background and presented the mean ± SD; N = 1, n = 14:0 h, 12: 4 h and 14: 5 h cells. **(E)** Representative immunofluorescence images of Aurora A-mVenus cells synchronised with Cdk4/6i, released and fixed at indicated time and immunolabelled for Plk1, Aurora A (anti-GFP) and Pericentrin (Centrosome marker). **(F)** Line-scan profiles of Aurora A and Plk1 intensity across Pericentrin in cells fixed at the indicated time points shown in **(E)**. Line scans were drawn in the same manner as in **(D)**. The intensity profiles were normalised to the background and presented the mean of n = 11:0 h, 11: 4 h and 13: 5 h cells. Data plotted as mean ± SD. **(G)** Immunoblot confirmation of expression of Plk1 mutants after Doxycycline induction. p-values: two-tailed Mann-Whitney test **(B)** are indicated. The boxes indicate 1st quartile (minima), median (centre) and 3rd quartiles (maxima) and whiskers represent minimum and maximum values. Overlaid dots represent individual observations and '+' sign represent mean value. Dots of same colour represent cells derived from same experiment. Statistical comparisons not discussed in main text are omitted for clarity and are provided in the Supplementary Data. Scale bars for whole cells = 5 μm and zoomed insets = 0.5 μm.

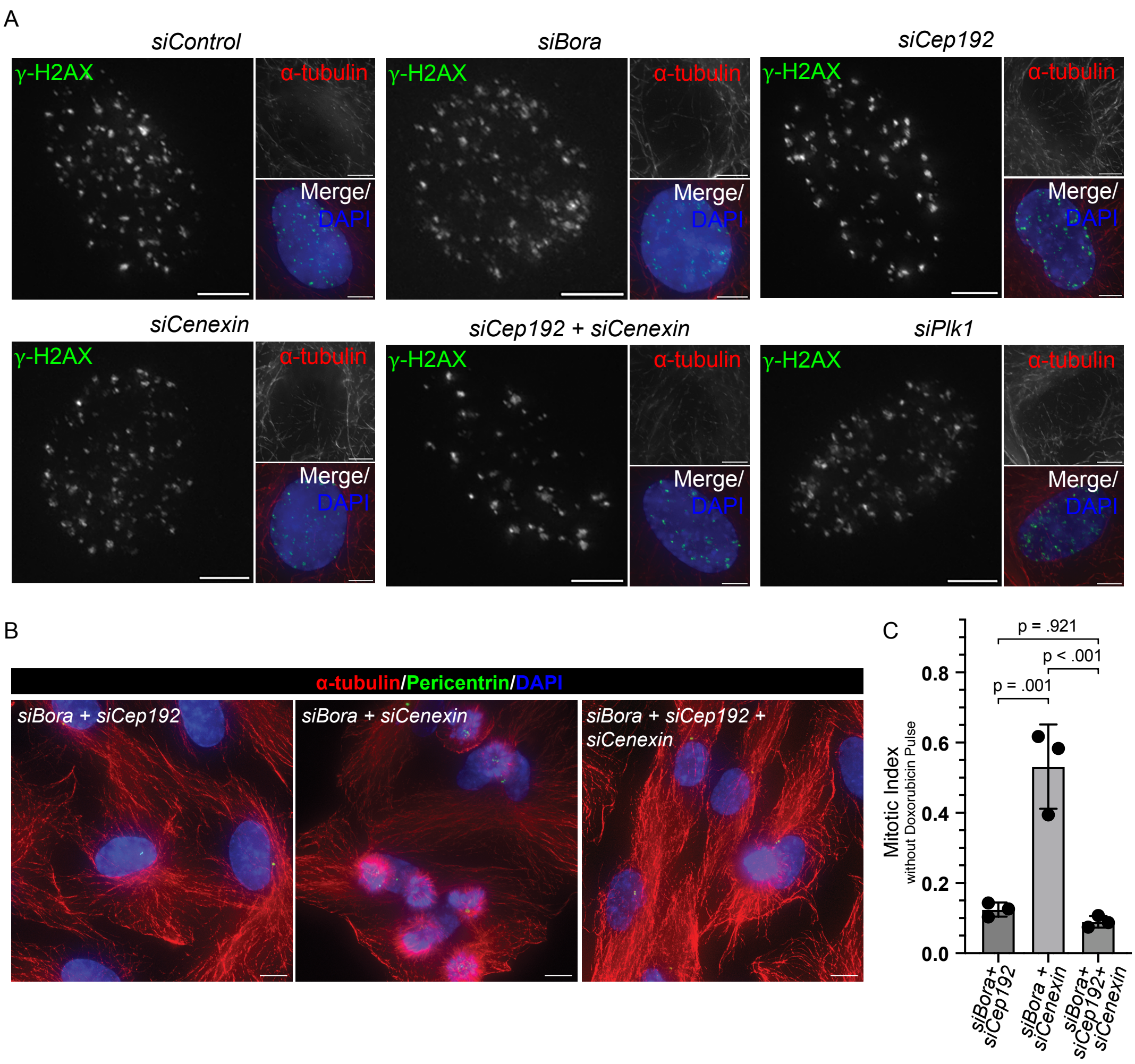

Figure S6: Confirmation of DNA damage induction by Doxorubicin pulse. (A) Representative images of synchronised and doxorubicin pulsed hTERT-RPE1 cells treated with indicated siRNA combinations and stained for γ-H2AX (green) to confirm induction of DNA damage after doxorubicin pulse. **(B)** Representative field images of hTERT-RPE1 cells treated with indicated siRNAs and stained for α-tubulin, Pericentrin and DAPI. **(C)** Quantification representing mitotic index in cells without doxorubicin pulse under indicated depletion conditions shown in **(B);** N=3, n=591: siBora + siCep192, 955: siBora + siCenexin and 637: siBora + siCep192 + siCenexin cells. The overlaid dots in each plot represent individual observations. The p-values: one-way ANOVA is indicated. '+' sign in each column represent the mean value. Statistical comparisons not discussed in the main text are omitted for clarity; complete results are provided in the Supplementary Data. Scale bars: 5 μm.

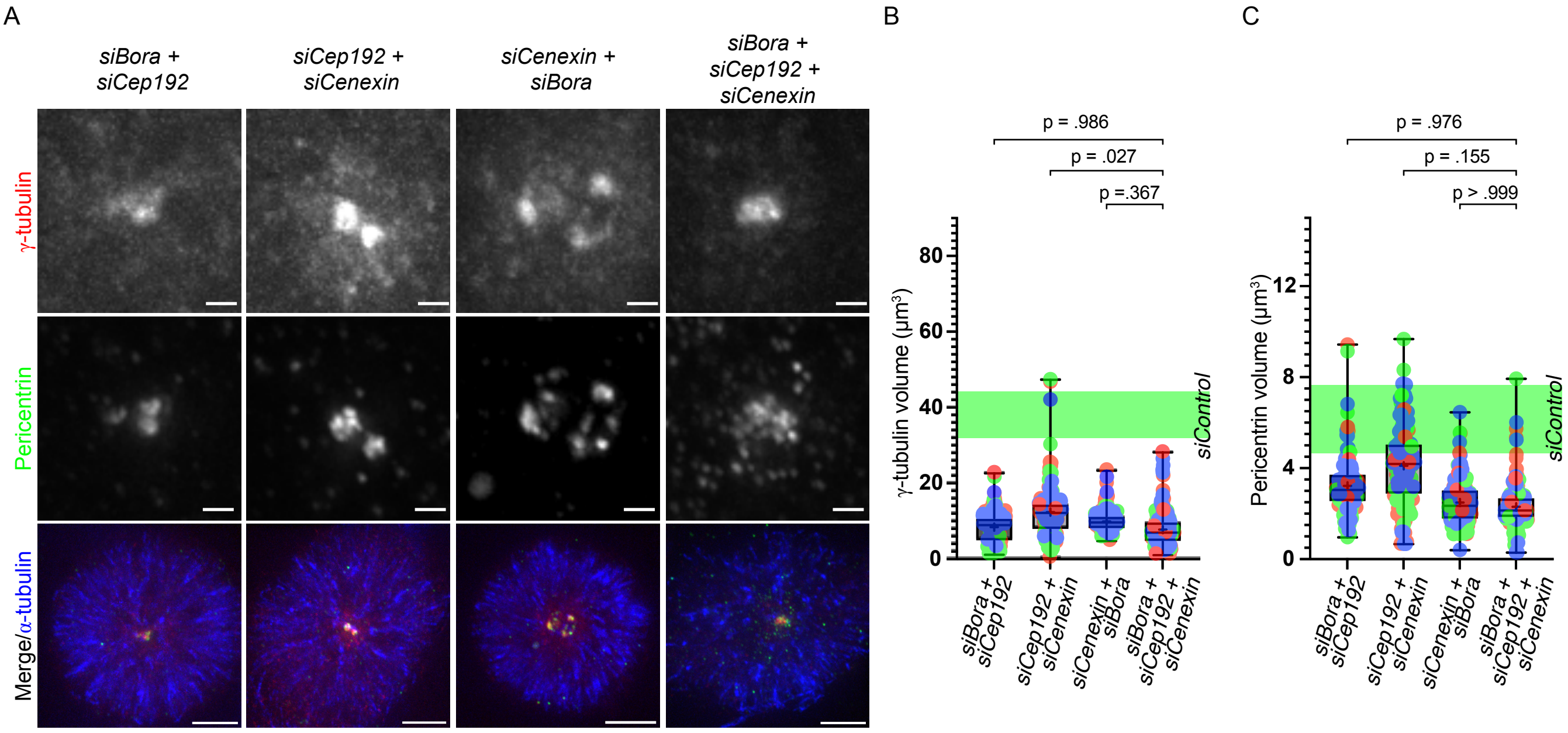

Figure S7: Plk1 activity is required for centriole maturation. (A) Representative images of G2 phase centrosomes stained with γ-tubulin (red) and Pericentrin (green) in hTERT-RPE1 cells treated with indicated siRNA combinations. **(B-C)** Quantifications for centrosomal levels of γ-tubulin **(B)** and Pericentrin **(C)** in cells represented in (A); N=3, n= (138: siBora + siCep192, 164: siCep192 + siCenexin, 181: siCenexin + siBora and 167: siBora + siCep192 + siCenexin for γ-tubulin) and (n= 136: siBora + siCep192, 167: siCep192 + siCenexin, 181: siCenexin + siBora and 170: siBora + siCep192 + siCenexin for Pericentrin (green) cells. p-values: Kruskal-Wallis test (two-sided) (B and C) are indicated. The boxes indicate 1st quartile (minima), median (centre) and 3rd quartiles (maxima) and whiskers represent minimum and maximum values. Overlaid dots represent individual observations and '+' sign represent mean value. Dots of same colour represent cells derived from same experiment. Statistical comparisons not discussed in main text are omitted for clarity and are provided in Supplementary Data. Scale bars: 0.5 µm.

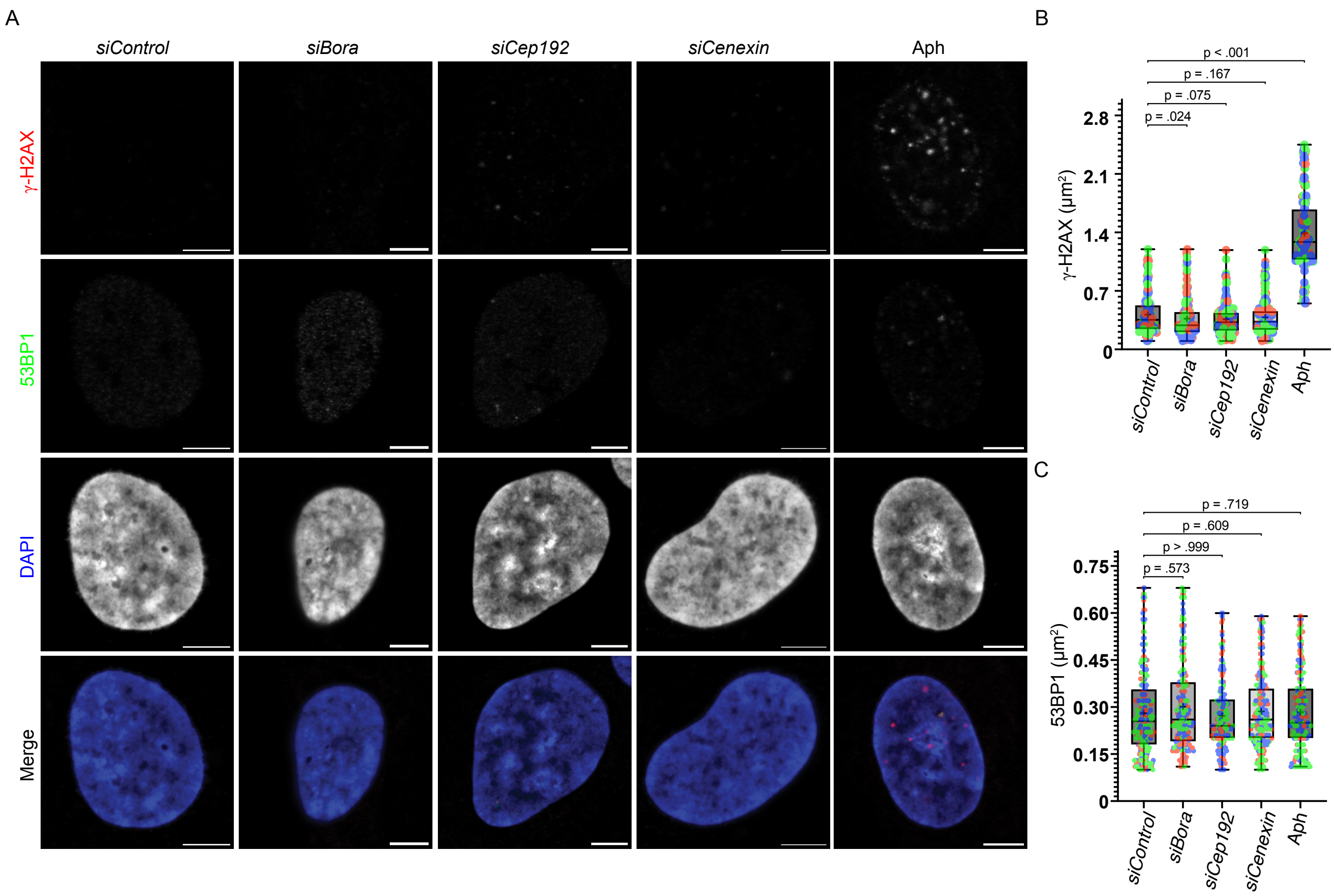

Figure S8: Depletion of Plk1 activators did not induce replication stress. (A) Representative images of hTERT-RPE1 cells treated with indicated siRNA/drugs and stained for indicated DNA damage marker proteins. **(B)** Quantification for the size of γ-H2AX nuclear puncta shown in **(A)**; N=3, n= 186: siControl, 184: siBora, 186: siCep192, 187: siCenexin and 166: Aphidicolin treated cells. **(C)** Quantification for the size of 53BP1 nuclear puncta shown in **(A)**; N=3, n= 228: siControl, 219: siBora, 169: siCep192, 223: siCenexin and 227: Aphidicolin treated cells. p-values: Kruskal-Wallis test (two-sided) (**B** and **C**) are indicated. The boxes indicate 1st quartile (minima), median (centre) and 3rd quartiles (maxima) and whiskers represent minimum and maximum values. Overlaid dots represent individual observations and '+' sign represent mean value. Dots of same colour represent cells derived from same experiment. Scale bars: 0.5 μm.

Supplementary Tables: Summary of Statistical tests

Table 1: Fig 1C and S1C: Cellular Plk1 activity in G2 Phase (Kruskal-Wallis test)

Condition	Significant ?	Summary	p - Value
**siControl** vs. **siBora**	Yes	***	<.001
**siControl** vs. **siCEP192**	No	ns	0.128
**siControl** vs. **siCenexin**	No	ns	0.538
**siControl** vs. **siAurora-A**	Yes	*	0.016
**siControl** vs. **siPlk1**	Yes	*	0.026
**siControl** vs. **Plk1i**	Yes	***	<.001
**siBora** vs. **siCEP192**	Yes	***	<.001
**siBora** vs. **siCenexin**	Yes	*	0.013
**siBora** vs. **siBora + siCEP192**	No	ns	0.976
**siBora** vs. **siBora + siCenexin**	No	ns	0.769
**siBora** vs. **siBora + siCEP192 + siCenexin**	No	ns	0.164
**siCEP192** vs. **siCenexin**	Yes	*	0.033
**siCEP192** vs. **siCEP192 + siCenexin**	No	ns	0.997
**siCEP192** vs. **siBora + siCEP192**	No	ns	0.173
**siCEP192** vs. **siBora + siCEP192 + siCenexin**	Yes	*	0.033
**siCenexin** vs. **siCEP192 + siCenexin**	Yes	*	0.045
**siCenexin** vs. **siBora + siCenexin**	No	ns	0.052
**siCenexin** vs. **siBora + siCEP192 + siCenexin**	Yes	***	<.001
**siControl** vs. **siBora + siCEP192**	Yes	***	<.001
**siControl** vs. **siBora + siCenexin**	Yes	***	<.001
**siControl** vs. **siCEP192 + siCenexin**	No	ns	0.075
**siBora + siCEP192** vs. **siBora + siCEP192 + siCenexin**	No	ns	0.719
**siBora + siCenexin** vs. **siBora + siCEP192 + siCenexin**	No	ns	0.200
**siCEP192 + siCenexin** vs. **siBora + siCEP192 + siCenexin**	No	ns	0.073

Table 2: Fig 1E and S1D: Centrosomal Plk1 activity in G2 Phase (Kruskal-Wallis test)

Condition	Significant ?	Summary	p - Value
**siControl** vs. **siBora**	No	ns	>.999
**siControl** vs. **siCEP192**	No	ns	0.146
**siControl** vs. **siCenexin**	Yes	*	0.026
**siControl** vs. **siAurora-A**	Yes	***	<.001
**siControl** vs. **siPlk1**	Yes	***	<.001
**siControl** vs. **Plk1i**	Yes	***	<.001
**siControl** vs. **siCEP192 + siCenexin**	Yes	***	<.001
**siBora** vs. **siCEP192**	No	ns	>.999
**siBora** vs. **siCenexin**	No	ns	>.999
**siCEP192** vs. **siCenexin**	No	ns	>.999
**siBora** vs. **siBora + siCEP192**	No	ns	>.999
**siBora** vs. **siCEP192 + siCenexin**	Yes	***	<.001
**siCEP192** vs. **siBora + siCEP192**	No	ns	>.999
**siCEP192** vs. **siBora + siCenexin**	No	ns	>.999
**siCenexin** vs. **siCEP192 + siCenexin**	Yes	**	0.002
**siCenexin** vs. **siBora + siCenexin**	No	ns	>.999
**siBora** vs. **siBora + siCEP192 + siCenexin**	Yes	***	<.001
**siCEP192** vs. **siBora + siCEP192 + siCenexin**	Yes	***	<.001
**siCenexin** vs. **siBora + siCEP192 + siCenexin**	Yes	***	<.001
**siBora + siCEP192** vs. **siBora + siCEP192 + siCenexin**	Yes	***	<.001
**siCEP192 + siCenexin** vs. **siBora + siCEP192 + siCenexin**	No	ns	0.374

Supplementary Tables: Summary of Statistical tests

Condition	Significant ?	Summary	p - Value
**siBora + siCenexin** vs. **siBora + siCEP192 + siCenexin**	Yes	***	<.001

Table 3: Fig 2J: Mobile Plk1 Pool (Kruskal-Wallis test)

Condition	Significant ?	Summary	p - Value
**siControl vs. siCep192**	Yes	**	0.001
**siControl vs. siCenexin**	No	ns	0.125
**siControl vs. siCep192+ siCenexin**	Yes	***	<.001
**siCep192 vs. siCenexin**	No	ns	0.965
**siCep192 vs. siCep192+ siCenexin**	No	ns	0.084
**siCenexin vs. siCep192+ siCenexin**	Yes	***	<.001

Table 4: Fig 2M and S2: Centrosomal Plk1 Levels in G2 (Kruskal-Wallis test)

Condition	Significant ?	Summary	p - Value
**siControl** vs. **siBora**	No	ns	>.999
**siControl** vs. **siCep192**	No	ns	>.999
**siControl** vs. **siCenexin**	No	ns	0.068
**siControl** vs. **siBora+ siCep192**	No	ns	>.999
**siControl** vs. **siCep192+ siCenexin**	Yes	**	0.004
**siControl** vs. **siBora+ siCenexin**	No	ns	>.999
**siControl** vs. **siBora+ siCep192+ siCenexin**	Yes	**	0.005
**siBora** vs. **siBora+ siCep192**	No	ns	>.999
**siBora** vs. **siBora+ siCenexin**	No	ns	>.999
**siCep192** vs. **siBora+ siCep192**	No	ns	>.999
**siCep192** vs. **siCep192+ siCenexin**	No	ns	0.942
**siCenexin** vs. **siBora+ siCep192**	No	ns	>.999
**siCenexin** vs. **siCep192+ siCenexin**	No	ns	>.999

Table 5: Fig 3C and S3B: S Phase Duration after depletion of Plk1 activators (Kruskal-Wallis test)

Condition	Significant ?	Summary	p - Value
siControl vs. siBora	Yes	***	<.001
siControl vs. siCyclin-A2	Yes	***	<.001
siControl vs. siCEP192	Yes	***	<.001
siControl vs. siCenexin	No	ns	0.180
siBora vs. siCEP192	Yes	***	<.001
siBora vs. siCenexin	No	ns	0.553
siCEP192 vs. siCenexin	Yes	***	<.001
siBora vs. siBora + siCEP192	Yes	**	0.003
siBora vs. siBora + siCenexin	Yes	***	<.001
siCEP192 vs. siBora + siCEP192	No	ns	>.999
siCEP192 vs. siCEP192 + siCenexin	No	ns	>.999
siCenexin vs. siBora + siCenexin	Yes	***	<.001

Supplementary Tables: Summary of Statistical tests

Condition	Significant ?	Summary	p - Value
siCenexin vs. siCEP192 + siCenexin	Yes	***	<.001
siBora vs. siBora + siCEP192 + siCenexin	Yes	***	<.001
siCEP192 vs. siBora + siCEP192 + siCenexin	No	ns	>.999
siCenexin vs. siBora + siCEP192 + siCenexin	Yes	***	<.001
siControl vs. siAurora-A	Yes	***	<.001
siControl vs. siPlk1	Yes	***	<.001

Table 6: Fig 3E and S3D: G2 Phase Duration after depletion of Plk1 activators (Kruskal-Wallis test)

Condition	Significant ?	Summary	p - Value
siControl vs. siBora	Yes	***	<.001
siControl vs. siCyclin-A2	Yes	***	<.001
siControl vs. siCEP192	No	ns	0.155
siControl vs. siCenexin	No	ns	>.999
siBora vs. siCEP192	Yes	***	<.001
siBora vs. siCenexin	Yes	***	<.001
siCEP192 vs. siCenexin	No	ns	>.999
siBora vs. siBora + siCEP192	No	ns	0.342
siBora vs. siBora + siCenexin	No	ns	>.999
siCEP192 vs. siBora + siCEP192	Yes	***	<.001
siCEP192 vs. siCEP192 + siCenexin	No	ns	>.999
siCenexin vs. siBora + siCenexin	Yes	***	<.001
siCenexin vs. siCEP192 + siCenexin	No	ns	>.999
siBora vs. siBora + siCEP192 + siCenexin	No	ns	>.999
siCEP192 vs. siBora + siCEP192 + siCenexin	Yes	***	<.001
siCenexin vs. siBora + siCEP192 + siCenexin	Yes	***	<.001
siControl vs. siAurora-A	Yes	***	<.001
siControl vs. siPlk1	Yes	***	<.001

Table 7: Fig 4D: Percentage of G1 cells after ectopic Cep192 expression in Cep192 RNAi cells (Ordinary one way ANNOVA)

Condition	Significant ?	Summary	p - Value
**siControl + Vector** vs. **siCep192 + Vector**	Yes	***	<.001
**siControl + Vector** vs. **siControl + Cep192* WT**	No	ns	>.999
**siControl + Vector** vs. **siCep192 + Cep192* WT**	No	ns	>.999
**siControl + Vector** vs. **siControl + Cep192* 2A**	No	ns	0.994
**siControl + Vector** vs. **siCep192 + Cep192* 2A**	Yes	*	0.044
**siCep192 + Vector** vs. **siControl + Cep192* WT**	Yes	***	<.001
**siCep192 + Vector** vs. **siCep192 + Cep192* WT**	Yes	***	<.001
**siCep192 + Vector** vs. **siControl + Cep192* 2A**	Yes	***	<.001
**siCep192 + Vector** vs. **siCep192 + Cep192* 2A**	No	ns	0.181
**siControl + Cep192* WT** vs. **siCep192 + Cep192* WT**	No	ns	>.999
**siControl + Cep192* WT** vs. **siControl + Cep192* 2A**	No	ns	0.977

Supplementary Tables: Summary of Statistical tests

Condition	Significant ?	Summary	p - Value
**siControl + Cep192* WT** vs. **siCep192 + Cep192* 2A**	Yes	*	0.029
**siCep192 + Cep192* TW** vs. **siControl + Cep192* 2A**	No	ns	0.997
**siCep192 + Cep192* TW** vs. **siCep192 + Cep192* 2A**	No	ns	0.052
**siControl + Cep192* 2A** vs. **siCep192 + Cep192* 2A**	No	ns	0.428

Table 8: Fig 4D: Percentage of S phase cells after ectopic Cep192 expression in Cep192 RNAi cells (Ordinary one way ANNOVA)

Condition	Significant ?	Summary	p - Value
**siControl + Vector** vs. **siCep192 + Vector**	Yes	***	<.001
**siControl + Vector** vs. **siControl + Cep192* WT**	No	ns	0.999
**siControl + Vector** vs. **siCep192 + Cep192* WT**	No	ns	>.999
**siControl + Vector** vs. **siControl + Cep192* 2A**	No	ns	0.212
**siControl + Vector** vs. **siCep192 + Cep192* 2A**	Yes	***	<.001
**siCep192 + Vector** vs. **siControl + Cep192* WT**	Yes	***	<.001
**siCep192 + Vector** vs. **siCep192 + Cep192* WT**	Yes	***	<.001
**siCep192 + Vector** vs. **siControl + Cep192* 2A**	Yes	***	<.001
**siCep192 + Vector** vs. **siCep192 + Cep192* 2A**	No	ns	>.999
**siControl + Cep192* WT** vs. **siCep192 + Cep192* WT**	No	ns	>.999
**siControl + Cep192* WT** vs. **siControl + Cep192* 2A**	No	ns	0.825
**siControl + Cep192* WT** vs. **siCep192 + Cep192* 2A**	Yes	***	<.001
**siCep192 + Cep192* TW** vs. **siControl + Cep192* 2A**	No	ns	0.721
**siCep192 + Cep192* TW** vs. **siCep192 + Cep192* 2A**	Yes	***	<.001
**siControl + Cep192* 2A** vs. **siCep192 + Cep192* 2A**	Yes	***	<.001

Table 9: Fig 4D: Percentage of G2/M cells after ectopic Cep192 expression in Cep192 RNAi cells (Ordinary one way ANNOVA)

Condition	Significant ?	Summary	p - Value
**siControl + Vector** vs. **siCep192 + Vector**	Yes	***	<.001
**siControl + Vector** vs. **siControl + Cep192* WT**	No	ns	>.999
**siControl + Vector** vs. **siCep192 + Cep192* WT**	No	ns	>.999
**siControl + Vector** vs. **siControl + Cep192* 2A**	No	ns	>.999
**siControl + Vector** vs. **siCep192 + Cep192* 2A**	Yes	***	<.001
**siCep192 + Vector** vs. **siControl + Cep192* WT**	Yes	***	<.001
**siCep192 + Vector** vs. **siCep192 + Cep192* WT**	Yes	***	<.001
**siCep192 + Vector** vs. **siControl + Cep192* 2A**	Yes	***	<.001
**siCep192 + Vector** vs. **siCep192 + Cep192* 2A**	No	ns	0.982
**siControl + Cep192* WT** vs. **siCep192 + Cep192* WT**	No	ns	>.999
**siControl + Cep192* WT** vs. **siControl + Cep192* 2A**	No	ns	>.999
**siControl + Cep192* WT** vs. **siCep192 + Cep192* 2A**	Yes	***	<.001
**siCep192 + Cep192* TW** vs. **siControl + Cep192* 2A**	No	ns	>.999
**siCep192 + Cep192* TW** vs. **siCep192 + Cep192* 2A**	Yes	***	<.001
**siControl + Cep192* 2A** vs. **siCep192 + Cep192* 2A**	Yes	***	<.001

Supplementary Tables: Summary of Statistical tests

Table 10: Fig 4F: Cellular Plk1 Activity in S Phase after depletion of Plk1 activators (Kruskal-Wallis test)

Condition	Significant ?	Summary	p - Value
siControl-G2 vs. ***siControl***	Yes	**	0.002
siControl vs. ***siBora***	No	ns	>.999
siControl vs. ***siCEP192***	Yes	**	0.007
siControl vs. ***siCenexin***	No	ns	0.868
siControl vs. **Plk1i**	Yes	**	0.008
siBora vs. ***siCEP192***	Yes	**	0.006
siBora vs. ***siCenexin***	No	ns	>.999
siBora vs. **Plk1i**	Yes	**	0.006
siCEP192 vs. ***siCenexin***	Yes	*	0.023
siCEP192 vs. **Plk1i**	No	ns	>.999
siCenexin vs. **Plk1i**	Yes	*	0.027

Table 11: Fig 4H: Centrosomal Plk1 Activity in S Phase after depletion of Plk1 activators (Kruskal-Wallis test)

Condition	Significant ?	Summary	p - Value
siControl-G2 vs. ***siControl***	Yes	*	0.018
siControl vs. ***siBora***	No	ns	>.999
siControl vs. ***siCEP192***	Yes	***	<.001
siControl vs. ***siCenexin***	No	ns	0.901
siControl vs. **Plk1i**	Yes	***	<.001
siBora vs. ***siCEP192***	Yes	**	0.005
siBora vs. ***siCenexin***	No	ns	>.999
siBora vs. **Plk1i**	Yes	**	0.009
siCEP192 vs. ***siCenexin***	Yes	***	<.001
siCEP192 vs. **Plk1i**	No	ns	0.126
siCenexin vs. **Plk1i**	Yes	***	<.001

Table 12: Fig. 5S: Rescue in PCNA nuclear foci with kinase dead Plk1 (K28R) after Aurora Ai

Condition	Significant ?	Summary	p - Value
**DMSO** vs. **DMSO + Doxycycline**	No	ns	0.291
**DMSO** vs. **Aurora Ai**	Yes	***	<.001
**DMSO + Doxycycline** vs. **Aurora Ai + Doxycycline**	Yes	***	<.001
**Aurora Ai** vs. **Aurora Ai + Doxycycline**	No	ns	>.999
**DMSO** vs. **Aurora Ai + Doxycycline**	Yes	***	<.001

Table 13: Fig. 5T: Rescue in PCNA nuclear foci with constitutively active Plk1 (T210D) after Aurora Ai

Condition	Significant ?	Summary	p - Value
**DMSO** vs. **DMSO + Doxycycline**	No	ns	>.999
**DMSO** vs. **Aurora Ai**	Yes	***	<.001
**DMSO + Doxycycline** vs. **Aurora Ai + Doxycycline**	No	ns	>.999

Supplementary Tables: Summary of Statistical tests

Condition	Significant ?	Summary	p - Value
**Aurora Ai** vs. **Aurora Ai + Doxycycline**	Yes	***	<.001
**DMSO** vs. **Aurora Ai + Doxycycline**	No	ns	>.999

Table 14: Fig. 6C: Checkpoint recovery in depletion of Plk1 activators (Kruskal-Wallis test)

Condition	Significant ?	Summary	p - Value
**siControl** vs. **siBora**	Yes	***	<.001
**siControl** vs. **siCEP192**	Yes	***	<.001
**siControl** vs. **siCenexin**	Yes	*	0.010
**siControl** vs. **siPlk1**	Yes	***	<.001
**siCEP192** vs. **siCEP192 + siCenexin**	No	ns	0.963
**siCenexin** vs. **siCEP192 + siCenexin**	Yes	***	<.001
**siBora** vs. **siPlk1**	No	ns	>.999
**siCEP192** vs. **siPlk1**	Yes	*	0.030
**siCenexin** vs. **siPlk1**	Yes	***	<.001

Table 15: Fig. 6G and S6C : Cell cycle progression without DNA damage after depletion of Plk1 activators (One-way ANNOVA)

Condition	Significant ?	Summary	p - Value
**siControl** vs. **siBora**	Yes	***	<.001
**siControl** vs. **siCep192**	Yes	***	<.001
**siControl** vs. **siCenexin**	No	ns	>.999
**siControl** vs. **siBora+ siCep192**	Yes	***	<.001
**siControl** vs. **siCep192+ siCenexin**	Yes	***	<.001
**siControl** vs. **siCenexin+ siBora**	Yes	***	<.001
**siControl** vs. **siBora+ siCep192+ siCenexin**	Yes	***	<.001
**siControl** vs. **siPlk1**	Yes	***	<.001
**siBora** vs. **siCep192**	Yes	***	<.001
**siBora** vs. **siCenexin**	Yes	**	0.005
**siBora** vs. **siBora+ siCep192**	Yes	***	<.001
**siBora** vs. **siCep192+ siCenexin**	Yes	***	<.001
**siBora** vs. **siCenexin+ siBora**	No	ns	>.999
**siBora** vs. **siBora+ siCep192+ siCenexin**	Yes	***	<.001
**siBora** vs. **siPlk1**	Yes	***	<.001
**siCep192** vs. **siCenexin**	Yes	***	<.001
**siCep192** vs. **siBora+ siCep192**	No	ns	>.999
**siCep192** vs. **siCep192+ siCenexin**	No	ns	>.999
**siCep192** vs. **siCenexin+ siBora**	Yes	***	<.001
**siCep192** vs. **siBora+ siCep192+ siCenexin**	No	ns	>.999
**siCep192** vs. **siPlk1**	No	ns	>.999
**siCenexin** vs. **siBora+ siCep192**	Yes	***	<.001
**siCenexin** vs. **siCep192+ siCenexin**	Yes	***	<.001
**siCenexin** vs. **siCenexin+ siBora**	Yes	**	0.002
**siCenexin** vs. **siBora+ siCep192+ siCenexin**	Yes	***	<.001

Supplementary Tables: Summary of Statistical tests

Condition	Significant ?	Summary	p - Value
**siCenexin** vs. **siPlk1**	Yes	***	<.001
**siBora+ siCep192** vs. **siCep192+ siCenexin**	No	ns	>.999
**siBora+ siCep192** vs. **siCenexin+ siBora**	Yes	***	0.001
**siBora+ siCep192** vs. **siBora+ siCep192+ siCenexin**	No	ns	0.921
**siBora+ siCep192** vs. **siPlk1**	No	ns	>.999
**siCep192+ siCenexin** vs. **siCenexin+ siBora**	Yes	***	<.001
**siCep192+ siCenexin** vs. **siBora+ siCep192+ siCenexin**	No	ns	>.999
**siCep192+ siCenexin** vs. **siPlk1**	No	ns	>.999
**siCenexin+ siBora** vs. **siBora+ siCep192+ siCenexin**	Yes	***	<.001
**siCenexin+ siBora** vs. **siPlk1**	Yes	***	<.001
**siBora+ siCep192+ siCenexin** vs. **siPlk1**	No	ns	0.998

Table 16: Fig 7B and S7B: γ-tubulin levels on centrosomes in prometaphase cells after depletion of Plk1 activators (Kruskal-Wallis test)

Condition	Significant ?	Summary	p - Value
**siControl** vs. **siBora**	Yes	*	0.025
**siControl** vs. **siCEP192**	Yes	*	0.042
**siControl** vs. **siCenexin**	No	ns	>.999
**siControl** vs. **siBora+ siCEP192**	Yes	**	0.006
**siControl** vs. **siCEP192+ siCenexin**	Yes	*	0.031
**siControl** vs. **siCenexin+ siBora**	Yes	*	0.026
**siControl** vs. **siBora+ siCEP192+ siCenexin**	Yes	*	0.024
**siControl** vs. **siPlk1**	Yes	*	0.026
**siBora** vs. **siCEP192**	Yes	*	0.028
**siBora** vs. **siCenexin**	Yes	**	0.001
**siBora** vs. **siBora+ siCEP192**	No	ns	>.999
**siBora** vs. **siCEP192+ siCenexin**	No	ns	0.058
**siBora** vs. **siCenexin+ siBora**	No	ns	0.508
**siBora** vs. **siBora+ siCEP192+ siCenexin**	No	ns	0.998
**siBora** vs. **siPlk1**	No	ns	>.999
**siCEP192** vs. **siCenexin**	Yes	*	0.012
**siCEP192** vs. **siBora+ siCEP192**	No	ns	0.054
**siCEP192** vs. **siCEP192+ siCenexin**	Yes	*	0.013
**siCEP192** vs. **siCenexin+ siBora**	Yes	***	<.001
**siCEP192** vs. **siBora+ siCEP192+ siCenexin**	Yes	*	0.034
**siCEP192** vs. **siPlk1**	Yes	*	0.042
**siCenexin** vs. **siBora+ siCEP192**	Yes	***	<.001
**siCenexin** vs. **siCEP192+ siCenexin**	Yes	**	0.009
**siCenexin** vs. **siCenexin+ siBora**	Yes	**	0.007
**siCenexin** vs. **siBora+ siCEP192+ siCenexin**	Yes	**	0.001
**siCenexin** vs. **siPlk1**	Yes	**	0.001
**siBora+ siCEP192** vs. **siCEP192+ siCenexin**	No	ns	0.122
**siBora+ siCEP192** vs. **siCenexin+ siBora**	No	ns	0.705

Supplementary Tables: Summary of Statistical tests

Condition	Significant ?	Summary	p - Value
**siBora+ siCEP192** vs. **siBora+ siCEP192+ siCenexin**	No	ns	0.986
**siBora+ siCEP192** vs. **siPlk1**	No	ns	>.999
**siCEP192+ siCenexin** vs. **siCenexin+ siBora**	Yes	*	0.032
**siCEP192+ siCenexin** vs. **siBora+ siCEP192+ siCenexin**	Yes	*	0.027
**siCEP192+ siCenexin** vs. **siPlk1**	No	ns	0.111
**siCenexin+ siBora** vs. **siBora+ siCEP192+ siCenexin**	No	ns	0.367
**siCenexin+ siBora** vs. **siPlk1**	No	ns	0.850
**siBora+ siCEP192+ siCenexin** vs. **siPlk1**	No	ns	0.940

Table 17: Fig 7B and S7B: Pericentrin levels on centrosomes in prometaphase cells after depletion of Plk1 activators (Kruskal-Wallis test)

Condition	Significant ?	Summary	p - Value
**siControl** vs. **siBora**	Yes	***	<.001
**siControl** vs. **siCEP192**	Yes	*	0.017
**siControl** vs. **siCenexin**	No	ns	0.999
**siControl** vs. **siBora+ siCEP192**	Yes	***	<.001
**siControl** vs. **siCEP192+ siCenexin**	Yes	*	0.012
**siControl** vs. **siCenexin+ siBora**	Yes	***	<.001
**siControl** vs. **siBora+ siCEP192+ siCenexin**	Yes	***	<.001
**siControl** vs. **siPlk1**	Yes	***	<.001
**siBora** vs. **siCEP192**	No	ns	0.295
**siBora** vs. **siCenexin**	Yes	***	<.001
**siBora** vs. **siBora+ siCEP192**	No	ns	>.999
**siBora** vs. **siCEP192+ siCenexin**	No	ns	0.404
**siBora** vs. **siCenexin+ siBora**	No	ns	>.999
**siBora** vs. **siBora+ siCEP192+ siCenexin**	No	ns	>.999
**siBora** vs. **siPlk1**	No	ns	>.999
**siCEP192** vs. **siCenexin**	No	ns	0.337
**siCEP192** vs. **siBora+ siCEP192**	No	ns	0.987
**siCEP192** vs. **siCEP192+ siCenexin**	No	ns	>.999
**siCEP192** vs. **siCenexin+ siBora**	No	ns	0.217
**siCEP192** vs. **siBora+ siCEP192+ siCenexin**	No	ns	0.106
**siCEP192** vs. **siPlk1**	Yes	*	0.034
**siCenexin** vs. **siBora+ siCEP192**	Yes	*	0.010
**siCenexin** vs. **siCEP192+ siCenexin**	No	ns	0.241
**siCenexin** vs. **siCenexin+ siBora**	Yes	***	<.001
**siCenexin** vs. **siBora+ siCEP192+ siCenexin**	Yes	***	<.001
**siCenexin** vs. **siPlk1**	Yes	***	<.001
**siBora+ siCEP192** vs. **siCEP192+ siCenexin**	No	ns	0.998
**siBora+ siCEP192** vs. **siCenexin+ siBora**	No	ns	>.999
**siBora+ siCEP192** vs. **siBora+ siCEP192+ siCenexin**	No	ns	0.976
**siBora+ siCEP192** vs. **siPlk1**	No	ns	0.727
**siCEP192+ siCenexin** vs. **siCenexin+ siBora**	No	ns	0.306

Supplementary Tables: Summary of Statistical tests

Condition	Significant ?	Summary	p - Value
**siCEP192+ siCenexin** vs. **siBora+ siCEP192+ siCenexin**	No	ns	0.155
**siCEP192+ siCenexin** vs. **siPlk1**	No	ns	0.051
**siCenexin+ siBora** vs. **siBora+ siCEP192+ siCenexin**	No	ns	>.999
**siCenexin+ siBora** vs. **siPlk1**	No	ns	>.999
**siBora+ siCEP192+ siCenexin** vs. **siPlk1**	No	ns	>.999

Table 18: Fig 7E: Rescue in γ-tubulin levels on centrosomes in prometaphase cells with RNAi-resistant Bora in Bora siRNA treated cells (Kruskal-Wallis test)

Condition	Significant ?	Summary	p - Value
**siControl + Vector** vs. **siBora + Vector**	Yes	***	<.001
**siControl + Vector** vs. **siControl + Bora WT Rescue**	No	ns	0.995
**siControl + Vector** vs. **siBora + Bora WT Rescue**	No	ns	0.413
**siBora + Vector** vs. **siControl + Bora WT Rescue**	Yes	**	0.001
**siBora + Vector** vs. **siBora + Bora WT Rescue**	Yes	**	0.005
**siControl + Bora WT Rescue** vs. **siBora + Bora WT Rescue**	No	ns	0.532

Table 19: Fig 7F: Rescue in Pericentrin levels on centrosomes in prometaphase cells with RNAi-resistant Bora in Bora siRNA treated cells (Kruskal-Wallis test)

Condition	Significant ?	Summary	p - Value
**siControl + Vector** vs. **siBora + Bora WT Rescue**	Yes	***	<.001
**siControl + Vector** vs. **siControl + Bora WT Rescue**	No	ns	0.918
**siControl + Vector** vs. **siBora + Bora WT Rescue**	No	ns	0.888
**siBora + Vector** vs. **siControl + Bora WT Rescue**	Yes	***	<.001
**siBora + Vector** vs. **siBora + Bora WT Rescue**	Yes	***	<.001
**siControl + Bora WT Rescue** vs. **siBora + Bora WT Rescue**	No	ns	>.999

Table 20: Fig 7H: Rescue in γ-tubulin levels on centrosomes in prometaphase cells with RNAi-resistant Cep192 in Cep192 siRNA treated cells (Kruskal-Wallis test)

Condition	Significant ?	Summary	p - Value
**siControl + Vector** vs. **siCep192 + Vector**	Yes	***	<.001
**siControl + Vector** vs. **siControl + Cep192* WT**	No	ns	0.617
**siControl + Vector** vs. **siCep192 + Cep192* WT**	No	ns	0.435
**siControl + Vector** vs. **siControl + Cep192* 2A**	No	ns	0.323
**siControl + Vector** vs. **siCep192 + Cep192* 2A**	Yes	***	<.001
**siCep192 + Vector** vs. **siControl + Cep192* WT**	Yes	***	<.001
**siCep192 + Vector** vs. **siCep192 + Cep192* WT**	Yes	***	<.001
**siCep192 + Vector** vs. **siControl + Cep192* 2A**	Yes	***	<.001
**siCep192 + Vector** vs. **siCep192 + Cep192* 2A**	No	ns	0.087
**siControl + Cep192* WT** vs. **siCep192 + Cep192* WT**	No	ns	>.999
**siControl + Cep192* WT** vs. **siControl + Cep192* 2A**	No	ns	0.992
**siControl + Cep192* WT** vs. **siCep192 + Cep192* 2A**	Yes	**	0.001
**siCep192 + Cep192* WT** vs. **siControl + Cep192* 2A**	No	ns	>.999
**siCep192 + Cep192* WT** vs. **siCep192 + Cep192* 2A**	Yes	**	0.002

Supplementary Tables: Summary of Statistical tests

Condition	Significant ?	Summary	p - Value
**siControl + Cep192* 2A** vs. **siCep192 + Cep192* 2A**	Yes	**	0.003

Table 21: Fig 7I: Rescue in Pericentrin levels on centrosomes in prometaphase cells with RNAi-resistant Cep192 in Cep192 siRNA treated cells (Kruskal-Wallis test)

Condition	Significant ?	Summary	p - Value
**siControl + Vector** vs. **siCep192 + Vector**	Yes	**	0.007
**siControl + Vector** vs. **siControl + Cep192* WT**	No	ns	>.999
**siControl + Vector** vs. **siCep192 + Cep192* WT**	No	ns	>.999
**siControl + Vector** vs. **siControl + Cep192* 2A**	No	ns	>.999
**siControl + Vector** vs. **siCep192 + Cep192* 2A**	No	ns	0.195
**siCep192 + Vector** vs. **siControl + Cep192* WT**	Yes	**	0.006
**siCep192 + Vector** vs. **siCep192 + Cep192* WT**	Yes	**	0.007
**siCep192 + Vector** vs. **siControl + Cep192* 2A**	Yes	**	0.008
**siCep192 + Vector** vs. **siCep192 + Cep192* 2A**	No	ns	0.377
**siControl + Cep192* WT** vs. **siCep192 + Cep192* WT**	No	ns	>.999
**siControl + Cep192* WT** vs. **siControl + Cep192* 2A**	No	ns	>.999
**siControl + Cep192* WT** vs. **siCep192 + Cep192* 2A**	No	ns	0.167
**siCep192 + Cep192* WT** vs. **siControl + Cep192* 2A**	No	ns	>.999
**siCep192 + Cep192* WT** vs. **siCep192 + Cep192* 2A**	No	ns	0.214
**siControl + Cep192* 2A** vs. **siCep192 + Cep192* 2A**	No	ns	0.226
