# Supplementary material for: Bora, CEP192 and Cenexin regulate distinct Plk1-dependent cell and centrosome cycle transitions
Source: Nat Commun. 2026 Jun 30;17:8158. doi: 10.1038/s41467-026-75033-5 (PMC13458439; doi:10.1038/s41467-026-75033-5)
Supplement: Supplementary file 2 [file 41467_2026_75033_MOESM2_ESM.pdf]

Title: Supplementary Movie 1

Description: FRAP recordings of hTERT-RPE1 expressing endogenously tagged Plk1-EGFP and treated with siControl. Every image was captured at an interval of 5s. The Supplementary Movie is shown at 3 frames per second (fps). Scale bars, 5 µm.

Title: Supplementary Movie 2

Description: FRAP recordings of hTERT-RPE1 expressing endogenously tagged Plk1-EGFP and treated with siCep192. Every image was captured at an interval of 5s. The Supplementary Movie is shown at 3 frames per second (fps). Scale bars, 5 µm.

Title: Supplementary Movie 3

Description: FRAP recordings of hTERT-RPE1 expressing endogenousy tagged Plk1-EGFP and treated with siCenexin. Every image was captured at an interval of 5s. The Supplementary Movie is shown at 3 frames per second (fps). Scale bars, 5 µm.

Title: Supplementary Movie 4

Description: FRAP recordings of hTERT-RPE1 expressing endogenously tagged Plk1-EGFP and co-treated with siCep192 and siCenexin. Every image was captured at an interval of 5s. The Supplementary Movie is shown at 3 frames per second (fps). Scale bars, 5 µm.

Title: Supplementary Movie 5

Description: FRAP recordings of hTERT-RPE1 expressing endogenously tagged Plk1-EGFP and treated with DMSO/Control. Every image was captured at an interval of 4s. The Supplementary Movie is shown at 3 frames per second (fps). Scale bars, 5 µm.

Title: Supplementary Movie 6

Description: FRAP recordings of hTERT-RPE1 expressing endogenously tagged Plk1-EGFP and treated with Plk1i(BI2536). Every image was captured at an interval of 5s. The Supplementary Movie is shown at 3 frames per second (fps). Scale bars, 5 µm.

Title: Supplementary Movie 7

Description: Live-cell imaging of hTERT-RPE1 expressing endogenously tagged mRuby-PCNA and treated with siControl progressing through S phase. Every image was captured at an interval of 10 m. The Supplementary Movie is shown at 5 frames per second (fps). Scale bars, 5 µm.

Title: Supplementary Movie 8

Description: Live-cell imaging of hTERT-RPE1 expressing endogenously tagged mRuby-PCNA and treated with siControl progressing through G2 phase. Every image was captured at an interval of 10 m. The Supplementary Movie is shown at 5 frames per second (fps). Scale bars, 5 µm.

Title: Supplementary Movie 9

Description: Live-cell imaging of hTERT-RPE1 expressing endogenously tagged mRuby-PCNA and treated with siCyclin A2 progressing through S phase. Every image was captured at an interval of 10 m. The Supplementary Movie is shown at 5 frames per second (fps). Scale bars, 5 µm.

Title: Supplementary Movie 10

Description: Live-cell imaging of hTERT-RPE1 expressing endogenously tagged mRuby-PCNA and treated with siCyclin A2 progressing through G2 phase. Every image was captured at an interval of 10 m. The Supplementary Movie is shown at 5 frames per second (fps). Scale bars, 5 µm.

Title: Supplementary Movie 11

Description: Live-cell imaging of hTERT-RPE1 expressing endogenously tagged mRuby-PCNA and treated with siBora progressing through S phase. Every image was captured at an interval of 10 m. The Supplementary Movie is shown at 5 frames per second (fps). Scale bars, 5 µm.

Title: Supplementary Movie 12

Description: Live-cell imaging of hTERT-RPE1 expressing endogenously tagged mRuby-PCNA and treated with siCep192 progressing through S phase. Every image was captured at an interval of 10 m. The Supplementary Movie is shown at 5 frames per second (fps). Scale bars, 5 µm.

Title: Supplementary Movie 13

Description: Live-cell imaging of hTERT-RPE1 expressing endogenously tagged mRuby-PCNA and treated with siCenexin progressing through S phase. Every image was captured at an interval of 10 m. The Supplementary Movie is shown at 5 frames per second (fps). Scale bars, 5 µm.

Title: Supplementary Movie 14

Description: Live-cell imaging of hTERT-RPE1 expressing endogenously tagged mRuby-PCNA and co-treated with siBora and Cep192 progressing through S phase. Every image was captured at an interval of 10 m. The Supplementary Movie is shown at 5 frames per second (fps). Scale bars, 5 µm.

Title: Supplementary Movie 15

Description: Live-cell imaging of hTERT-RPE1 expressing endogenously tagged mRuby-PCNA and co-treated with siCep192 and siCenexin progressing through S phase. Every image was captured at

an interval of 10 m. The Supplementary Movie is shown at 5 frames per second (fps). Scale bars, 5 µm.

Title: Supplementary Movie 16

Description: Live-cell imaging of hTERT-RPE1 expressing endogenously tagged mRuby-PCNA and co-treated with siBora and siCenexin progressing through S phase. Every image was captured at an interval of 10 m. The Supplementary Movie is shown at 5 frames per second (fps). Scale bars, 5 µm.

Title: Supplementary Movie 17

Description: Live-cell imaging of hTERT-RPE1 expressing endogenously tagged mRuby-PCNA and co-treated with siBora, siCep192 and siCenexin altogether progressing through S phase. Every image was captured at an interval of 10 m. The Supplementary Movie is shown at 5 frames per second (fps). Scale bars, 5 µm.

Title: Supplementary Movie 18

Description: Live-cell imaging of hTERT-RPE1 expressing endogenously tagged mRuby-PCNA and treated with siPlk1 progressing through S phase. Every image was captured at an interval of 10 m. The Supplementary Movie is shown at 5 frames per second (fps). Scale bars, 5 µm.

Title: Supplementary Movie 19

Description: Live-cell imaging of hTERT-RPE1 expressing endogenously tagged mRuby-PCNA and treated with siBora progressing through G2 phase. Every image was captured at an interval of 10 m. The Supplementary Movie is shown at 5 frames per second (fps). Scale bars, 5 µm.

Title: Supplementary Movie 20

Description: Live-cell imaging of hTERT-RPE1 expressing endogenously tagged mRuby-PCNA and treated with siCep192 progressing through G2 phase. Every image was captured at an interval of 10 m. The Supplementary Movie is shown at 5 frames per second (fps). Scale bars, 5 µm.

Title: Supplementary Movie 21

Description: Live-cell imaging of hTERT-RPE1 expressing endogenously tagged mRuby-PCNA and treated with siCenexin progressing through G2 phase. Every image was captured at an interval of 10 m. Supplementary Movie is shown at 5 frames per second (fps). Scale bars, 5 µm.

Title: Supplementary Movie 22

Description: Live-cell imaging of hTERT-RPE1 expressing endogenously tagged mRuby-PCNA and co-treated with siBora and Cep192 progressing through G2 phase. Every image was captured at an interval of 10 m. The Supplementary Movie is shown at 5 frames per second (fps). Scale bars, 5 µm.

Title: Supplementary Movie 23

Description: Live-cell imaging of hTERT-RPE1 expressing endogenously tagged mRuby-PCNA and co-treated with siCep192 and siCenexin progressing through G2 phase. Every image was captured at an interval of 10 m. The Supplementary Movie is shown at 5 frames per second (fps). Scale bars, 5 µm.

Title: Supplementary Movie 24

Description: Live-cell imaging of hTERT-RPE1 expressing endogenously tagged mRuby-PCNA and co-treated with siBora and siCenexin progressing through G2 phase. Every image was captured at an interval of 10 m. The Supplementary Movie is shown at 5 frames per second (fps). Scale bars, 5 µm.

Title: Supplementary Movie 25

Description: Live-cell imaging of hTERT-RPE1 expressing endogenously tagged mRuby-PCNA and co-treated with siBora, siCep192 and siCenexin altogether progressing through G2 phase. Every image was captured at an interval of 10 m. The Supplementary Movie is shown at 5 frames per second (fps). Scale bars, 5 µm.

Title: Supplementary Movie 26

Description: Live-cell imaging of hTERT-RPE1 expressing endogenously tagged mRuby-PCNA and treated with siPlk1 progressing through G2 phase. Every image was captured at an interval of 10 m. The Supplementary Movie is shown at 5 frames per second (fps). Scale bars, 5 µm.

Title: Supplementary Movie 27

Description: Live-cell imaging of hTERT-RPE1 expressing endogenously tagged mRuby-PCNA and treated with siAurora A progressing through S phase. Every image was captured at an interval of 10 m. The Supplementary Movie is shown at 5 frames per second (fps). Scale bars, 5 µm.

Title: Supplementary Movie 28

Description: Live-cell imaging of hTERT-RPE1 expressing endogenously tagged mRuby-PCNA and treated with siAurora A progressing through G2 phase. Every image was captured at an interval of 10 m. The Supplementary Movie is shown at 5 frames per second (fps). Scale bars, 5 µm.

Title: Supplementary Movie 29

Description: Live-cell imaging of hTERT-RPE1 expressing endogenously tagged mRuby-PCNA synchronised in G1 with Cdk4/6i, released and treated with DMSO progressing to enter S phase. Every image was captured at an interval of 10 m. The Supplementary Movie is shown at 5 frames per second (fps). Scale bars, 5 µm.

Title: Supplementary Movie 30

Description: Live-cell imaging of hTERT-RPE1 expressing endogenously tagged mRuby-PCNA synchronised in G1 with Cdk4/6i, released and treated with Aurora Ai progressing to enter S phase. Every image was captured at an interval of 10 m. The Supplementary Movie is shown at 5 frames per second (fps). Scale bars, 5 µm.

Title: Supplementary Movie 31

Description: Live-cell imaging of hTERT-RPE1 expressing endogenously tagged mRuby-PCNA synchronised in G1 with Cdk4/6i, released and treated with Plk1i progressing to enter S phase. Every image was captured at an interval of 10 m. The Supplementary Movie is shown at 5 frames per second (fps). Scale bars, 5 µm.

Title: Supplementary Movie 32

Description: Live-cell imaging of hTERT-RPE1 expressing endogenously tagged mRuby-PCNA synchronised in G1 with Cdk4/6i, released and treated with siControl progressing to enter S phase. Every image was captured at an interval of 10 m. The Supplementary Movie is shown at 5 frames per second (fps). Scale bars, 5 µm.

Title: Supplementary Movie 33

Description: Live-cell imaging of hTERT-RPE1 expressing endogenously tagged mRuby-PCNA synchronised in G1 with Cdk4/6i, released and treated with siCep192 progressing to enter S phase. Every image was captured at an interval of 10 m. The Supplementary Movie is shown at 5 frames per second (fps). Scale bars, 5 µm.

Title: Supplementary Movie 34

Live-cell imaging of hTERT-RPE1 expressing endogenously tagged Aurora A - mVenus synchronised in G1 with Cdk4/6i and released, progressing to enter S phase. The imaging was started 3 hrs post release, and every image was captured at an interval of 10 m. The Supplementary Movie is shown at 5 frames per second (fps). Scale bars, 5 µm.

Title: Supplementary Movie 35

Description: Live-cell imaging of hTERT-RPE1 expressing endogenously tagged Plk1-EGFP synchronised in G1 with Cdk4/6i and released, progressing to enter S phase. The imaging was started 3 hrs post release, and every image was captured at an interval of 10 m. The Supplementary Movie is shown at 5 frames per second (fps). Scale bars, 5 µm.
