# Supplementary material for: Bora, CEP192 and Cenexin regulate distinct Plk1-dependent cell and centrosome cycle transitions
Source: Nat Commun. 2026 Jun 30;17:8158. doi: 10.1038/s41467-026-75033-5 (PMC13458439; doi:10.1038/s41467-026-75033-5)
Supplement: Supplementary file 3 [file 41467_2026_75033_MOESM38_ESM.pdf]

nature portfolio

Corresponding author(s): Devashish Dwivedi and Patrick Meraldi

Last updated by author(s): Jun 15, 2026

Reporting Summary

Nature Portfolio wishes to improve the reproducibility of the work that we publish. This form provides structure for consistency and transparency in reporting. For further information on Nature Portfolio policies, see our Editorial Policies and the Editorial Policy Checklist.

Statistics

For all statistical analyses, confirm that the following items are present in the figure legend, table legend, main text, or Methods section.

n/a	Confirmed	
☐	☒	The exact sample size (*n*) for each experimental group/condition, given as a discrete number and unit of measurement
☐	☒	A statement on whether measurements were taken from distinct samples or whether the same sample was measured repeatedly
☐	☒	The statistical test(s) used AND whether they are one- or two-sided<br>*Only common tests should be described solely by name; describe more complex techniques in the Methods section.*
☒	☐	A description of all covariates tested
☐	☒	A description of any assumptions or corrections, such as tests of normality and adjustment for multiple comparisons
☐	☒	A full description of the statistical parameters including central tendency (e.g. means) or other basic estimates (e.g. regression coefficient) AND variation (e.g. standard deviation) or associated estimates of uncertainty (e.g. confidence intervals)
☐	☒	For null hypothesis testing, the test statistic (e.g. *F*, *t*, *r*) with confidence intervals, effect sizes, degrees of freedom and *P* value noted<br>*Give P values as exact values whenever suitable.*
☒	☐	For Bayesian analysis, information on the choice of priors and Markov chain Monte Carlo settings
☒	☐	For hierarchical and complex designs, identification of the appropriate level for tests and full reporting of outcomes
☒	☐	Estimates of effect sizes (e.g. Cohen's *d*, Pearson's *r*), indicating how they were calculated

Our web collection on statistics for biologists contains articles on many of the points above.

Software and code

Policy information about availability of computer code

Data collection	For FRET analysis Dual channel images were acquired on Spinning disk microscope (Nipkow disk) with Visiview 4.00.10 software (Visitron)<br>Expansion microscopy images were acquired on Deltavision microscope with Softworx 6.5.2 (GE Healthcare) software.<br>Immunoflourescence images were acquired on Deltavision microscope with Softworx 6.5.2 (GE Healthcare) software.<br>Live cell imaging data was acquired on Nikon Ti Microscope using NIS Elements 4.30.02 software (Nikon).<br>Flow Cytometry based Cell cycle profile was acquired on Beckman Coulter Cytoflex using CytExpert Software (Beckman Coulter)
Data analysis	All the Immuno-fluorescence images (IF, STED, U-ExM, and FRET) were analyzed in ImageJ/Fiji.<br>Flow Cytometry Data was analysed in Flow Jo Ver 10 (BD Bioscieices)<br>Statistical data were analyzed with Graphpad Prism 10 (Graphpad)

For manuscripts utilizing custom algorithms or software that are central to the research but not yet described in published literature, software must be made available to editors and reviewers. We strongly encourage code deposition in a community repository (e.g. GitHub). See the Nature Portfolio guidelines for submitting code & software for further information.

March 2021

Data

Policy information about availability of data

All manuscripts must include a data availability statement. This statement should provide the following information, where applicable:

- Accession codes, unique identifiers, or web links for publicly available datasets
- A description of any restrictions on data availability
- For clinical datasets or third party data, please ensure that the statement adheres to our policy

All the Raw data related to figures and supplementary figures (representative images and movies) will be made available

Human research participants

Policy information about studies involving human research participants and Sex and Gender in Research.

Reporting on sex and gender	N/A
Population characteristics	N/A
Recruitment	N/A
Ethics oversight	N/A

Note that full information on the approval of the study protocol must also be provided in the manuscript.

Field-specific reporting

Please select the one below that is the best fit for your research. If you are not sure, read the appropriate sections before making your selection.

[x] Life sciences   [ ] Behavioural & social sciences   [ ] Ecological, evolutionary & environmental sciences

For a reference copy of the document with all sections, see nature.com/documents/nr-reporting-summary-flat.pdf

Life sciences study design

All studies must disclose on these points even when the disclosure is negative.

Sample size	Sample size and number of biological repeats for each experiment are indicated in all figure legends or in the "Statistics and reproducibility" section. In all the experiments, at least 3 independent biological repeats were performed. In each case the sample size was chosen to fit the statistical test used for testing the null hypothesis.
Data exclusions	No data was excluded from the analysis.
Replication	All quantifiable experiments were replicated in at least 3 independent experiments (sometimes more than 3) to ensure the reproducibility of the results
Randomization	Our study is based solely on basic research. Moreover, due to the variations within the same condition and the morphological modifications following the different treatments used which would inevitably give us an indication of the condition analyzed, makes the randomization not applicable to our study. Therefore, no formal randomization techniques were used.<br>However, we performed all of our experiments as consistently as possible and most of our measurements are based on a large number of analyzed conditions.
Blinding	Blinding was not necessary during data collection (Image acquisition) as information regarding centriole disengagement or fluorescence intensity can only be obtained during Image analysis. All the quantifiable parameters were extracted from the images using automated image analysis softwares that excluded experimentator-induced biases, further rendering the usage of blinding approaches unnecessary. Furthermore, our study does not work with human subjects/patients/animals.

Reporting for specific materials, systems and methods

We require information from authors about some types of materials, experimental systems and methods used in many studies. Here, indicate whether each material, system or method listed is relevant to your study. If you are not sure if a list item applies to your research, read the appropriate section before selecting a response.

Materials & experimental systems

n/a	Involved in the study
[ ]	[x] Antibodies
[ ]	[x] Eukaryotic cell lines
[x]	[ ] Palaeontology and archaeology
[x]	[ ] Animals and other organisms
[x]	[ ] Clinical data
[x]	[ ] Dual use research of concern

Methods

n/a	Involved in the study
[x]	[ ] ChIP-seq
[ ]	[x] Flow cytometry
[x]	[ ] MRI-based neuroimaging

Antibodies

Antibodies used

- Mouse anti-α-tubulin (Geneva antibody facility: AA345-M2a; 1:250: ExM)
- Mouse anti-β-tubulin (Geneva antibody facility: AA344-M2a; 1:250: ExM)
- Mouse anti-α-tubulin (Clone DM1α; Sigma Aldrich, T9026, 1:5000: Western Blotting)
- Rabbit polyclonal anti-Pericentrin (abcam: ab4448; 1:250: ExM, 1:2000: IF)
- Mouse anti-Cyclin-A2 (Clone: E23.1, abcam: ab38; 1:1000: Western Blotting)
- Mouse anti-Aurora A (Clone 4/IAK1, BD Bioscieices: 610939: 1:2000: Western Blotting)
- Rabbit Polyclonal anti-Bora (Gift from Erich Nigg, 1:1000 Western blotting)
- Rabbit polyclonal anti-CEP192 (Bethyl: A302-324A, 1:1000 Western blotting),
- Rabbit Piolyclonal anti-Cenexin (abcam: ab43840, 1:1000 Western blotting)
- Mouse Monoclonal anti-Plk1 (Clone: 36-298, abcam: ab17056, 1:1000 Western blotting)
- Chicken Polyclonal anti-GFP (Thermo Fisher Scientific: A10262; 1:2000 IF)
- Mouse monoclonal anti-Separase (abcam: ab16170; 1:500 Western blotting)
- Mouse monoclonal anti-Centrin (Clone: 20H5, Merck Millipore: 04-1624 ; 1:1500 IF)
- Rabbit polyclonal anti-53BP1 (Novus Biologicals: NB100-304: 1:2000 IF)
- Mouse monoclonal anti-γ-tubulin (Clone: GTU-88, Sigma Aldrich: T6557, 1:2000 IF)
- Mouse monoclonal anti-PCNA (Clone: PC10, Santacruz Biotechnology, SC-56, 1:1000 IF)
- Mouse monoclonal anti-γ-H2AX pSer139 (Clone: JBW301, Merck Millipore, 05-636, 1:1500 IF)
- Mouse anti-PLK1 (pT210) (BD Pharmingen™ Purified) (Clone: K50-483: BD Biosciences, 558400, 1:200 IF)
- Mouse anti-GAPDH (Clone: GA1R, Thermo Fisher Scientific, MA5-15738: 1:2000 Western Blotting)
- Rabbit Polyclonal Anti-GFP Antibody (Origene: TP401, 1:5000 Western Blotting)

- - - Alexa Fluor–conjugated secondary antibodies in Immunofluorescence/U-ExM– Dilution 1:500:
- Donkey anti-Mouse IgG (H+L) Alexa Fluor 488 (Invitrogen; A-21202)
- Goat anti-Rabbit IgG (H+L) Alexa Fluor 488 (Invitrogen; A-11008)
- Goat anti-Rabbit IgG (H+L) Alexa Fluor 568 (Invitrogen; A-11036)
- Goat anti-Mouse IgG (H+L) Alexa Fluor 568 (Invitrogen; A-11031)
- Goat anti-Chicken IgG (H+L) Alexa Fluor 488 (Invitrogen: A-11039)

- - HRP-Conjugated secondary antibody in Western Blotting - Dilution 1:10,000
- Goat anti-Mouse antibody for western blotting (Thermo Fisher: Cat # 32430)
- Goat anti-Rabbit antibody for western Blotting (BioRad Laboratories: Cat # 1706515)

Validation

- Mouse anti-α-tubulin (Geneva antibody facility: AA345-M2a; 1:250: ExM) was previously tested and validated in the manuscript (doi:10.24450/journals/abrep.2019.e17)
- Mouse anti-β-tubulin (Geneva antibody facility: AA345-M2a; 1:250: ExM) was previously tested and validated in the manuscript (doi:10.24450/journals/abrep.2019.e17)
- Mouse anti-α-tubulin (Clone DM1α; Sigma Aldrich, T9026, 1:5000: Western Blotting) was previously validated by the manufacturer (https://www.sigmaaldrich.com/CH/en/product/sigma/t9026)
- Rabbit polyclonal anti-Pericentrin (abcam: ab4448; 1:250: ExM, 1:1000: STED) was previously validated by the manufacturer (https://www.abcam.com/pericentrin-antibody-centrosome-marker-ab4448.html)
- Mouse anti-Cyclin-A2 (Clone: E23.1, abcam: ab38; 1:1000: Western Blotting) was previously validated by manufacturer (https://www.abcam.com/cyclin-a2-antibody-e231-ab38.html)
- Mouse anti-Aurora A (Clone 4/IAK1, BD Biosciences: 610939: 1:2000: Western Blotting) was previously validated by manufacturer (https://www.bdbiosciences.com/en-ch/products/reagents/microscopy-imaging-reagents/immunofluorescence-reagents/purified-mouse-anti-iak1.610939?tab=product_details)
- Rabbit Polyclonal anti-Bora (Gift from Erich Nigg, 1:1000 Western blotting) and was previously reported, tested and validated in Chan et al., 2008 (PMID: 18521620)
- Rabbit polyclonal anti-CEP192 (Bethyl: A302-324A, 1:1000 Western blotting) was previously validated by manufacturer (https://www.fortislife.com/products/primary-antibodies/rabbit-anti-cep192-antibody/BETHYL-A302-324?selected=A302-324A)
- Rabbit Polyclonal anti-Cenexin (abcam: ab43840, 1:1000 Western blotting)was previously validated by manufacturer (https://www.abcam.com/en-us/products/primary-antibodies/cenexin1-odf2-antibody-ab43840)
- Mouse Monoclonal anti-Plk1 (Clone: 36-298, abcam: ab17057, 1:1000 Western blotting) was previously validated by manufacturer (https://www.abcam.com/en-us/products/primary-antibodies/plk1-antibody-36-298-ab17057)

- Chicken Polyclonal anti-GFP (Thermo Fisher Scientific: A10262; 1:2000 IF) was previously validated by manufacturer (https://www.thermofisher.com/antibody/product/GFP-Antibody-Polyclonal/A10262)
- Mouse monoclonal anti-Separase (abcam: ab16170; 1:500 Western blotting) was previously validated by manufacturer (https://www.abcam.com/en-us/products/primary-antibodies/separase-antibody-xj11-1b12-ab16170)
- Mouse monoclonal anti-Centrin (Clone: 20H5, Merck Millipore: 04-1624 ; 1:1500 IF) was previously validated by manufacturer (http://www.merckmillipore.com/CH/de/product/Anti-Centrin-Antibody-clone-20H5,MM_NF-04-1624?ReferrerURL=https%253A%252F%252Fwww.bing.com%252F&bd=1)
- Rabbit polyclonal anti-53BP1 (Novus Biologicals: NB100-304: 1:2000 IF) was previously validated by manufacturer (https://www.novusbio.com/products/53bp1-antibody_nb100-304)
- Mouse monoclonal anti-γ-tubulin (Clone: GTU-88, Sigma Aldrich: T6557, 1:2000 IF) was previously validated by manufacturer (https://www.sigmaaldrich.com/CH/en/product/sigma/t6557)
- Mouse monoclonal anti-PCNA (Clone: PC10, Santacruz Biotechnology, SC-56, 1:1000 IF) was previously validated by manufacturer (https://datasheets.scbt.com/sc-56.pdf)
- Mouse monoclonal anti-γ-H2AX pSer139 (Clone: JBW301, Merck Millipore, 05-636, 1:1500 IF) was previously validated by manufacturer (https://www.merckmillipore.com/CH/en/product/Anti-phospho-Histone-H2A.X-Ser139-Antibody-clone-JBW301,MM_NF-05-636)
- Mouse anti-PLK1 (pT210) (BD Pharmingen™ Purified) (Clone: K50-483: BD Biosciences, 558400, 1:200 IF) was previously validated by manufacturer (https://www.bdbiosciences.com/en-ch/products/reagents/western-blotting-and-molecular-reagents/western-blot-reagents/purified-mouse-anti-plk1-pt210.558400?tab=product_details)
- Mouse anti-GAPDH (Clone: GA1R, Thermo Fisher Scientific, MA5-15738: 1:2000 Western Blotting) was previously validated by manufacturer (https://www.thermofisher.com/antibody/product/GAPDH-Loading-Control-Antibody-clone-GA1R-Monoclonal/MA5-15738)
- Rabbit Polyclonal Anti-GFP Antibody (Origene: TP401, 1:5000 Western Blotting) was previously validated by manufacturer (https://www.origene.com/catalog/antibodies/primary-antibodies/tp401-gfp-rabbit-polyclonal-antibody

Eukaryotic cell lines

Policy information about cell lines and Sex and Gender in Research

Cell line source(s)

- hTERT-RPE1 cells were obtained from ATCC (ATCC; CRL-4000, CCL-2).
- hTERT-RPE1 Plk1-EGFP cell-line was generated in the lab for this study and the process is described in the materials and methods section.
- hTERT-RPE1 Plk1 FRET Sensor + Cyclin A2 mScarlet cell-line was generated in the lab for this study and the process is described in the materials and methods section.
- hTERT-RPE1 Centrosomal Plk1 FRET Sensor + Cyclin A2 mScarlet cell-line was generated in the lab for this study and the process is described in the materials and methods section.
- hTERT-RPE1 mRuby-PCNA Cell line was kindly provided by Alexis Barr, Imperial College London, United Kingdom and was described for the first time in Meitinger et al., 2016 (https://www.nature.com/articles/ncomms14728)
- hTERT-RPE1 USP28 Knockout Cell line was kindly provided by Arshad Desai, University of California, San Diego, United Stated and was described for the first time in Meitinger et al., 2016 (https://rupress.org/jcb/article/214/2/155/38581/53BP1-and-USP28-mediate-p53-activation-and-G1)
- hTERT-RPE1 Aurora A - mVenus was kindly provided by Cahterine Lindon, University of Cambridge, Cambridge, United Kingdom and was described for the first time in Grant et al., 2018 (https://royalsocietypublishing.org/rsob/article/8/6/170272/91656/Constitutive-regulation-of-mitochondrial)
- hTERT-RPE1 USP28 Knockout Centrosome less cell line was generated in the lab for this study and the process is described in the materials and methods section.
- hTERT-RPE1 Cenexin knockout cell line was kindly provided by Meng-Fu Bryan Tsou, Memorial Sloan-Kettering Cancer Center, New York, United States and was described for the first time in Mazo et al., 2016 (https://www.sciencedirect.com/science/article/pii/S1534580716307195?via=ihub)
- hTERT-RPE1 Centrosomal Separase Sensor cell-line was generated in the lab for this study and the process is described in the materials and methods section.
- hTERT-RPE1 EGFP-Bora RNAi resistant cell-line was generated in the lab for this study and the process is described in the materials and methods section.
- hTERT-RPE1 EGFP-Cep192 WT RNAi resistant cell-line was generated in the lab for this study and the process is described in the materials and methods section.
- hTERT-RPE1 EGFP-Cep192 2A (T44A, S995A) RNAi resistant cell-line was generated in the lab for this study and the process is described in the materials and methods section.
- hTERT-RPE1 FRT/TR EGFP-Plk1 K82R cell line was generated in the lab for this study and the process is described in the materials and methods section.
- hTERT-RPE1 FRT/TR EGFP-Plk1 T210D cell line was generated in the lab for this study and the process is described in the materials and methods section.
- hTERT-RPE1 Aurora A - mVenus + mScarlet3-PACT cell line was generated in the lab for this study and the process is described in the materials and methods section.
- hTERT-RPE1 Plk1-GFP + mScarlet3-PACT cell line was generated in the lab for this study and the process is described in the materials and methods section.

Authentication

None of the cell lines was authenticated

Mycoplasma contamination

All cell lines are tested for mycoplasma every 6 months and are negative for any mycoplasma contamination.

Commonly misidentified lines
(See ICLAC register)

All the cell lines used in this study are not included in the list of known misidentified cell lines from the International Cell Line Authentication Committee.

Flow Cytometry

Plots

Confirm that:

- [ ] The axis labels state the marker and fluorochrome used (e.g. CD4-FITC).
- [ ] The axis scales are clearly visible. Include numbers along axes only for bottom left plot of group (a 'group' is an analysis of identical markers).
- [ ] All plots are contour plots with outliers or pseudocolor plots.
- [x] A numerical value for number of cells or percentage (with statistics) is provided.

Methodology

Sample preparation	Asynchronously growing cells in log phase were collected by trypsinisation in a microcentrifuge tube, counted and fixed with prechilled 70% ethanol and incubated overnight at -20°C. Next day, the cells were washed with 1X PBS and resuspended in PI/RNase Staining Buffer (BD Biosciences: 550825) to a final concentration of 0.5 million cells per ml of suspension and incubated in dark for 1h at room temperature.
Instrument	Beckman Coulter Cytoflex
Software	CytExpert Software for the CytoFLEX Platform and Flow Jo 10
Cell population abundance	75-95%
Gating strategy	No specific gating strategy was adopted for data acquisition. Only Single cells were selected based on height vs Area plot of Propidium Iodide and only single cells were considered for analysis.

- [ ] Tick this box to confirm that a figure exemplifying the gating strategy is provided in the Supplementary Information.
