# Supplementary material for: Bora, CEP192 and Cenexin regulate distinct Plk1-dependent cell and centrosome cycle transitions
Source: Nat Commun. 2026 Jun 30;17:8158. doi: 10.1038/s41467-026-75033-5 (PMC13458439; doi:10.1038/s41467-026-75033-5)
Supplement: Supplementary file 4 [file 41467_2026_75033_MOESM39_ESM.pdf]

nature portfolio

Peer Review File

Bora, CEP192 and Cenexin regulate distinct Plk1-dependent cell and centrosome cycle transitions

Corresponding Author: Dr Devashish Dwivedi

This file contains all reviewer reports in order by version, followed by all author rebuttals in order by version.

Version 0:

Reviewer comments:

Reviewer #1

(Remarks to the Author)
The manuscript by Dwivedi et al. investigates how three Plk1 co-activators, i. e., Bora, Cep192, and Cenexin, activate distinct spatial pools of Plk1 to regulate different stages of the cell and centrosome cycles. Through live-cell FRET reporters, endogenously GFP-tagged Plk1, FRAP, Ultrastructure-Expansion Microscopy (U-ExM), PCNA dynamics, Separase sensors, and flow cytometry, the authors propose a model in which Bora predominantly activates cytoplasmic/nuclear Plk1 to drive mitotic entry and DNA-damage recovery, whereas Cep192 and Cenexin activate centrosomal Plk1 to regulate S-phase progression and centriole disengagement. Overall, the study is technically impressive and addresses an important biological question. The work has the potential to contribute significantly to understanding Plk1 regulation. However, a number of central claims require stronger experimental support before the manuscript can meet publication standards.
Major Suggestions:
1. Most key conclusions, including but not limited to Cep192 regulating S-phase origin firing, Bora promoting G2/damage recovery, Cep192/Cenexin controlling disengagement rely on single siRNA knockdowns. Rescue with RNAi-resistant constructs, or at minimum independent siRNAs producing consistent effects, is essential to exclude potential off-target contributions and to support mechanistic interpretations.
2. The FRET and FRAP data convincingly demonstrate changes in local activity and mobility, but there is no biochemical quantification of Plk1 T210 phosphorylation or Aurora-A/Plk1 complex formation is provided. Showing that Bora, Cep192, and Cenexin differentially regulate canonical Plk1 activation would substantiate the spatial activation model.
3. The finding that Cep192 regulates replication-origin firing, which is apparently independent of centrosomes, is interesting but it is mechanistically underdeveloped. Experiments using acute Aurora-A or Plk1 inhibition in S-phase could reveal if the role of Cep192 depends on these kinases.
4. Since Bora depletion causes strong G2 delays and global changes in Plk1 activity, the authors' observation of centriole disengagement could arise indirectly from altered timing. Including a rescue experiment (or additional timing controls) would help clarify if Bora, or its downstream pathways, directly regulates engagement status.
Minor Suggestions:
1. Clarify how FRET bleed-through and background were corrected, how ROIs were selected, and provide FRAP acquisition parameters (bleach pulse, ROI size, laser power). A brief sensor calibration, or dynamic-range control, would strengthen confidence in interpretation.
2. This manuscript should briefly mention positive control validation of the Separase sensor in the cell line to support claims that Separase is not activated during disengagement.

Reviewer #2

(Remarks to the Author)
This manuscript by Dwivedi and co-workers proposes that distinct pools of the PLK1 kinase control different functions of this kinase during the cell cycle in human cells. Bora, previously shown to act as an Aurora A activator targeting PLK1 for activation through phosphorylation, is identified as the main driver for functions of PLK1 in mitotic entry, recovery from DNA damage, and centrosome maturation. Two other PLK1 regulators, CEP192 and Cenexin, are instead identified for their functions in centriole disengagement, and limitedly to CEP192, S-phase progression through promotion of replication origin firing.

The mechanisms that regulate the ability of PLK1 to deliver its activity to different cellular locales and processes at the appropriate times remain incompletely understood. This manuscript represents an attempt to fill this gap. Building on previous work on the topic, the manuscript reports novel observations and reaches important new conclusions on the unique mechanisms of PLK1 regulation. Throughout the manuscript, the authors usually provide a fair and balanced account of previous work in this area, with small glitches that are discussed below. Overall, the manuscript succeeds in advancing our understanding of the function of PLK1, "sorting" PLK1 activity to different potential activators both in terms of localization and of activity. For instance, the authors positioned FRET based PLK1 activity sensors in the cytoplasm and nucleoplasm as well as at centrosomes, and monitored PLK1 activity in the G2 phase of the cell cycle when one or more of the three main activators studied here were targeted by RNAi. The authors also looked at the kinetics and mobility of PLK1 at centrosomes, revealing redundancies in the localization mechanism, but a stabilization of a specific centrosome pool of PLK1 by CEP192 and Cenexin that may be responsible for a highly significant fraction of the local activity. These experiments reveal the complexity of the PLK1 activation mechanism and the interrelation of activators. Exciting new observations reported here concern the role of PLK1 activators in the S- and G2-phases of the cell cycle, with CEP192 and Bora apparently taking the lead as PLK1 activators in these two phases of the cell cycle. The discovery of the involvement of CEP192 in origin firing is a major contribution of this study. The authors conclude that CEP192 in S-phase is important for activity at centrosomes and in the cytosol, but that centrosomes are not essential for the CEP192-dependent activation of PLK1 required for efficient DNA replication. Technically, I consider this to be a very well executed study, with experiments that are convincingly controlled. Thus, collectively, I am happy to express my strong support towards the publication of this study. I would like the authors to consider the following minor points for a possible improvement of the manuscript.

– It is certainly true that both Bora and CEP192 have the potential to "bridge" Aurora A and PLK1, and that this may be important for the activation of PLK1. However, Bora is the only protein that can promote PLK1 Thr210 phosphorylation, as far as I am aware of. Recent work of in vitro reconstitution now available as online preprints (https://doi.org/10.1101/2025.07.25.666769; https://doi.org/10.1101/2025.07.25.666770), cited here, has clarified the unique mechanism of Bora activation and also showed CEP192 seems to be unable to activate Aurora A towards PLK1 phosphorylation. I.e., the mechanisms of PLK1 activation by Bora and CEP192 appear to be fundamentally distinct. This is a topic that the manuscript may try to address with more clarity (verbally), as it seems to equate Bora and CEP192 in terms of molecular mechanism of PLK1 activation. I would like to urge the authors to clarify this point for their readership. As written, the only reference to this mechanism is at line 242

-Figure S1G – Why do Cyclin A2 levels decline when Aurora A is silenced?

-Maybe Figure 7 could be merged as the final panel of Figure 2?

-Line 262: Honestly, it is hard to conclude from this study that Cenexin is required at a specific cell cycle stage. Its contributions seem minor at best.

-Time profiles in Figure 8F could be drawn a bit more professionally. Also, by simply looking at the figure, it is unclear what "Processes we have identified" refers to.

Reviewer #3

(Remarks to the Author)
PLK1 is an essential kinase with various critical functions in cell cycle regulation including centrosome maturation, mitotic entry and DNA-damage response. PLK1 is localised to different structures through interaction with binding partners, mediated through its conserved polo-box domain. Additionally, PLK1 activity is modulated by numerous co-activators, including Bora, CEP192 and Cenexin. Whether and how the above functions of PLK1 are stratified between different co-activator-bound pools of the kinase has not been clearly established. In this manuscript, Meraldi and colleagues address this important question using a range of imaging techniques including FRET and expansion microscopy, concluding that there is indeed separation of function.

Firstly, they note that Bora is the primary driver behind PLK1 activity during mitotic entry and, as previously reported, recovery from DNA damage-induced cell cycle arrest. Unexpectedly, the authors find that centrosomal CEP192 is responsible for low levels of global PLK1 activity during S-phase with loss of CEP192 correlating with reducing levels of PCNA foci. Finally, they identify a contribution by both CEP192 and cenexin to PLK1-dependent centriole disengagement during late mitosis.

The manuscript is well-written, contains high quality data and focuses on an interesting problem that could appeal to a broad readership. However, in its current form, I am not convinced that the conclusions are fully supported by the data. Importantly, both the title and abstract state that Bora and CEP192 activate different Plk1 pools and regulate distinct cell and centrosome cycle transitions but the manuscript does not present direct experimental evidence to validate these claims. While some results suggest potential stratification of processes to different co-activators, on the whole the experimental approach lacks the resolution needed to differentiate the contributions of individual kinase pools.

Major Points:

1. The central claim of this manuscript is that functions of PLK1 are stratified across different pools of the kinase: "Our results indicate that the cytoplasmic and centrosomal pools of Plk1 activity are under distinct control….This indicates that both the

Plk1 pools must be considered separately." However, many of the results need better explanation as to how they fit this rationale. For example, from the FRAP experiments 2/3 of centrosomal PLK1 appears mobile; this mobile pool could therefore exchange between CEP192/cenexin at the centrosome and Bora in the cytoplasm. Indeed, this would explain why Bora has such a strong effect (>70%) on centrosome maturation. However, siCEP192 also dampened centrosome maturation by over 50%. Given these partial effects are observed for different interactors, why do the authors insist that these pools have non-overlapping functions and must be considered separately? An interdependence of these pools or perhaps Bora and CEP192 regulating different aspects of centrosome maturation and PLK1 function may be a more plausible interpretation. It would also be useful to know the relative size of PLK1 pools. For instance, if the pools interchange and the cytoplasmic pool is substantially larger than the centrosomal one, depletion of Bora would have an outsized effect on processes when compared to depletion of centrosomal interactors. Stronger evidence is required to claim separation of function, or the interpretation must be adjusted and claims toned down.

2. Line 263: "stage-specific regulation…." This is an interesting model, but aside from CEP192's role in S phase, the data does not align well with a linear state-specific regulation. For instance, in G2 cells, depletion of Bora or CEP192 both reduced PLK1 activity based on FRET assays, seemingly with additive effects. Like that of CEP192, depletion of Bora and cenexin also extended S phase duration. Furthermore even though depletion of Bora (but not the other co-factors) altered G2 duration, this effect was still significantly smaller than that seen with PLK1 inhibition, suggesting that additional co-activators might also play a role. Altogether these results argue against stage-specific regulation of PLK1 by individual co-factors, and instead highlight a role for additional co-activators and/or more complex regulatory interactions. Again, the authors should take into account these points when describing/discussing their data.

3. Aside from well documented off-target effects, siRNA depletion of these components could have consequences way beyond PLK1 regulation. For instance, it is not tested whether the increase in S-phase duration seen in CEP192-depleted cells is indeed due to drop in PLK1 activity. Although unlikely, it may be coincidental that CEP192 depletion and PLK1 inhibition exhibit a similar impact on PCNA levels. Have the authors tried to use multiple different CEP192 siRNA oligos to exclude off-target effects? As an extension, could the authors utilise Alpha-fold models and published data to design CEP192 mutants that do not interact with PLK1? Characterising these and the effects on PLK1 activity would help firm up this really exciting new finding.

4. A critical context sparsely touched throughout the manuscript is the mechanism of activation for each of these co-activators against PLK1. For instance, the authors point out that it remains to be seen whether CEP192-dependent PLK1 activity in S-phase requires Aurora-A. Given that this is the most original finding in the manuscript, the authors should address this. Having PLK1 activation at the centrosome prior to Aurora-A expression, potentially in a concentration-dependent manner would be a very interesting finding providing novel insight into PLK1 regulation. Assessing the timing and level of recruitment of Aurora-A to the centrosome relative to PLK1 would be important to explain these phenomena.

5. I am puzzled by the findings that CEP192-depleted cells do not enter mitosis in Fig 6 whether doxorubicin is added or not. This is quite an extreme phenotype, given that cells have 6+16 hours to complete S-phase, traverse through G2 and enter mitosis, whereas the average S-phase duration in these cells is ~10 hours based on Fig 3. Only around 1/8 of CEP192-depleted cells make it into mitosis in Fig. 6E, suggesting an inability to complete DNA replication in 22 hours. Is it possible that a significant percentage of CEP192-depleted cells get stuck in G2 and not S-phase? The observation is unexpected and potentially of great importance and therefore the authors should make more of an effort to figure out what is going on. For instance, does this depend on the type of G1/S arrest? Do cells ultimately recover? As before, have the authors observed this with multiple different CEP192-targeting siRNAs?

6. Please provide details of the FRET sensors in the main text and if space allows also include a diagram in the figure. Currently, readers need to check citations for details; as a minimum, please include that the sensor is based on c-jun and that PLK1 activity inversely correlates with FRET signal (as shown in MBoC paper referenced from 2018). Do the plots in Figure 1B and D correspond to inverse FRET ratios? Again, there is no mention of PACT in main text and in figure legend it is simply referred to as 'centrosomal Plk1 activity sensor'. Please include this version as well in diagram.

7. Figures are not particularly intuitive; treatments and cell cycle stages are not always shown in graphs or panels. For instance, in Figure 6 the labelling is identical for B,C and D,E, and without the figure legend it is impossible to know which conditions are depicted in which panel. Please indicate conditions/treatments for graphs and panels wherever is relevant.

8. Throughout, the presentation of statistical comparisons above graphs is somewhat messy and lacks clarity, addressing this will greatly improve the accessibility and ease of understanding of the manuscript. It may be advisable to remove the statistical comparisons not discussed in the text from the figures and then include these in the figure legend or a supplemental table. Also, the graphs are often crowded with p values (lots of 0.99), so the authors could remove some of the non-significant ones and replace these with ns or adding a statement to figure legends.

9. Methods need a much more through explanation of image quantitation. This was provided for expansion microscopy, separase activity and live cell imaging assays but nothing else. Particularly for FRAP analysis and calculation of mobile fraction of PLK1, this intuitively seems like the fraction of mobile PLK1 should always be less than 1 yet it is clear the mobile fraction can be >1 in many cases.

Minor Points

1. Figure 1D – Please show the points alongside these box and whisker plot, overlapping distributions are given strong

significance. For example, siControl vs siCEP192 has a p-value of less than <0.001. With well over 100 cells being analysed and each one statistically treated as an independent event, significance may be inflated.

2. Figure S1G is labelled with Cyclin A2, this should presumably be Aurora-A?

3. Line 41-42:" Nevertheless, a centrosome-bound pool has been reported to promote DNA replication15, 16". I followed the two references indicated here, wanting to compare your findings with the referenced literature, however neither of these papers appears to make reference to DNA replication, only recovery from DNA damage.

4. Line 82-83: Third, immunoblotting indicated that loss of one Plk1 co-activator is not compensated by overexpression of the other ones (Fig. S1E). From the provided blot (Figure S1E), CEP192 appears to increase slightly with cenexin depletion. Is thsi reproducible? Furthermore, the authors should highlight limitations such as changes in cell cycle distribution. For instance, Bora is degraded during mitosis and the author's work demonstrates that depleting the co-factors impacts S and G2 progression.

5. Figure S2D – The author's correctly state that PLK1 inhibition does not alter the relative proportion of mobile PLK1. However, it clearly alters the rate at which PLK1 is able to recover, this is worth noting.

6. Figure S2A-D: Figure legend notes N=3 while the figure itself states N=2.

7. For the live cell imaging of S and G2-phase progression in Figures 3 and 4, it would help the readers to include a brief description of what changes in your marker you use to mark the boundaries of each phase (e.g., loss of PCNA foci for G2 entry or change in nuclear shape for mitotic entry). This could go in main text or methods.

8. Figure 5E PLK1i – There appears to have been an image duplication of a FRET channel in place of Cyclin A2.

9. Figure 7, it would be interesting to see the levels of CEP192 at these centrosomes in the main conditions. This would confirm firstly whether any residual CEP192 persists at the centrosome in siCEP192 condition which would lead to a more intermediate phenotype. Additionally, given CEP192's γ-tubulin recruitment this could help identify whether the effect is at the level of reducing binding sites available for γ-tubulin or whether recruitment itself is perturbed.

10. Figure S6 is referred to as Figure S7 in the text on line 175.

11. Line 193-194: RPE1 CENEXIN-/- knock-out cells (to avoid applying a technically challenging triple siRNA treatment). Why only use this knock out for the imaging? The authors have been successfully using triple depletion throughout the manuscript, confirmed by western blotting in Figure S1G. This makes the rationale provided somewhat obsolete and the experiment is then inconsistent with the bulk of experimental work within the paper.

12. Line 196-197: "We conclude that centriole disengagement is primarily driven by the centrosomal Plk1 activators Cep192 and Cenexin," Bora depletion causes ~90% of centrioles to be disengaged, which is reduced (albeit not entirely) by co-depletion or knockout of CEP192 and cenexin. The authors conclude that the effects of Bora depletion are at least partially manifested through CEP192 and Cenexin. While the above statement is correct, this needs a much clearer explanation especially to non-experts given the highly penetrant effect of Bora depletion alone.

13. Line 270: "This differential regulatory network can… where the mitotic progression is delayed while the centrosome cycle advances, causing a severe desynchrony between the two otherwise synchronous processes." Is this regarding the increase premature centriole disengagement the authors observed? If so, it may be worth clarifying the statement as authors also demonstrated that during G2, centrosome maturation was also impacted by Bora depletion.

Reviewer #4

(Remarks to the Author)
I co-reviewed this manuscript with one of the reviewers who provided the listed reports. This is part of the Nature Communications initiative to facilitate training in peer review and to provide appropriate recognition for Early Career Researchers who co-review manuscripts.

Version 1:

Reviewer comments:

Reviewer #2

(Remarks to the Author)
I thank the authors for their careful consideration of the reviewers' comments. I feel that they have greatly improved the clarity and scope of the manuscript, and I am very happy to support publication.

Reviewer #3

(Remarks to the Author)

The authors have done an excellent job addressing my comments both in terms of adding important new data (e.g., PLK1 binding mutants) and improving presentation and clarity of their manuscript. I am fully supportive of publication.

Reviewer #4

(Remarks to the Author)
I co-reviewed this manuscript with one of the reviewers who provided the listed reports. This is part of the Nature Communications initiative to facilitate training in peer review and to provide appropriate recognition for Early Career Researchers who co-review manuscripts.

Open Access This Peer Review File is licensed under a Creative Commons Attribution 4.0 International License, which permits use, sharing, adaptation, distribution and reproduction in any medium or format, as long as you give appropriate credit to the original author(s) and the source, provide a link to the Creative Commons license, and indicate if changes were made.
In cases where reviewers are anonymous, credit should be given to 'Anonymous Referee' and the source.
The images or other third party material in this Peer Review File are included in the article's Creative Commons license, unless indicated otherwise in a credit line to the material. If material is not included in the article's Creative Commons license and your intended use is not permitted by statutory regulation or exceeds the permitted use, you will need to obtain permission directly from the copyright holder.
To view a copy of this license, visit https://creativecommons.org/licenses/by/4.0/

<u>Point-by point rebuttal</u>

We thank all three reviewers for the constructive input. We have addressed their concerns in the following manner.

Reviewer #1:

The manuscript by Dwivedi et al. investigates how three Plk1 co-activators, i. e., Bora, Cep192, and Cenexin, activate distinct spatial pools of Plk1 to regulate different stages of the cell and centrosome cycles. Through live-cell FRET reporters, endogenously GFP-tagged Plk1, FRAP, Ultrastructure-Expansion Microscopy (U-ExM), PCNA dynamics, Separase sensors, and flow cytometry, the authors propose a model in which Bora predominantly activates cytoplasmic/nuclear Plk1 to drive mitotic entry and DNA-damage recovery, whereas Cep192 and Cenexin activate centrosomal Plk1 to regulate S-phase progression and centriole disengagement. Overall, the study is technically impressive and addresses an important biological question. The work has the potential to contribute significantly to understanding Plk1 regulation. However, a number of central claims require stronger experimental support before the manuscript can meet publication standards.

We thank the reviewer for the positive comments and have addressed the concerns in the following way:

Major Suggestions:

1. **Most key conclusions, including but not limited to Cep192 regulating S-phase origin firing, Bora promoting G2/damage recovery, Cep192/Cenexin controlling disengagement rely on single siRNA knockdowns. Rescue with RNAi-resistant constructs, or at minimum independent siRNAs producing consistent effects, is essential to exclude potential off-target contributions and to support mechanistic interpretations.**

   As suggested, we carried out siRNA rescue experiments to validate our key conclusions. This includes the role of Bora in regulating DNA damage checkpoint recovery and centrosome maturation, and in the case of Cep192 its ability to regulate S phase progression and centrosome maturation. We generated stable RPE1 cell lines expressing RNAi-resistant Bora (WT) or Cep192 (WT and a Plk1 binding defective mutant). RNAi-resistant Bora WT efficiently rescued checkpoint recovery (Fig. 6D-E) and centrosome maturation (Fig. 7D-F). Similarly, reintroducing Cep192 WT but not a Plk1 binding defective mutant (Cep192 T44A, S995A), restored the normal percentage of S-phase cells (Fig 4D) and levels of Pericentrin and γ-tubulin on mitotic centrosomes (Fig. 7G-I). These experiments thus fully support our conclusions. In the case of Cenexin, we only see its implication during centriole disengagement, and this result is based on a Cenexin knock-out cell line.

2. **The FRET and FRAP data convincingly demonstrate changes in local activity and mobility, but there is no biochemical quantification of Plk1 T210 phosphorylation or Aurora-A/Plk1 complex formation is provided. Showing that Bora, Cep192, and Cenexin differentially regulate canonical Plk1 activation would substantiate the spatial activation model.**

   As suggested, we complemented our Plk1 activity FRET measurements after Bora, Cep192 or Cenexin depletion with quantitative immunofluorescence measurements of Plk1 T210 phosphorylation (Fig. 1F-I). We found that both methods yielded qualitatively equivalent results, showing that Plk1 T210 phosphorylation was primarily driven by Aurora-A-Bora in G2 and Aurora-A-Cep192 in S-phase.

3. **The finding that Cep192 regulates replication-origin firing, which is apparently independent of centrosomes, is interesting but it is mechanistically underdeveloped. Experiments using acute Aurora-A or Plk1 inhibition in S-phase could reveal if the role of Cep192 depends on these kinases**.

   Based on the reviewer input, we have now strongly expanded this section of the manuscript. First, we show that RNAi-resistant WT Cep192 complements the S-phase impairment seen after Cep192 RNAi, but that a Cep192 mutant defective in Plk1 binding cannot (Fig 4D). This confirms our hypothesis that Cep192 regulates S-phase via Plk1. Moreover, our new results now demonstrate S-phase entry, replication origin firing and S-phase progression are under the control of the Aurora-A-Cep192-Plk1 axis. Indeed, acute Aurora-A inhibition, Plk1 inhibition or Cep192 depletion delay S-Phase entry to the same extent after a Cdk4/6 inhibition release (Fig. 5E-H). We also found that Aurora-A depletion prolongs the duration of S-phase by similar magnitude than Plk1 or Cep192 depletion (Fig. 5A and B vs. Fig. 3C). Furthermore, inhibition of Plk1 or Aurora A (Fig. 5I and J), or Cep192 depletion (Fig. 3F and G) suppressed the formation of active replication forks (visualised as number of PCNA foci per nucleus) to the same extent, consistent with a linear pathway. Moreover, our live imaging experiments with endogenously tagged Aurora-A or Plk1 show that Aurora A is recruited on the centrosomes prior to Plk1 (Fig. 5O-Q). Finally, Aurora Ai abrogated both cyto/nucleoplasmic and centrosomal Plk1 activity in S phase cells (Fig. 5L-N), and induction of a constitutively active Plk1 mimicking Aurora-A phosphorylation, but not the catalytically inactive Plk1 mutant, rescued the number of PCNA foci in Aurora-A inhibited cells (Fig. 5R-T). These results therefore lead us to propose that phosphorylation of Plk1 by a Cep192-Aurora-A complex on centrosomes in S-phase is rate-limiting for S-phase entry and replication origin firing.

4. **Since Bora depletion causes strong G2 delays and global changes in Plk1 activity, the authors 'observation of centriole disengagement could arise indirectly from altered timing. Including a rescue experiment (or additional timing controls) would help clarify if Bora, or its downstream pathways, directly regulates engagement status.**

   As suggested, we now included two additional experiments to better distinguish how Bora depletion induces centriole disengagement in G2. Indeed, since Bora depletion also prolongs G2 phase and impair centrosome maturation, premature centriole disengagement could represent a downstream consequence of these two events. First, we tested whether impaired centrosome maturation, which we mimicked by Pericentrin depletion, would induce premature centriole dis-engagement, and found this not to be the case (Figure 8E-F). Second, we show that depletion of Cyclin A2, which leads to a similar long G2 delay, does not suffice on its own to induce premature centriole disengagement (Figure 8G and H). This is consistent with our previous studies, which showed that inhibition of Cdk1 or Plk1, which both also induce a strong G2 delay, did not cause premature centriole disengagement (Wilhelm et al., Nat. Commun., 2019 and Dwivedi et al., Nat Commun., 2023). Nevertheless, we also note that our previous study showed that the Plk1-dependent premature centriole disengagement induced by mild replication stress, could be suppressed by forcing cells to rapidly enter mitosis with a Wee1 inhibitor. Given that centriole disengagement in Bora-depleted cells depends on the two other Plk1 activiator we overall conclude that centriole disengagement in Bora depleted cells (1) occurs independently of centrosome maturation, and (2) is the result of a sustained Plk1 activity at centrosomes, combined with reduced cyto/nucleoplasmic Plk1 activity in a prolonged G2 phase (see revised discussion page 14).

Minor Suggestions:

1. **Clarify how FRET bleed-through and background were corrected, how ROIs were selected, and provide FRAP acquisition parameters (bleach pulse, ROI size, laser power). A brief sensor calibration, or dynamic-range control, would strengthen confidence in interpretation.**

   As suggested, we have now included all the details in the methods section of the revised manuscript. Our revised manuscript now includes "FRET analysis was performed in ImageJ. Background fluorescence was subtracted independently from both CFP and YFP channels …… confirming responsiveness within the experimental range." Described on pages 20/21. We also added an entire new section on FRAP "**FRAP analysis determining mobile Plk1 fraction ……..** intensity at the plateau, respectively." on page 24.

2. **This manuscript should briefly mention positive control validation of the Separase sensor in the cell line to support claims that Separase is not activated during disengagement.**

   As suggested, we now include mild replication stress (400 nM Aphidicolin) as positive control in Figure 8I and J, as we had previously shown that such a configuration results in a detectable Separase activity at centrosome concomitant with a Separase-dependent premature centriole disengagement (Dwivedi et al., Nat Commun., 2023).

Reviewer #2:

This manuscript by Dwivedi and co-workers proposes that distinct pools of the PLK1 kinase control different functions of this kinase during the cell cycle in human cells. Bora, previously shown to act as an Aurora A activator targeting PLK1 for activation through phosphorylation, is identified as the main driver for functions of PLK1 in mitotic entry, recovery from DNA damage, and centrosome maturation. Two other PLK1 regulators, CEP192 and Cenexin, are instead identified for their functions in centriole disengagement, and limitedly to CEP192, S-phase progression through promotion of replication origin firing.

The mechanisms that regulate the ability of PLK1 to deliver its activity to different cellular locales and processes at the appropriate times remain incompletely understood. This manuscript represents an attempt to fill this gap. Building on previous work on the topic, the manuscript reports novel observations and reaches important new conclusions on the unique mechanisms of PLK1 regulation. Throughout the manuscript, the authors usually provide a fair and balanced account of previous work in this area, with small glitches that are discussed below. Overall, the manuscript succeeds in advancing our understanding of the function of PLK1, "sorting" PLK1 activity to different potential activators both in terms of localization and of activity. For instance, the authors positioned FRET based PLK1 activity sensors in the cytoplasm and nucleoplasm as well as at centrosomes, and monitored PLK1 activity in the G2 phase of the cell cycle when one or more of the three main activators studied here were targeted by RNAi. The authors also looked at the kinetics and mobility of PLK1 at centrosomes, revealing redundancies in the localization mechanism, but a stabilization of a specific centrosome pool of PLK1 by CEP192 and Cenexin that may be responsible for a highly significant fraction of the local activity. These experiments reveal the complexity of the PLK1 activation mechanism and the interrelation of activators. Exciting new observations reported here concern the role of PLK1 activators in the S- and G2-phases of the cell cycle, with CEP192 and Bora apparently taking the lead as PLK1 activators in these two phases of the cell cycle. The discovery of the involvement of CEP192 in origin firing is a major contribution of this study. The authors conclude that CEP192 in S-phase is important for activity at centrosomes and in the cytosol, but that centrosomes are not essential for the CEP192-dependent activation of PLK1 required for efficient DNA replication. Technically, I consider this to be a very well executed study, with experiments that are convincingly controlled. Thus, collectively, I am happy to express my strong support towards the publication of this study. I would like the authors to consider the following minor points for a possible improvement of the manuscript.

We thank the reviewer for the positive comments and have addressed the concerns in the following way:

1. **It is certainly true that both Bora and CEP192 have the potential to "bridge" Aurora A and PLK1, and that this may be important for the activation of PLK1. However, Bora is the only protein that can promote PLK1 Thr210 phosphorylation, as far as I am aware of. Recent work of in vitro reconstitution now available as online preprints (https://doi.org/10.1101/2025.07.25.666769; https://doi.org/10.1101/2025.07.25.666770), cited here, has clarified the unique mechanism of Bora activation and also showed CEP192 seems to be unable to activate Aurora A towards PLK1 phosphorylation. I.e., the mechanisms of PLK1 activation by Bora and CEP192 appear to be fundamentally distinct. This is a topic that the manuscript may try to address with more clarity (verbally), as it seems to equate Bora and CEP192 in terms of molecular mechanism of PLK1 activation. I would like to urge the authors to clarify this point for their readership. As written, the only reference to this mechanism is at line 242**

   We thank the reviewer for suggesting a more thorough discussion and investigation of the activation mechanism of Plk1. Our new quantification of Plk1 T210 phosphorylation in cellulo agrees with the two cited publications, as it indicates that in G2 T210 phosphorylation is primarily Bora-dependent (Fig. 1F and G). Indeed, Esposito-Verza et al., 2026 specifically shows that in vitro the Aurora-A-Cep192 complex is inefficient at phosphorylating Plk1 on T210. Nevertheless, our new in cellulo results in Fig. S4, studying the activation of Plk1 in S-phase, show that in S-phase a weak but critical amount of Plk1 T210 depends on Aurora-A and Cep192, but not on Bora (Fig S4C and D). We therefore propose that the Aurora-A-Cep192 complex can phosphorylate Plk1 in the specific context of S-phase (see revised discussion on page 13). We envisage two possibilities that could explain this result, which we present in the discussion. A first possibility, is that Aurora-A-Cep192 could phosphorylate Plk1 with a low but sufficient efficacy in S-phase, before being displaced by Bora as cells enter G2, consistent with the observation that Bora and Cep192 compete for the same binding site on Aurora-A. A second possibility is that the Cep192-Aurora-A complex may be supported in S-phase in activating Plk1 by an additional interactor present at centrosomes. This interactor would still present in acentriolar cells and would explain how Plk1 activation is still Cep192-dependent in our acentriolar cells. In this case Cep192 would still be displaced from Aurora-A in G2, when Bora levels increase (Seki et al., Science, 2008)

2. **Figure S1G – Why do Cyclin A2 levels decline when Aurora A is silenced?**

   We apologise for this labelling mistake in the western blot confirming Aurora A depletion. We have now corrected it in the revised version and thank the reviewer.

3. **Maybe Figure 7 could be merged as the final panel of Figure 2?**

   We thank the reviewer for the suggestion but given the addition of novel elements in both figures, we feel that the two figures should remain separated.

4. **Line 262: Honestly, it is hard to conclude from this study that Cenexin is required at a specific cell cycle stage. Its contributions seem minor at best.**

   We thank the reviewer for this comment. We now state that the contribution of Cenexin is indeed minor and may only become rate-limiting in the context of centriole dis-engagement, when Bora, the main mitotic activator of Plk1 has been degraded. We now also state in the discussion As such, "Cenexin might not be a cell cycle stage specific Plk1 activator but might more provide a localized Plk1 activity".

5. **Time profiles in Figure 8F could be drawn a bit more professionally. Also, by simply looking at the figure, it is unclear what "Processes we have identified" refers to.**

   We thank the reviewer the for the comment, we have now adjusted the final model.

Reviewer #3:

PLK1 is an essential kinase with various critical functions in cell cycle regulation including centrosome maturation, mitotic entry and DNA-damage response. PLK1 is localised to different structures through interaction with binding partners, mediated through its conserved polo-box domain. Additionally, PLK1 activity is modulated by numerous co-activators, including Bora, CEP192 and Cenexin. Whether and how the above functions of PLK1 are stratified between different co-activator-bound pools of the kinase has not been clearly established. In this manuscript, Meraldi and colleagues address this important question using a range of imaging techniques including FRET and expansion microscopy, concluding that there is indeed separation of function.

Firstly, they note that Bora is the primary driver behind PLK1 activity during mitotic entry and, as previously reported, recovery from DNA damage-induced cell cycle arrest. Unexpectedly, the authors find that centrosomal CEP192 is responsible for low levels of global PLK1 activity during S-phase with loss of CEP192 correlating with reducing levels of PCNA foci. Finally, they identify a contribution by both CEP192 and cenexin to PLK1-dependent centriole disengagement during late mitosis.

The manuscript is well-written, contains high quality data and focuses on an interesting problem that could appeal to a broad readership. However, in its current form, I am not convinced that the conclusions are fully supported by the data. Importantly, both the title and abstract state that Bora and CEP192 activate different Plk1 pools and regulate distinct cell and centrosome cycle transitions but the manuscript does not present direct experimental evidence to validate these claims. While some results suggest potential stratification of processes to different co-activators, on the whole the experimental approach lacks the resolution needed to differentiate the contributions of individual kinase pools.

We thank the reviewer for the positive comments and have addressed the concerns in the following manner:

Major Points:

1. **The central claim of this manuscript is that functions of PLK1 are stratified across different pools of the kinase: "Our results indicate that the cytoplasmic and centrosomal pools of Plk1 activity are under distinct control….This indicates that both the Plk1 pools must be considered separately." However, many of the results need better explanation as to how they fit this rationale. For example, from the FRAP experiments 2/3 of centrosomal PLK1 appears mobile; this mobile pool could therefore exchange between CEP192/cenexin at the centrosome and Bora in the cytoplasm. Indeed, this would explain why Bora has such a**

strong effect (>70%) on centrosome maturation. However, siCEP192 also dampened centrosome maturation by over 50%. Given these partial effects are observed for different interactors, why do the authors insist that these pools have non-overlapping functions and must be considered separately? An interdependence of these pools or perhaps Bora and CEP192 regulating different aspects of centrosome maturation and PLK1 function may be a more plausible interpretation. It would also be useful to know the relative size of PLK1 pools. For instance, if the pools interchange and the cytoplasmic pool is substantially larger than the centrosomal one, depletion of Bora would have an outsized effect on processes when compared to depletion of centrosomal interactors. Stronger evidence is required to claim separation of function, or the interpretation must be adjusted and claims toned down.

We thank the reviewer for the comment, which helped us to re-interpret our results. First, as suggested by the reviewer we quantified the respective Plk1 levels in the cyto/nucleoplasm and centrosomes, comparing by immunoblotting whole cell lysates and isolated centrosomes from KE37 cells. Our results, which now put the two populations in a better perspective, indicate that in this cell line 1-2% of Plk1 is present at centrosomes (which corresponds to a 10-20 fold enrichment, given that centrosomes occupy roughly 1/1000 of the cell volume; Fig. 2A). This helps to explain how Bora depletion can have a large effect on cytoplasmic Plk1, while not affecting much the smaller centrosomal activity, which can rely on Cep192 and Cenexin. As we now explain in our extensively revised discussion, overall, our results suggest a large interchangeable fraction combined with small restricted Plk1 pool. We therefore adapted our title, abstract and discussion to reflect these more contrasted results. This allows us also to emphasize more in the title and the abstract what we believe is the true novelty of the manuscript, how different Plk1 activators drive distinct cell- and centrosome cycle steps in S (Cep192) and G2 (Bora). Indeed, this distinction that was strongly reinforced by our detailed mechanistic investigation of the role of Cep192, Aurora-A and Plk1 in driving S-phase progression.

2. **Line 263: "stage-specific regulation…." This is an interesting model, but aside from CEP192's role in S phase, the data does not align well with a linear state-specific regulation. For instance, in G2 cells, depletion of Bora or CEP192 both reduced PLK1 activity based on FRET assays, seemingly with additive effects. Like that of CEP192, depletion of Bora and cenexin also extended S phase duration. Furthermore even though depletion of Bora (but not the other co-factors) altered G2 duration, this effect was still significantly smaller than that seen with PLK1 inhibition, suggesting that additional co-activators might also play a role. Altogether these results argue against stage-specific regulation of PLK1 by individual**

co-factors, and instead highlight a role for additional co-activators and/or more complex regulatory interactions. Again, the authors should take into account these points when describing/discussing their data.

Here, we partially disagree with the reviewer, as our old and novel data clearly indicate that the small activity of Plk1 in S-phase is primarily driven by Cep192, while the G2-related activities of Plk1, such as mitotic entry and centrosome maturation are most dependent on Bora (Fig. 4- 7). This does not mean that it only depends on Bora, as we agree with the reviewer that Cep192 also contributes to the Plk1-dependent processes in G2 (although we note that Cep192 has a Plk1-independent contribution to centrosome maturation), as illustrated on our final model in Figure 9. Moreover, our data do not point to yet additional Plk1 activators in G2, since loss of Bora, Cep192 and Cenexin reduces Plk1 activity to the same extent as Plk1 inhibition (Fig. 1B-E and Fig. S1A-D). Overall, this suggests that, as cells transition from S-phase to G2, Plk1 switches from Cep192 as a main activator to Bora, consistent with recent structural data showing that the two proteins compete for the same binding site on Aurora-A, the main activator of Plk1 (Pilan et. al., EMBO J., 2026)). We now make this point more explicit in the discussion to explain our characterization of stage-specific regulation

3. **Aside from well documented off-target effects, siRNA depletion of these components could have consequences way beyond PLK1 regulation. For instance, it is not tested whether the increase in S-phase duration seen in CEP192-depleted cells is indeed due to drop in PLK1 activity. Although unlikely, it may be coincidental that CEP192 depletion and PLK1 inhibition exhibit a similar impact on PCNA levels. Have the authors tried to use multiple different CEP192 siRNA oligos to exclude off-target effects? As an extension, could the authors utilise Alpha-fold models and published data to design CEP192 mutants that do not interact with PLK1? Characterising these and the effects on PLK1 activity would help firm up this really exciting new finding.**

We thank the reviewer for raising this important point regarding potential off-target effects and the specificity of the Cep192 depletion phenotype. To address this, we performed rescue experiments, in which expression of either an RNAi-resistant Cep192 WT or a Plk1-binding defective CEP192 mutant (2A) (Meng et al., (2015)) was induced in Cep192-depleted cells. While the reintroduction of Cep192 WT fully rescued the S phase defects, the Cep192 2A mutant did not, indicating that observed S-phase defects are specific to Cep192 depletion and linked to the regulation of Plk1 activity by Cep192 (Fig. 4D). This conclusion is further supported by the fact that RNAi mediated depletion or pharmacological inhibition of Plk1 phenocopies the increase in

S-phase duration and the reduction in PCNA levels seen after Cep192 depletion, and the fact that Cep192 depletion leads to a measurable decrease in centrosomal Plk1 activity (Fig. 3B-C and Fig. 5G-H).

4. **A critical context sparsely touched throughout the manuscript is the mechanism of activation for each of these co-activators against PLK1. For instance, the authors point out that it remains to be seen whether CEP192-dependent PLK1 activity in S-phase requires Aurora-A. Given that this is the most original finding in the manuscript, the authors should address this. Having PLK1 activation at the centrosome prior to Aurora-A expression, potentially in a concentration-dependent manner would be a very interesting finding providing novel insight into PLK1 regulation. Assessing the timing and level of recruitment of Aurora-A to the centrosome relative to PLK1 would be important to explain these phenomena.**

   We thank the reviewer for highlighting this important mechanistic aspect of PLK1 activation. We agree that understanding the relationship between CEP192-dependent PLK1 activity and Aurora A is critical, particularly in the context of our S-phase findings. We now show in our revised manuscript that Plk1-activation in S-phase indeed depends both on Aurora-A and Cep192 (Fig. 4D and 5K-N). We demonstrate that Aurora A depletion leads to the same increase in S phase duration as after Plk1 depletion (Fig. 5A and B), and that in cells synchronised in G1, acute Aurora inhibition resulted in the same delayed S-phase entry as seen after Plk1 inhibition (Fig. 5E and F). Second, our live-cell imaging experiments with endogenously tagged proteins confirm that Aurora A is recruited on to the centrosomes prior to Plk1 (Fig. 5 O-Q) and that acute Aurora A inhibition in S phase results in loss of Plk1 activity and reduction in number of PCNA foci per nuclei (proxy for the number of DNA replication sites), similar to inhibition of Plk1 and Cep192 depletion (Fig. 5I-N). Finally, when we expressed constitutively active or catalytically inactive Plk1 mutants in cells treated with Aurora-A inhibitor, we found that constitutively active Plk1 but not the catalytically inactive mutant efficiently rescued the replication origin firing (Fig. 5R-T), indicating that Aurora-A acts upstream of Plk1.

5. **I am puzzled by the findings that CEP192-depleted cells do not enter mitosis in Fig 6 whether doxorubicin is added or not. This is quite an extreme phenotype, given that cells have 6+16 hours to complete S-phase, traverse through G2 and enter mitosis, whereas the average S-phase duration in these cells is ~10 hours based on Fig 3. Only around 1/8 of CEP192-depleted cells make it into mitosis in Fig. 6E, suggesting an inability to complete DNA**

replication in 22 hours. Is it possible that a significant percentage of CEP192-depleted cells get stuck in G2 and not S-phase? The observation is unexpected and potentially of great importance and therefore the authors should make more of an effort to figure out what is going on. For instance, does this depend on the type of G1/S arrest? Do cells ultimately recover? As before, have the authors observed this with multiple different CEP192-targeting siRNAs?

We thank the reviewer for carefully analysing this point and for highlighting the apparent discrepancy between S-phase duration and mitotic entry in CEP192-depleted cells. A key aspect that was not sufficiently clarified in the original manuscript is that release from CDK4/6 inhibition does not result in immediate S-phase entry. Cells typically initiate S-phase ~6 hours after release. Importantly, CEP192 depletion significantly delays both S-phase onset and progression. Based on our measurements, we find that **control cells** spend ~6.5 hours till S-phase entry (as seen by the appearance of PCNA foci), ~4 hours in S-phase, ~4.5 hours in G2. Therefore, cells are expected to enter mitosis 15 hours after release from Palbociclib. **CEP192-depleted cells** in contrast need ~11.5 hours to enter S-phase, spend ~10 hours in S-phase and ~6 hours in G2. They therefore need ~27.5 hours to enter mitosis. Thus, within the 22-hour observation window used in Fig. 6, a large fraction of CEP192-depleted cells are unlikely to reach mitosis. This explains why only a minority (~1/8) of cells entered mitosis during this time frame, without invoking a complete failure of DNA replication. Regarding the reviewer's suggestion that cells may accumulate in G2, our data indicate that while there is a modest extension of G2 duration, the predominant delay arises from the delayed S-phase entry and progression (Fig 3-4). We agree that this is an important point and have now clarified these timing relationships explicitly in the revised manuscript (Page 9). In addition, to address robustness, we have now shown that this accumulation in S phase is rescued by reintroducing RNAi-resistant Cep192 WT in Cep192 depleted cells, arguing against off-target effects. Finally, we agree that the fate of CEP192-depleted cells beyond the imaging window is an interesting question but feel that it is beyond the scope of the present study, which focuses much more on the novel S-phase role of Cep192.

6. **Please provide details of the FRET sensors in the main text and if space allows also include a diagram in the figure. Currently, readers need to check citations for details; as a minimum, please include that the sensor is based on c-jun and that PLK1 activity inversely correlates with FRET signal (as shown in MBoC paper referenced from 2018). Do the plots in Figure 1B and D correspond to inverse FRET ratios? Again, there is no mention of PACT in main**

text and in figure legend it is simply referred to as 'centrosomal Plk1 activity sensor'. Please include this version as well in diagram.

We thank the reviewer the comment, we have now included additional details of both the cytosolic and centrosome-targeted FRET biosensor in the figure (Fig. 1A) and expanded their description in the methods section. Specifically, we now show in the figure that the sensor is based on a c-Jun–derived phosphorylation motif that is phosphorylated by Plk1, resulting in a conformational change that alters FRET efficiency. We also clarify that Plk1 activity inversely correlates with the measured FRET signal. Accordingly, the FRET measurement plots shown throughout the manuscript represent inverse FRET ratios, which we chose to display to facilitate intuitive interpretation of increased kinase activity. In addition, we have clarified the centrosome-targeting strategy of the sensor. The centrosomal version contains the PACT domain, which directs the sensor to centrosomes. This information is now explicitly included in both the main text and the figure legend (Fig. 1).

7. **Figures are not particularly intuitive; treatments and cell cycle stages are not always shown in graphs or panels. For instance, in Figure 6 the labelling is identical for B,C and D,E, and without the figure legend it is impossible to know which conditions are depicted in which panel. Please indicate conditions/treatments for graphs and panels wherever is relevant.**

   We thank the reviewer the comment, we have now made changes to the figure to better depict the difference between the otherwise same looking experiments.

8. **Throughout, the presentation of statistical comparisons above graphs is somewhat messy and lacks clarity, addressing this will greatly improve the accessibility and ease of understanding of the manuscript. It may be advisable to remove the statistical comparisons not discussed in the text from the figures and then include these in the figure legend or a supplemental table. Also, the graphs are often crowded with p values (lots of 0.99), so the authors could remove some of the non-significant ones and replace these with ns or adding a statement to figure legends**.

   We thank for the comment and have now introduced these changes as suggested by the reviewer.

9. **Methods need a much more through explanation of image quantitation. This was provided for expansion microscopy, separase activity and live cell imaging assays but nothing else.**

Particularly for FRAP analysis and calculation of mobile fraction of PLK1, this intuitively seems like the fraction of mobile PLK1 should always be less than 1 yet it is clear the mobile fraction can be >1 in many cases.

10. We thank the reviewer for highlighting the need for clarification of the FRAP analysis. We agree that, conceptually, the mobile fraction is expected to be ≤1. In our analysis, however, the mobile fraction is calculated based on normalized fluorescence intensities derived from live-cell imaging, and values slightly exceeding 1 can arise due to experimental and analytical variability. Specifically, these deviations can result from factors such as noise in intensity measurements, small fluctuations in pre-bleach normalization, background subtraction, and minor cell or focal drift during acquisition. In addition, variability in bleaching efficiency and local fluorescence fluctuations at centrosomes can further influence recovery curves. As the calculation relies on relative fluorescence intensities rather than absolute molecular counts, such effects can occasionally lead to apparent mobile fractions >1. Importantly, these instances are infrequent and do not affect the overall trends or comparative conclusions drawn between conditions. We have now clarified the calculation method, normalization procedure, and these limitations in the revised Methods section to improve transparency. (Page 24-25)

Minor Points

1. **Figure 1D – Please show the points alongside these box and whisker plot, overlapping distributions are given strong significance. For example, siControl vs siCEP192 has a p-value of less than <0.001. With well over 100 cells being analysed and each one statistically treated as an independent event, significance may be inflated.**

We thank the reviewer the comment. We agree that treating individual cells as independent observations can inflate statistical significance, particularly in large datasets where subtle distributional overlaps may still yield very low p-values. We now overlay individual data points on the box-and-whisker plots, allowing the reader to directly assess the degree of overlap between distributions rather than relying solely on summary statistics. In addition, for all our immunofluorescence experiments, we have re-analyzed the data by treating each biological replicate (experiment) as an independent observation. Specifically, mean values from each experiment were calculated and used for statistical testing. The corresponding p-values based on these independent experimental means are now reported alongside the distributions derived from single-cell analyses. These changes provide a more conservative and statistically robust

assessment of the observed differences and have been incorporated into the revised figures and manuscript text.

2. **Figure S1G is labelled with Cyclin A2, this should presumably be Aurora-A?**

   We thank the reviewer the comment, we apologise for this labelling mistake. We have now corrected it in the revised version.

3. **Line 41-42:" Nevertheless, a centrosome-bound pool has been reported to promote DNA replication15, 16". I followed the two references indicated here, wanting to compare your findings with the referenced literature, however neither of these papers appears to make reference to DNA replication, only recovery from DNA damage.**

   We thank the reviewer the comment, we have now replaced them with the correct reference (Shen et al., Cell Res., 2013).

4. **Line 82-83: Third, immunoblotting indicated that loss of one Plk1 co-activator is not compensated by overexpression of the other ones (Fig. S1E). From the provided blot (Figure S1E), CEP192 appears to increase slightly with Cenexin depletion. Is this reproducible? Furthermore, the authors should highlight limitations such as changes in cell cycle distribution. For instance, Bora is degraded during mitosis and the author's work demonstrates that depleting the co-factors impacts S and G2 progression.**

   We thank the reviewer the comment, we tested the levels of Cep192 after Cenexin depletion and did not find any significant upregulation/increased recruitment of Cep192 on the centrosomes (Fig S1H-I). We agree, however, that interpretation of total cell immunoblotting has inherent limitations in this context. In particular, changes in cell cycle distribution can influence protein abundance measurements, especially for cell cycle–regulated proteins such as Bora, which is degraded during mitosis. To point to this limitation, we added the following note in the figure legend: "*Note that in a first approximation, depletion of one Plk1 activator did not change the expression of the other activators; our data does however not exclude more minor changes due to changes in cell cycle phase durations.*"

5. **Figure S2D – The author's correctly state that PLK1 inhibition does not alter the relative proportion of mobile PLK1. However, it clearly alters the rate at which PLK1 is able to recover, this is worth noting.**

   We thank the reviewer the comment, we have now also added this to our results and explicitly state "This change in the mobility was due to a change in Plk1 anchoring rather than Plk1 activity itself, since Plk1 inhibition had no effect on the relative proportion of mobile and immobile Plk1, albeit it affected the recovery kinetics".

6. **Figure S2A-D: Figure legend notes N=3 while the figure itself states N=2.**

   We thank the reviewer the comment, we apologise for this labelling mistake that occurred during the assembly of figures. we have now corrected it in the revised version.

7. **For the live cell imaging of S and G2-phase progression in Figures 3 and 4, it would help the readers to include a brief description of what changes in your marker you use to mark the boundaries of each phase (e.g., loss of PCNA foci for G2 entry or change in nuclear shape for mitotic entry). This could go in main text or methods.**

   We thank the reviewer the comment, we have now included the schematic in the main figure (Fig. 3A) and included the description of changes in the nuclear localisation of PCNA which we utilise as marker for G1/S, S/G2 and G2/M transitions in methods section.

8. **Figure 5E PLK1i – There appears to have been an image duplication of a FRET channel in place of Cyclin A2.**

   We thank the reviewer the comment, we apologise for this labelling mistake that occurred during the assembly of figures. we have now corrected it in the revised version.

9. **Figure 7, it would be interesting to see the levels of CEP192 at these centrosomes in the main conditions. This would confirm firstly whether any residual CEP192 persists at the centrosome in siCEP192 condition which would lead to a more intermediate phenotype. Additionally, given CEP192's γ-tubulin recruitment this could help identify whether the effect is at the level of reducing binding sites available for γ-tubulin or whether recruitment itself is perturbed.**

We thank the reviewer for this insightful suggestion. To directly address this point, we quantified centrosomal CEP192 levels following RNAi. As shown in Fig. S1H–I, CEP192 is strongly depleted at centrosomes under siCEP192 conditions, arguing against the persistence of substantial residual CEP192 that could account for an intermediate phenotype. Regarding the possibility that the observed phenotype arises from impaired γ-tubulin recruitment via CEP192, our new RNAi complementation data with a WT Cep192 and CEP192 2A mutant that cannot bind Plk1, we show that Cep192 affects centrosome maturation via two mechanisms: via Plk1, and in a Plk1-independent manner, most likely due to its ability to bind γ-tubulin. This is now highlighted both in our results and in our discussion: "In contrast, the Cep192 2A mutant that cannot interact with Plk1 partially restored the recruitment of those pericentrosomal components (Fig. 7G-I), indicating that Cep192 contributes to PCM assembly both in a Plk1-dependent and independent manner."

10. **Figure S6 is referred to as Figure S7 in the text on line 175.**

We thank the reviewer the comment, we apologise for this labelling mistake. we have now corrected it in the revised version.

11. **Line 193-194: RPE1 CENEXIN-/- knock-out cells (to avoid applying a technically challenging triple siRNA treatment). Why only use this knock out for the imaging? The authors have been successfully using triple depletion throughout the manuscript, confirmed by western blotting in Figure S1G. This makes the rationale provided somewhat obsolete and the experiment is then inconsistent with the bulk of experimental work within the paper.**

We thank the reviewer for raising this point and agree that consistency of experimental systems is important. The use of RPE1 CENEXIN$^{-/-}$ cells in this specific experiment was motivated by technical considerations related to expansion microscopy. This approach substantially reduces the effective imaging area per coverslip following gel expansion, making the identification of appropriately staged cells (in particular G2-phase cells) considerably more challenging. In the context of triple siRNA depletion, where cell cycle progression is delayed and cell numbers are often reduced to the general toxicity of multiple siRNA, this limitation becomes a significant bottleneck, rendering the experiment technically impractical. To overcome this, we employed the CENEXIN knockout line as a simplified and robust system that phenocopies the relevant aspects of the triple depletion while allowing efficient acquisition of sufficient numbers of cells for quantitative analysis under expansion microscopy conditions. Importantly, our data throughout

the manuscript indicate that CENEXIN does not play a major role in the transitions examined here, supporting the validity of this substitution. Thus, the use of the knockout line represents a practical compromise for this technically demanding experiment rather than a conceptual deviation from the main findings. We have now clarified this rationale in the revised manuscript.

12. **Line 196-197: "We conclude that centriole disengagement is primarily driven by the centrosomal Plk1 activators Cep192 and Cenexin," Bora depletion causes ~90% of centrioles to be disengaged, which is reduced (albeit not entirely) by co-depletion or knockout of CEP192 and Cenexin. The authors conclude that the effects of Bora depletion are at least partially manifested through CEP192 and Cenexin. While the above statement is correct, this needs a much clearer explanation especially to non-experts given the highly penetrant effect of Bora depletion alone.**

    We thank the reviewer for the suggestion, and we now *explain the logic behind this result in a more explicit manner in our discussion, highlighting the role of partial Plk1 activity (page 15). "Rather, we propose that premature centriole disengagement instead arises from sustained Cep192 and Cenexin-dependent centrosomal Plk1 activity combined with reduced cyto/nucleoplasmic Plk1 activity during a prolonged G2 phase, consistent with our previous data showing that forcing cells to rapidly enter mitosis suppresses Plk1-dependent premature centriole disengagement in G2."*

13. **Line 270: "This differential regulatory network can… where the mitotic progression is delayed while the centrosome cycle advances, causing a severe desynchrony between the two otherwise synchronous processes." Is this regarding the increase premature centriole disengagement the authors observed? If so, it may be worth clarifying the statement as authors also demonstrated that during G2, centrosome maturation was also impacted by Bora depletion.**

    We thank the reviewer the comment and specified our thoughts in the following manner. "This differential regulatory network can, however, induce genetic instability, as exemplified Bora depletion, uncoupling the cell cycle and the centrosome cycle progression, where the mitotic entry is (retarded cell cycle) while the centriole disengagement is promoted (accelerated centrosome cycle) simultaneously, resulting in a pronounced desynchrony between the two otherwise synchronous processes"
